# A comprehensive survey on securing the social internet of things: protocols, threat mitigation, technological integrations, tools, and performance metrics

**DOI:** 10.1038/s41598-025-23865-4

**Published:** 2025-11-17

**Authors:** Deepa Ashok Patil, Shyamala G.

**Affiliations:** Department of Computer Science and Engineering, B.M.S College of Engineering, affiliated to Visvesvaraya Technological University, Bengaluru, 560019 Karnataka India

**Keywords:** Social internet of things (SIoT), SIoT security, Threat mitigation, Blockchain and edge computing, Artificial intelligence (AI) and Machine learning (ML) integration, Communication protocols, Engineering, Information systems and information technology, Mathematics and computing, Science, technology and society

## Abstract

The integration of social networking concepts with the Internet of Things (IoT) has led to the Social Internet of Things (SIoT)—a paradigm enabling autonomous, context-aware interactions among devices based on social relationships. While this connectivity improves interoperability, it also raises critical challenges in trust management, secure communication, and data protection. This survey reviews 225 papers published between 2014 and 18 September 2025, analyzing advancements in SIoT security. Sources include IEEE Xplore, ACM Digital Library, Springer, ScienceDirect (Elsevier), MDPI, Wiley, Taylor & Francis, and Google Scholar. Blockchain and AI/ML approaches feature prominently, with blockchain referenced in more than 50 papers, AI/ML in over 80, and many adopting both in combination. The literature is examined across architectural foundations, security requirements, and layered defenses, with evaluation most often based on latency, accuracy, scalability, and false-positive rate. The review further highlights existing security and communication protocols, attack mitigation strategies, and the adoption of blockchain, cloud, and edge computing for scalable and decentralized processing. The survey traces the evolution of SIoT research, identifies future directions to strengthen security and transparency, and serves as a reference for researchers and practitioners designing secure and decentralized SIoT environments.

## Introduction

**Figure 1:**
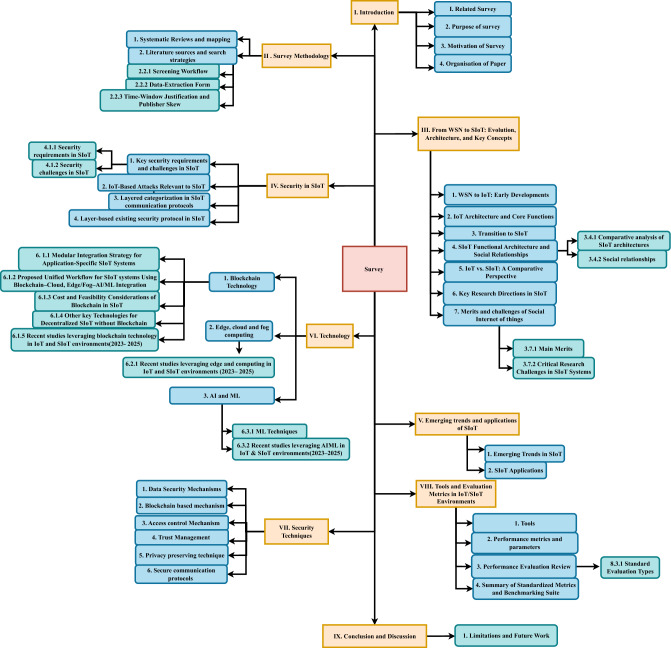
Tree-based taxonomy of surveyed research.

The integration of social networking with the Internet of Things (IoT) has led to the emergence of the Social Internet of Things (SIoT). This integration has brought about various security and privacy challenges that need to be addressed to ensure the safety of users and their data. The core aspects of SIoT include integrating social principles into IoT devices, ensuring seamless interoperability, enabling systems to operate independently, establishing trust and security, and enhancing context awareness. These elements foster interactions between devices, people, and services, creating a more connected and intelligent world. The increasing use of connected devices in critical applications has made security in SIoT an urgent and evolving area of research. While many studies have proposed security solutions for traditional IoT, the social dimension of SIoT introduces unique challenges related to trust management, privacy preservation, and secure communication. Addressing these challenges requires a comprehensive perspective that connects system architectures, communication protocols, attack mitigation techniques, and the emerging integration of enabling technologies such as blockchain for decentralization, cloud and edge computing for scalable performance, and artificial intelligence and machine learning for intelligent detection and decision-making. Although a few researchers have previously reviewed SIoT security, this survey is distinct in its integration-oriented perspective. It systematically covers SIoT literature from 2014 to 2025, with special emphasis on the most recent advances (2023–2025). Building on earlier reviews, our contribution lies in synthesizing diverse results into a layered, taxonomy-driven framework (Fig. 1) that connects architectures, threats, defenses, technologies, and evaluation methods.

**Key Contributions (tied to RQs and Taxonomy in Fig.** 1**):**

**1. Mapping SIoT threats and defenses (RQ**3**, RQ**4, Section “Security in SIoT”): We consolidate IoT-based and SIoT-specific attacks into a layer-wise classification across communication protocols, and map countermeasures into a structured defense taxonomy.

**2. Trust-exploitation and relationship-aware perspective (RQ**3**, RQ**5, Subsection 3.4.2): We analyze how SIoT social relationships (OOR, CLOR, CWOR, POR, SOR) influence attack surfaces and trust exploitation, and provide a comparative table (Table 4) linking relationships with their security implications.

**3. Technology-integration taxonomy (RQ**6, Section “Technology”): We categorize recent advances in blockchain, edge/fog/cloud computing, and AI/ML for SIoT, highlighting their feasibility, integration workflows, and recent studies (2023–2025) that combine these technologies for decentralized and intelligent security.

**4. Application trends and emerging domains (RQ**5**, RQ**9, Section “Emerging trends and applications of SIoT”): We survey application-driven SIoT research in domains such as smart healthcare, logistics, industrial IoT, and transportation, emphasizing how emerging trends translate into real-world deployments.

**5. Security techniques (RQ**7, Section “Security in SIoT”): We organize data security, blockchain-based mechanisms, access control, trust management, privacy preservation, and secure communication protocols into a layered set of techniques adapted to SIoT environments.

**6. Evaluation metrics and tooling synthesis (RQ**8, Section “Tools and evaluation metrics in IoT/SIoT environments”): We collate tools (e.g., NS-3, iFogSim, Ganache) and performance metrics (latency, scalability, trust accuracy, energy overhead), and review how they have been applied in SIoT evaluations. This provides a foundation for benchmarking and reproducibility.

**7. Gap analysis and future directions (RQ**9, Section “Conclusion and discussion”): We identify unresolved challenges in scalability, privacy preservation, explainability, and cross-layer security, and propose directions for research beyond 2025.

### Related survey

Several surveys have been conducted to review the security of Social Internet of Things (SIoT), and their findings are summarized in Table 1. The works by^1^ and^2^ provide the most comprehensive coverage, strongly addressing core areas such as requirements, attacks, applications, protocols, and security techniques. Reference^2^ further explore technology integration and performance evaluation, particularly within trust management systems. Papers like^3^ and^4^ address a wide range of topics with a focus on resource discovery and false service advertisement, respectively, offering partial insights into integration and tool support. Studies such as^5^ focus more narrowly on SIoT architecture but provide moderate consideration across multiple dimensions. Other contributions such as^6^ and^7^ highlight specific attack scenarios (e.g., malicious code injection and decentralization), but only partially cover broader evaluation metrics. The paper by^8^ emphasizes social relationships, offering strong insights into applications and parameters but lacking in protocol and integration discussions. Overall, while some papers offer comprehensive evaluations, others focus on specialized issues within the SIoT ecosystem, highlighting the fragmented but evolving nature of security research in this domain.

Scoring was performed manually by the first author and cross-checked by the research supervisor to ensure consistency. Each column indicates the extent to which the surveyed paper addressed the attribute: ✓ (explicitly covered), ★ (partially covered or indirectly addressed), and ✗ (not covered). Coding was based on full-text assessment of all included surveys using a standardized template. While the rubric was consistently applied, some interpretive subjectivity may remain, which we acknowledge as a limitation. Reference numbers (Ref. No) in the first column map directly to the corresponding citations in the bibliography. “Evaluation tools” refers to explicit use of simulation or testbed frameworks (e.g., NS-3, OMNeT++, iFogSim, TensorFlow), and “Performance evaluation parameters” refers to measurable system metrics (e.g., latency, throughput, scalability, trust accuracy, false positive rate, energy consumption, availability). The detailed scoring sheet is provided as **Supplement S2** (table1_related_surveys.csv) for transparency and reproducibility.

**Table 1:** Related surveys.

Ref. no	Year	Security requirements	Attacks	Applications	Security protocols	Existing security technique	Integration of technologies	Evaluation tools	Performance evaluation parameters	Aspects considered
^1^	2018	✓	✓	✓	✓	✓	★	✗	✗	Existing IoT architectures
^8^	2019	✓	✓	✓	✓	✓	★	✓	✓	Social relationships
^3^	2020	✓	✗	✓	✓	★	★	✗	✓	Resource Discovery
^4^	2023	✓	✓	✓	✓	✗	★	✓	✓	False service advertisement
^6^	2023	✓	✓	✓	✓	★	★	✓	✗	Malicious code injection attack
^7^	2023	✓	✓	✓	✓	✓	✓	★	★	Review of decentralization
^9^	2023	✓	✓	✓	✓	✓	✓	★	★	Challenges and attacks in SIoT
^5^	2024	✓	✓	✓	★	✓	★	✗	★	SIoT architecture
^10^	2024	✓	✓	✓	✓	✓	★	✓	✓	Types of IoT attacks
^2^	2025	✓	✓	✓	✓	✓	✓	✓	✓	Trust Management
**Our survey**	2025	✓	✓	✓	✓	✓	✓	✓	✓	Provides a survey on all aspects listed in the table.

**Legend:** ✓ = Fully covered; ★ = Covered; ✗ = Not covered

### Purpose of survey

The purpose of this survey is to systematically review existing research on security mechanisms within the Social Internet of Things (SIoT) ecosystem. It aims to identify and categorize prior work based on key aspects such as security requirements, attack models, protocol support, trust and privacy techniques, technological integrations (e.g., AI/ML, blockchain, edge/cloud computing), tool usage, and performance evaluation practices. By benchmarking these studies, this survey highlights the limitations and research gaps in current solutions particularly the lack of unified, scalable, and context-aware security frameworks. This assessment not only clarifies the state of the art but also forms the foundation for positioning our proposed work as a novel and necessary contribution to secure SIoT design.

### Motivation of survey

The increasing deployment of smart devices and their autonomous interactions in the Social Internet of Things (SIoT) has raised critical concerns around trust, privacy, security, and interoperability. Despite a growing number of research efforts addressing specific security challenges, existing surveys often focus narrowly on individual techniques or layers, lacking a comprehensive, multi-dimensional view that considers both technical and social dynamics in SIoT environments. Moreover, with the rapid evolution of enabling technologies—such as blockchain, edge/fog computing, and AI/ML—there is a pressing need to re-evaluate how these integrations affect security architectures. This motivates a structured survey to bridge fragmented knowledge, uncover gaps in current solutions, and guide the development of more robust, scalable, and decentralized security mechanisms tailored for next-generation SIoT systems.

### Organisation of paper

**Figure** 1 illustrates the taxonomy of this survey. **Section Summaries:**

**Section** “Introduction”**:** Introduces the study and its motivation.

**Section** “Survey methodology”**:** Details the systematic review methodology, including sources, search criteria, and inclusion/exclusion strategies ensuring transparency and coverage.

**Section** “From WSN to SIoT: evolution, architecture, and key concepts”**:** Reviews the evolution from WSNs to IoT and SIoT. Highlights SIoT architecture, social object interactions, and distinctions from IoT, along with key research directions and challenges.

**Section** “Security in SIoT”**:** Examines SIoT security requirements and challenges (e.g., Sybil attacks, trust manipulation), mapped to protocol layers and associated defense mechanisms.

**Section** “Emerging trends and applications of SIoT”**:** Discusses emerging trends like decentralized trust and context-aware analytics. Explores SIoT applications across domains such as healthcare, transportation, and smart homes.

**Section** “Technology”**:** Covers enabling technologies—blockchain, federated learning, fog/cloud computing, and AI/ML—for secure, scalable, and intelligent SIoT systems, with recent research insights.

**Section** “Security techniques”**:** Surveys layered security techniques including encryption, access control, trust evaluation, privacy preservation, and secure communication protocols.

**Section** “Tools and evaluation metrics in IoT/SIoT environments”**:** Describes evaluation tools (e.g., NS-3, iFogSim, Ganache) and key metrics (latency, trust accuracy, scalability) for benchmarking SIoT systems using standardized testing frameworks.

**Section** “Conclusion and discussion”**:** This survey traced SIoT’s evolution from WSNs to socially aware, secure IoT ecosystems. It highlighted SIoT’s architecture, trust-centric interactions, and enabling technologies like blockchain, edge/fog/cloud computing, and AI/ML. While advancements support decentralization and intelligence, challenges remain—lightweight trust models, privacy, interoperability, and scalability trade-offs. Future research must address ethical, social, and regulatory concerns as SIoT expands into critical sectors.

## Survey methodology

### Systematic reviews and mapping

We adopted the systematic survey mapping methodology outlined by^11^ and^10^ to construct this comprehensive review, enabling a structured and reproducible analysis across diverse research domains. The following ten research questions, numbered **1–10** in this section and later referenced as **RQ1–RQ10** in the relevant sections, guide the remainder of this paper and form the basis of the systematic review and mapping process.

1. How has the Social Internet of Things (SIoT) evolved from traditional Wireless Sensor Networks (WSNs) and IoT, and what are the key differences in architecture and social interaction models?
2. What systematic approach has been adopted to collect, filter, and analyze relevant SIoT literature from 2014 to 2025, and how does it ensure transparency and reproducibility?
3. What are the key security requirements of SIoT systems, and what unique challenges arise due to their decentralized, dynamic, and socially driven nature?
4. What types of attacks are most prevalent in SIoT environments, and how are they mapped across communication protocol layers?
5. What are the emerging trends in SIoT research, and how are these trends reflected in real-world applications such as smart healthcare, transportation, logistics, and industrial IoT?
6. How are emerging technologies such as blockchain, edge/fog/cloud computing, and machine learning (ML) being integrated into SIoT systems, and what are their roles, benefits, and limitations in enabling secure, intelligent, and decentralized operations?
7. What core security techniques are used in SIoT systems—including encryption, access control, trust management, privacy preservation, and secure communication and how do they address the system’s unique vulnerabilities?
8. What tools and simulation environments are commonly used to model, simulate, and evaluate Social Internet of Things (SIoT) systems, and what performance metrics and validation parameters are adopted in recent research studies?
9. What are the persistent research gaps and future challenges in developing scalable, interoperable, and privacy-preserving SIoT systems?
10. What are the core security challenges in SIoT systems?

### Literature sources and search strategies

This section addresses **RQ**2. We queried IEEE Xplore, ACM Digital Library, Elsevier (ScienceDirect), SpringerLink, MDPI, Wiley, Taylor & Francis, and Google Scholar. The last search was **18 Sep 2025**; the window was **Jan 2014–18 Sep 2025**. A keyword strategy targeted SIoT security (e.g., “SIoT”, “security”, “authentication”, “access control”, “attacks”, “MQTT”), with database-specific strings given below.

**Figure 2:**
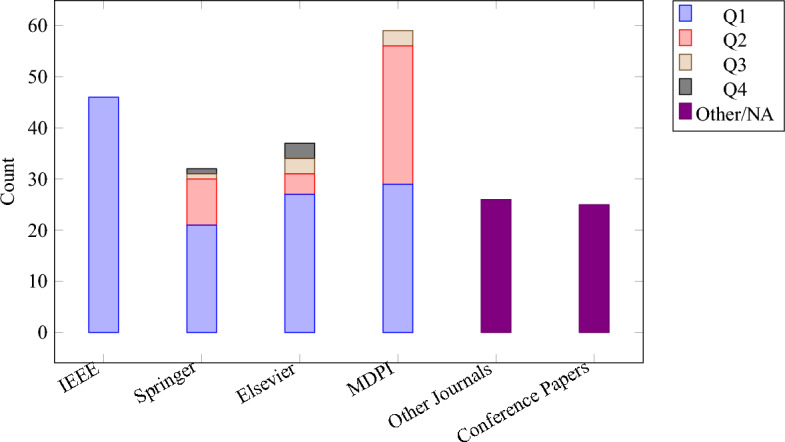
Publications by publisher and quartile.

**Figure 3:**
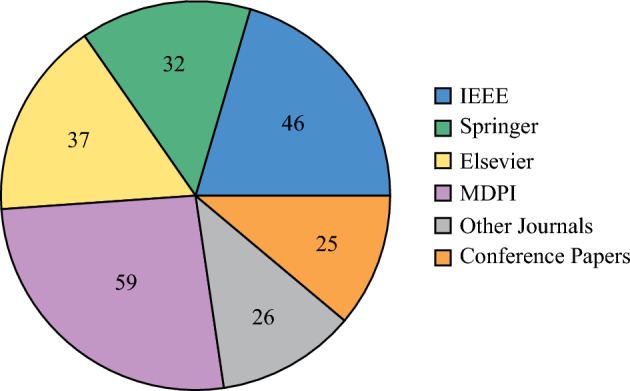
Publication share by publisher (n = 225).

**Figure 4:**
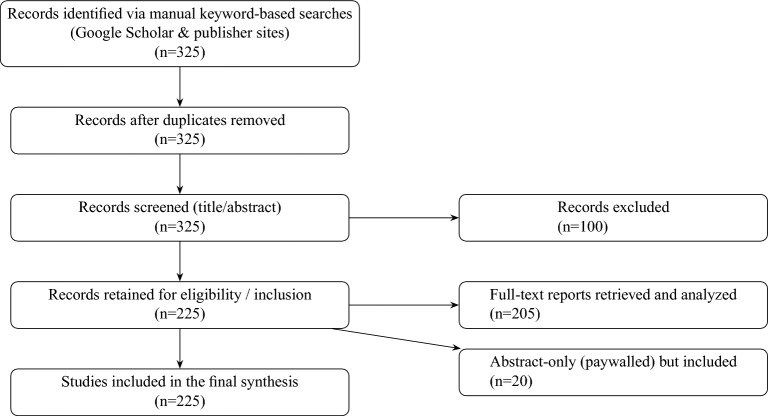
PRISMA flow diagram for study selection (search window: 2014–18 Sep 2025). All records were identified via manual keyword-based searches on Google Scholar and corresponding publisher sites.

**Selection summary (PRISMA).** We identified **325** records in total. After screening, **225** studies were included (**205** full-text and **20** abstract-only due to paywalls), and **100** were excluded at title/abstract. The full pipeline is shown in Fig. 4; publisher/quartile distribution and overall publisher share are shown in Figs. 2 and 3, respectively.

**Canonical query (semantics).**  (“Social Internet of Things” OR SIoT OR “social IoT”) AND (security OR privacy OR trust OR authentication OR authorization OR “access control” OR “intrusion detection” OR attack* OR threat* OR vulnerability OR “key management”) AND (IoT OR “Internet of Things”).

**Database-ready strings (examples).** 

- **IEEE Xplore (All Metadata):** ( “social internet of things” OR “SIoT” OR “social IoT” ) AND ( security OR privacy OR trust OR authentication OR “access control” OR “intrusion detection” OR attack* ) AND ( “Internet of Things” OR IoT ) Refinements: Year=2014–2025; Document Types=Journals, Early Access, Conferences.
- **ACM DL:**
  acmdlTitle:(“social internet of things” OR “social IoT” OR SIoT)
  AND (security OR privacy OR trust OR authentication OR “access control” OR “intrusion detection” OR attack*)
  Years: 2014–2025; Publication Type: Article, Proceedings.
- **SpringerLink / ScienceDirect / Wiley / T&F:** Title/Abstract/Keyword=(“social internet of things” OR “social IoT” OR SIoT) AND (security OR privacy OR trust OR authentication OR “access control” OR “intrusion detection” OR attack*). Years: 2014–2025; Content type: Journal Article, Conference Paper, Book Chapter.
- **Google Scholar:**
  “social internet of things” OR SIoT OR “social IoT”
  security OR privacy OR trust OR “access control” OR “intrusion detection” OR attack*
  Custom range: 2014–2025.

**Inclusion Criteria** Articles were included if they:

1. Addressed security in IoT with relevance to SIoT environments.
2. Proposed or analyzed effective security measures or protocols.
3. Discussed attacks, threats, or vulnerabilities in IoT/SIoT systems.
4. Covered security issues in IoT applications or communication protocols.
5. Focused on realistic or socially driven IoT use cases involving security.

**Exclusion Criteria** Articles were excluded if they:

1. Focused only on generic IoT security without SIoT relevance.
2. Purely theoretical work without security relevance.
3. Duplicates or secondary surveys.

#### Screening workflow

We used a two-pass pipeline aligned with PRISMA (Fig. 4):

1. **Pass-1 (Title/Abstract).** The first author screened the **325** records after deduplication. Borderline cases were flagged and independently checked by the supervising author; final decisions were made by consensus. This stage excluded **100** papers.
2. **Pass-2 (Full-text eligibility).** Of the remaining **225** studies, **205** were retrieved and assessed in full; **20** were included based on abstracts only due to paywalls but clear relevance. Disagreements were resolved by discussion; we did not compute a formal inter-rater statistic.

#### Data-extraction form

**Fields captured (systematically across all 225 studies):** Year; Title; Venue (publisher/journal or conference); Type (journal/conference/chapter); Access (OA/paywalled). We did not systematically code additional technical attributes (e.g., SIoT focus, security topics, protocols, metrics). A machine-readable CSV/JSON containing these five fields is provided in **Supplement S1** (corpus_225.csv).

#### Time-window justification and publisher skew

The **2014–2025** window captures the emergence and maturation of SIoT security: pre-2014 usage is sparse and terminologically inconsistent, while the chosen end date (**18 Sep 2025**) ensures currency. The publisher distribution (Figs. 2, 3) shows higher counts for IEEE and MDPI; this reflects (i) their larger throughput in IoT/SIoT and (ii) indexing coverage of our databases. To mitigate skew, we queried multiple publishers and platforms with uniform strings, deduplicated across sources, and retained venue-diverse evidence.

## From WSN to SIoT: evolution, architecture, and key concepts

This section comprehensively addresses **RQ**1 by tracing the progression from WSNs to IoT and then to SIoT, focusing on architectural developments and the emergence of social interaction models. It includes discussions on IoT architecture and core functions, the transition to SIoT, SIoT functional architectures, and various social relationship. A comparative analysis of IoT vs. SIoT architectures is also provided, along with key research directions, benefits, and challenges of SIoT. To support this discussion, Table 3 presents a synthesized review of existing SIoT architectures and their core functional components.

### WSN to IoT: early developments

**Figure 5:**
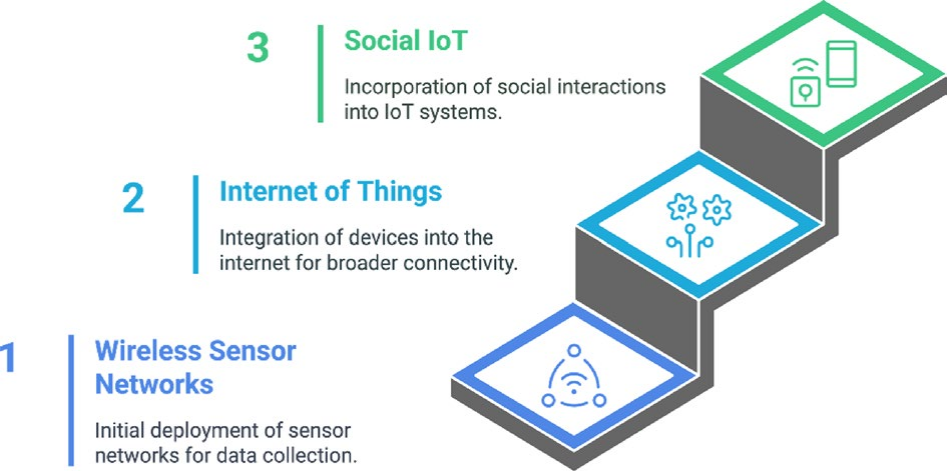
WSN to IoT and SIoT.

In our diverse society, social relationships are built on factors such as mutual interest, common goals, and shared resources, which help solve problems collaboratively. This idea of interconnectivity has also influenced the evolution of digital systems, transitioning from wireless sensor network (WSN) to Internet of Things and eventually to Social Internet of Things. As illustrated in Fig. 5, this evolution reflects a shift from simple sensing and data collection to more complex, socially aware systems. WSN consists of distributed nodes that monitor and collect data on environmental conditions such as temperature, humidity, and motion. These networks are typically limited to specific applications with limited communication capabilities and lack direct user interactions. However, they provide the functional data gathering layer necessary for more advanced systems. Based on the data collection capabilities of WSNs, IOT connects these physical devices to the Internet, enabling them to communicate, share data and perform actions based on the gathered information^12^. In IoT systems, devices are interconnected to provide improved automation, control, and monitoring in various sections from smart agriculture to industrial automation. However, interactions in IoT are mostly functional and lack the dynamic human-like social interactions seen in everyday human relationships.

### IoT architecture and core functions

**Figure 6:**
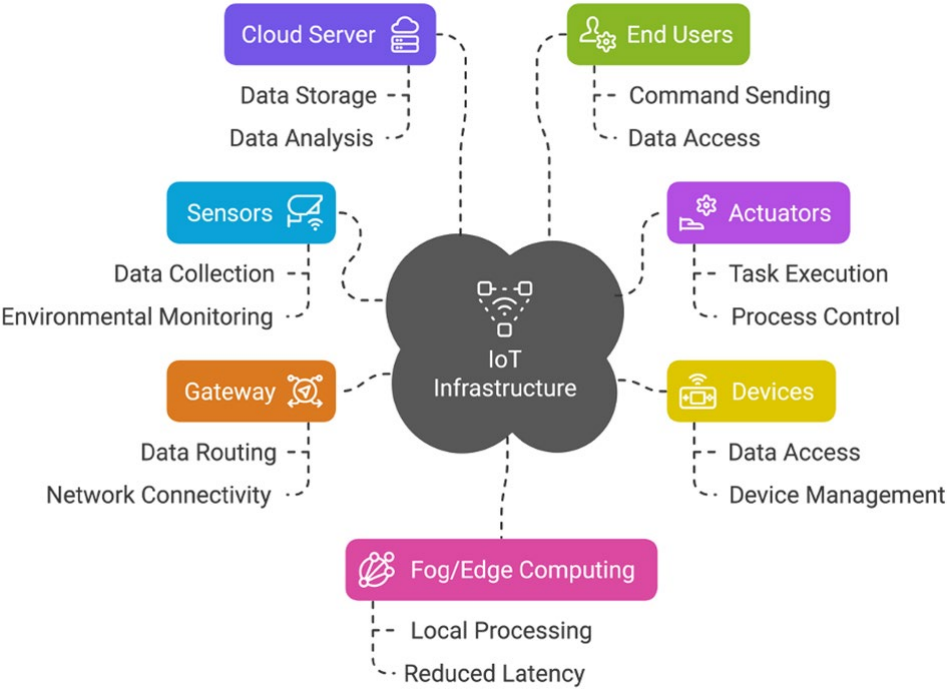
IoT infrastructure and communication.

The Internet of Things (IoT) is an interconnected network of various types of devices that communicate and interact seamlessly with each other over the Internet. At the core of IoT devices are sensors and actuators, which enable them to engage with the physical world and collect valuable data. Sensors detect and measure environmental changes, converting physical data into digital signals that can be analyzed and interpreted by IoT devices. Actuators, in contrast, convert digital signals into physical actions, receive commands from IoT devices, and execute tasks accordingly. As illustrated in Fig. 6, the IoT infrastructure consists of multiple interconnected components enabling seamless data flow and control. The Internet of Things (IoT) encompasses a wide range of devices, from common household appliances such as smart thermostats and wearables to industrial machinery and medical equipment. Although IoT devices offer numerous benefits, including enhanced convenience, improved efficiency, and data-driven insights, they also pose significant challenges related to security vulnerabilities, privacy concerns, and technical intricacies. Striking a balance between these advantages and disadvantages is essential for the effective and responsible implementation of IoT technologies. The applications of IoT are vast and wide-ranging, spanning multiple domains such as healthcare, finance, education, government, and beyond.

**Overview of IoT Functionality**  A typical IoT infrastructure includes sensors, actuators, devices, a gateway, and a cloud server. Sensors collect data that are transmitted to the cloud via a gateway for storage and analysis or to enhance through edge and fog computing. End users can access the data through endpoint devices and send commands, which are routed back to actuators to manage sensors or perform tasks. Although IoT devices are compact and compatible, they face challenges such as limited resources and security concerns. Various communication methods are used to ensure effective data exchange^13^. Various communication channels and methods are utilized to effectively facilitate data exchange between IoT devices.

**Figure 7:**
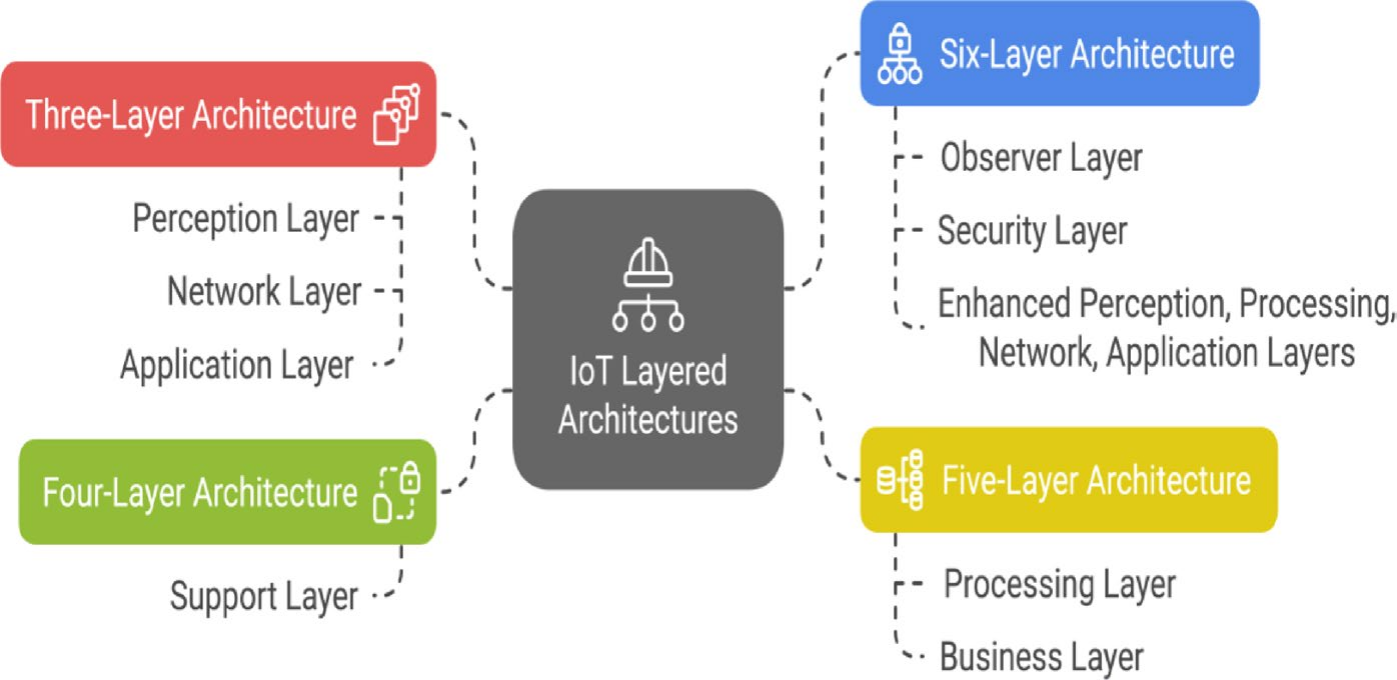
IoT layered architectures.

**Existing IoT architectures**  IoT systems are generally built using a layered approach, and the number of layers can vary depending on the complexity and requirements of the system. The most common IoT architectures are the three-layer, four-layer, five-layer, and six-layer architecture as shown in Fig. 7. To ensure the security of IoT systems, it is crucial to understand the layered architectures, the potential security threats at each layer, and the existing security measures designed to mitigate these threats. The IoT layered model has been discussed extensively in the literature, evolving from the three-layer baseline to four, five, and six-layer enhancements, each addressing security and scalability limitations^1^. In this study^14^ they provide a broad IoT survey, detailing standard architectures such as the 3-layer, 5-layer, and fog gateway models, emphasizing their roles in perception, processing, and service delivery. The paper also introduces the Social IoT (SIoT) concept, outlining its basic components (identification, metadata, security controls, discovery, relationship management, and service composition) and presenting a representative layering (server-side with three layers; device-side with object and social layers). However, this remains conceptual/illustrative, not a novel SIoT architecture. The work further highlights middleware requirements (privacy, trust, security) and QoS dimensions (availability, scalability, interoperability, dependability, performance, mobility), framing open challenges around DoS resilience, scalability, and trust management. Each evolution in the architecture introduces layers to address specific IoT challenges such as security, data handling, and scalability, culminating in the six-layer model for comprehensive protection and efficiency.

Three-Layer Architecture: The simplest model with three layers: Perception Layer: Gathers data from sensors. Network Layer: Transmits data to processing systems. Application Layer: Provides IoT services to users. Although foundational, it lacks advanced security features, making it vulnerable to attacks. Four-Layer Architecture: Builds on the three-layer model by adding a Support Layer between the Perception and Network layers. The Support Layer authenticates and verifies the data before passing it to the Network Layer, addressing security flaws in the three-layer model. Five-Layer Architecture: Expands the four-layer model with two new layers: Processing Layer: Handles data filtering, storage, and analysis to manage big data challenges. Business Layer: Oversees system management, user privacy, and business logic, addressing application-specific vulnerabilities and business-level threats. Six-Layer Architecture: Improves functionality and security further with these layers: Observer layer: Verifies data integrity and authentication from the Perception Layer. Security Layer: Encrypts data for secure transmission, mitigating risks at the Network Layer. Other layers (Perception, Processing, Network, and Application) remain, but are more robust, supporting better security, scalability, and service delivery.

### Transition to SIoT

The SIoT is the next evolutionary step after the IoT by introducing social, intelligent, and collaborative interactions between devices and users. In SIoT, devices can autonomously discover, select, and interact with other devices based on the social relationships they form, enhancing collaborations and functionality. Integrating the principles of social networking into the Internet of Things (IoT) introduces a new paradigm known as the Social Internet of Things (SIoT). While IoT connects physical devices over the Internet to collect and exchange data, SIOT goes beyond allowing these devices to interact with each other and with people in a social, human-like manner, like interactions on a social media platform. For example, SIoT can transform smart homes by learning occupants’ preferences and automatically adjusting the settings to improve comfort and efficiency. It enables smartwatches to share fitness data with friends or participate in group fitness challenges. A smartwatch might collaborate with a smart refrigerator to suggest healthy food options based on fitness goals. Smart cars can share real-time traffic updates with each other, optimising routes and reducing congestion. In smart cities, streetlights can share energy usage data to balance power consumption. Devices can verify the creditability of each other before sharing sensitive data, reducing the risk of malicious attack. It also enhances entertainment by facilitating applications that share movie reviews and suggest content based on a user’s past preferences and enable the sharing of music playlists with friends and family. Incorporating SIoT into our daily life offers significant advantages, such as enhancing user experience, intelligent decision-making, social and collaborative interactions, scalability and resource optimization, security and trust management, and unlocking new business opportunities in a wide range of applications.

**Figure 8:**
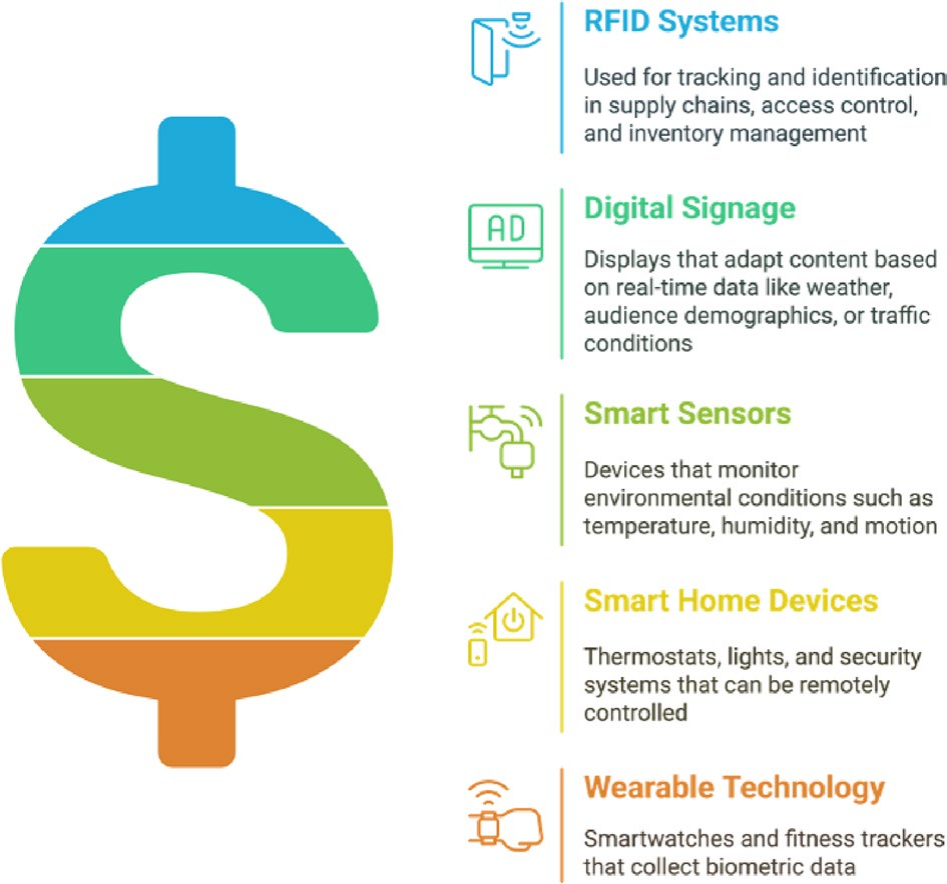
Overview of smart objects.

**Smart Objects:** Smart objects are physical devices embedded with microcontrollers, sensors, and actuators that enable them to collect, process, and share data autonomously over the Internet. Figure 8 shows the overview of smart objects.

### SIoT functional architecture and social relationships

This subsection presents a brief discussion of our proposed conceptual functional framework for SIoT. In addition, we also review recent research contributions from other researchers on advances in SIoT architecture.

**Figure 9:**
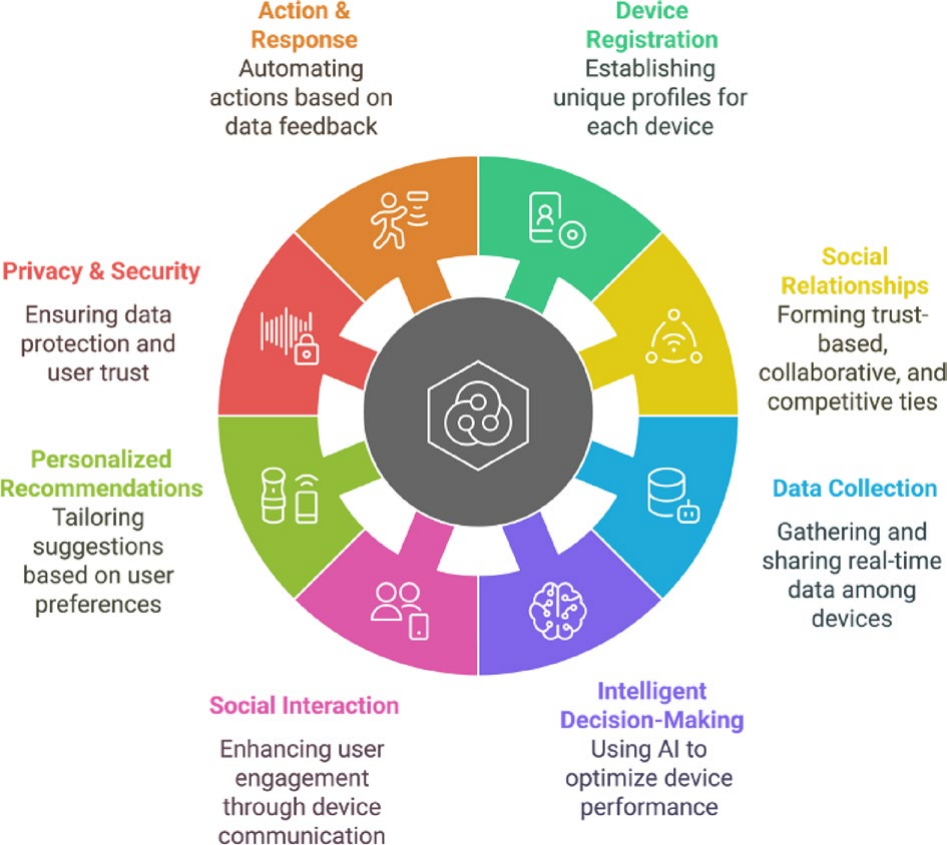
Conceptual functional framework of the social internet of things (SIoT).

**Figure 10:**
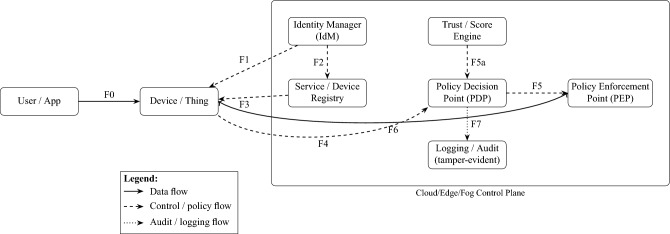
Functional SIoT framework with labelled interfaces (F0–F7).

**Figure 11:**
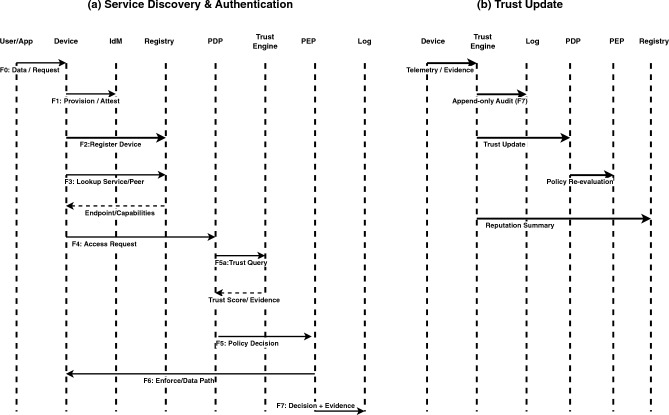
Conceptual SIoT sequence diagrams. (**a**) Service Discovery and Authorization, showing provisioning, registry lookup, trust evaluation, and policy enforcement. (**b**) Trust Update, showing evidence logging, trust updates, policy re-evaluation, and reputation sharing.

This conceptual wheel framework is literature-based (no implementation claim) and integrates recurring elements highlighted across surveyed SIoT architectures. For instance, service and data discovery features identified in prior works^5,15,16^ are implicitly captured in the Device Registration and Data Collection components. Similarly, trust-centric modules^17,18^ are reflected in Social Relationships and Privacy & Security. Together, Figs. 9 and 10 provide a synthesized conceptual model abstracted from comparative analysis: Fig. 10 captures the high-level functional elements, while Fig. 11 illustrates the corresponding sequence interactions. Figure 9 Conceptual functional architecture of the Social Internet of Things (SIoT). The diagram synthesizes eight core functions—Device Registration, Social Relationships, Data Collection, Intelligent Decision-Making, Social Interaction, Personalized Recommendations, Privacy & Security, and Action & Response—highlighting how SIoT couples social ties with device capabilities. Conceptual illustration; literature-based (no implementation claim).This architecture provides an overview of the functional components of the SIoT architecture which consists of various components such as Device Registration, Social Relationships, data collection, Intelligent Decision making, Social Interaction, Personalized Recommendation, Privacy and security, and Action and response. Each of these has their own functionalities, while they work together to provide autonomous intelligent decision-making entities that can connect, collect data, share, and seamlessly communicate between users and other devices over the Internet within the framework. In the SIoT network, devices such as sensors, wearables, and smart home appliances would be able to create unique digital identities. These devices form a connection based on shared goals, built on trust, collaboration, or competition. These IoT devices collect real-time data, processing it locally (edge computing) or storing it in the cloud to improve the interactions between users, devices, and other devices within the SIoT network. Emerging technologies such as advanced AI and machine learning algorithms that support intelligence decision making, allowing devices to adopt to user preferences. They also facilitate social interactions by sharing data across networks, providing personalized recommendations, and instant feedback. To ensure security and privacy, robust authentication protocols be implemented, restricting access to sensitive information collected from devices and users, to further ensure that they can also consider leveraging blockchain technology. Devices perform automated actions based on gathered data, such as adjusting home settings or sending alerts, and provide users with post-event summaries to monitor performance and share insights. This figure is conceptual and distill patterns reported across prior SIoT literature.

Figure 10 refines this view into a functional workflow, represented through labelled interfaces (F0–F7), separating device-level interactions from control-plane functions. The glossary embedded in the caption specifies each interface. This conceptual framework emphasizes the role of identity, trust, and policy enforcement as recurring patterns across surveyed works. A user/app sends data to a device (F0). The device is provisioned and validated by the Identity Manager (IdM), which also updates the Service/Device Registry (F1–F2). The registry answers discovery lookups back to the device (F3). Before execution, the device requests an authorization decision from the Policy Decision Point (PDP) (F4). The PDP incorporates trust evidence from the Trust/Score Engine (F5a) and sends its verdict to the Policy Enforcement Point (PEP) (F5). Authorized data traffic is then enforced on the PEP–device path (F6). All control decisions are audited (F7) to support accountability and forensics. The container indicates that these functions may be deployed in cloud, fog, or edge infrastructure while retaining the same conceptual flow. This conceptual framework emphasizes the role of identity, trust, and policy enforcement as recurring patterns across surveyed works. Table 2 complements this workflow by mapping each interface (F0–F7) to specific cryptographic operations and their overheads. Device provisioning (F1) relies on asymmetric key lifetimes, session initiation (F0) employs TLS/DTLS traffic keys or PUF-based derivations, and registry lookups (F2–F3) are anchored with signed keys. Authorization (F4) introduces PDP evaluation costs, while enforcement at the PEP (F5–F6) depends on signed tokens and symmetric encryption with negligible latency. Trust evidence (F5a) and audit logging (F7) leverage event signatures and blockchain anchoring, introducing modest but acceptable delays. Together, Fig. 10 and Table 2 capture how cryptographic defaults underpin the SIoT control workflow.

**Interface glossary.** F0 = User/App $\rightarrow$ Device/Thing (data initiation); F1 = Identity provisioning; F2 = Device registration; F3 = Service discovery; F4 = Access request (to PDP); F5 = Decision (PDP$\rightarrow$PEP); F5a = Trust query (to Trust Engine); F6 = Enforcement/data path (PEP$\leftrightarrow$Device); F7 = Append-only logging/audit.

**Table 2:** Cryptographic key lifetimes and performance overheads (conceptual defaults, grounded in surveyed SIoT literature).

Component/operation	Material/mechanism	Lifetime/overhead
Device identity	Ed25519 / ECDSA-P256 keypair; finite-field schemes ^19^	6–12 months (or on reprovision)
Channel session	TLS/DTLS traffic keys (HKDF); PUF-based derivation ^20^	Per connection / transaction
PEP auth token	Signed policy token	5–15 minutes rolling refresh
Trust evidence	Event signature (Ed25519)	Per event (immutable)
Storage at rest	AES-GCM symmetric data keys	24h rotation (envelope rewrap)
Audit/anchoring	Blockchain anchoring keys ^21^	1–3 s commit delay; hourly batching
Registry signing	Service registry key ^22^	3–6 months with audit log
PDP policy check	Fog-node policy evaluation	20–50 ms (delegation selection overhead) ^23^
PEP enforcement	Policy decision enforcement	<5 ms (message interception + enforcement negligible vs PDP) ^23^
AES-GCM encryption	Symmetric crypto cost (AES-GCM; lightweight ciphers such as SIMECK-T ^24^)	<5% CPU
Trust verification	Trust/score update	2.2 s, 280 Tx/s ^25^
Blockchain logging	Append-only tamper-evident logs	1–3 s per commit ^26^

Figure 11 illustrates the SIoT process through sequence diagrams. Part (a) outlines service discovery and authorization: a device is provisioned, registered, and queries the registry; requests are evaluated by the PDP with evidence from the trust engine; final decisions are enforced through the PEP and recorded in the log. Part (b) shows the continuous trust update cycle: devices send telemetry to the trust engine, which appends audit logs, updates the PDP for potential re-evaluation, and publishes reputation summaries to the registry. These interactions highlight both the operational and feedback loops necessary for sustaining trust-aware SIoT environments.

#### Comparative analysis of SIoT architectures

Although there is no standard form of an architecture, researchers have proposed their own architecture based on the underlying concepts of IoT with the SIoT principles. Each evolution in the architecture introduces new layers to address specific SIoT challenges such as device and data service discovery, security, data storage and analysis, interoperability, and scalability. Several researches have proposed new SIoT architectures that integrate social elements into IoT in a cohesive and efficient manner. These architectures aim to enhance device interactions by incorporating social networking principles into the IoT, enabling devices to form and manage social relationships autonomously.

The^16^ general architecture of SIoT consists of three layers, such as 1. Perception layer: The perception or sensing layer which is responsible for sensing from the physical environment and collecting data through various sensing devices such as RFID, Sensors (Temperature, Humidity, Pressure, Motion, etc.) IoT enabled cameras, GPS, NFC. 2. Network Layer: To establish communication and ensure reliable transmission of data between various devices and cloud system, various network elements can be employed, including: Cellular networks, WLAN, LoRaWAN, wireless personal area network can be used. 3. Application layer: This layer serves as a user interface between end users and IoT, offering a variety of services that facilitate enhanced connectivity, automation, and decision making. Services such as SIoT application services (Industrial monitoring, Smart home), Service Discovery, Service management, Data storage, and Database Services.

They^5^ proposed an architecture pattern for SIoT that aligns with a broad spectrum of requirements, incorporating fundamental elements, such as SIoT Service Discovery: Devices that autonomously discover services that meet its specific needs, acting as gateway for inter-object relationship, Social Virtual Entity (SVE): Storage for a social virtual entity that represents digital information of real-world physical objects, SVE resolution: It provides essential information such as SIoT IDs, service types, and location details that need to connect to Social Virtual Entity with SIoT services, which enable them to access relevant services and information, Relationship management: To track and evaluate how devices interact, exchange information and to ensure that they follow predefined behavior. This helps decide who to trust and work with based on past experience, relationship behavior, and monitoring. If a device behaves in a certain way, then the system ensures that it aligns with expected behavior and adjusts when needed. It also checks the system, consciously observes device interaction, and flags if any problems occur.

The^15^ proposed Semantic Web of Things (SIoT) architecture consists of multiple layers. The bottom layer comprises embedded devices and IoT technologies for smart cities, where sensors collect data and transmit it through UDP / IP or CoAP. The U-KB layer annotates and represents knowledge, structuring received data using Linked Data principles. The annotation of metadata is stored using OWL-2 ontologies, enabling structured representation. The Tiny-ME Rationality and Matchmaking Engine employs semantic Rationality for Service Discovery, processing requests accordingly. The top layer facilitates service discovery and resource discovery within the SIoT ecosystem, incorporating intelligent decision-making based on available resources. This architecture integrates annotation based on ontology, enabling efficient data representation and communication between IoT devices. Using semantic reasoning, you can easily discover services and resources. By combining COAP, Linked Data, and UDP/IP, the SIoT architecture ensures reliable, interoperable, and efficient IoT communication.

The S2NeTM architecture, as described in^18^, is a distributed middleware solution that leverages semantic technologies to seamlessly interconnect IoT devices, optimizing data processing and facilitating informed decision-making through ontology-based semantic reasoning. The architecture consists of three layers: the Data Collection Platform, which comprises physical devices that detect and perceive data from IoT devices, sensors, and open data sources, and handles communication protocols such as MQTT, COAP, and HTTP; the S2NeTM middleware, responsible for data processing, semantic reasoning, and trustworthiness management through components such as CM, OC, UP, and TM; and the Application Layer, which provides services and interfaces to users. The effectiveness of this middleware architecture has been demonstrated through a successful implementation of the Green Route Use Case, where users receive eco-friendly route recommendations based on real-time environmental data.

As described in^17^, a socially aware service recommendation framework is proposed for the Social Internet of Things (SIoT). This framework considers devices, their owners, and the services offered by these devices. The framework operates in four stages. Initially, social relationships among devices are identified based on five types of SIoT relationships (CLOR, CWOR, POR, OOR, SOR) derived from their owners’ social connections. The devices are then clustered into communities based on their social relationships using a boundary-based community detection algorithm. Within these communities, users are grouped by common preferences and behaviors using the Jaccard similarity coefficient, enabling interest-based service suggestions. Finally, a hybrid filtering approach (collaborative and content-based filtering) is adopted for service recommendation, prioritizing trustworthy and relevant services based on user-device relationships, interest similarities, and social connectivity strength. The performance of the framework is evaluated using real-world datasets (Santander Smart City and Twitter), with metrics including precision, recall, F measure, and computational cost.

**Table 3:** Summary of SIoT architectures and core features.

Ref	Year	Publisher	SIoT Architecture	Core Aspects of SIoT				FT	Fog/CD	BCT	Type	Limitations
				NN	SD	RM	TM					
^15^	2024	MDPI	Integrated SIoT–SWoT using LDN, LDP-CoAP, OWL, and Tiny-ME	✓	✓	✓	partial	✓	✓	✗	Simulation	No blockchain; edge-only; needs semantic infra
^5^	2024	MDPI / Mathematics	Proposed architecture pattern for SIoT	✓	✓	✓	✓	✓	✓	✗	Survey	Only a high-level concept
^18^	2023	MDPI	S2NeTM architecture & operational environment	✓	✓	✓	✓	✗	✗	✗	Implemented	No FT, fog/CD, BCT; few metrics; static privacy
^17^	2021	IEEE	Social-Relationships-Based Service Recommendation System for SIoT Devices	✓	✓	✓	✓	✓	✗	✗	Impt and sim	High cost for large-scale community detection
^27^	2020	Springer	Five-Layer architecture for SIoT	✓	✓	✓	✓	partial	partial	✗	Survey	Descriptive only; lacks implementation
^28^	2019	IEEE	Layered SIoV architecture	✓	✓	✓	✓	✗	✓	✓	Survey	No implementation or experiments
^12^	2014	IEEE	Generic Social Internet of Things (SIoT) architecture	✓	✓	✓	✓	✓	✗	✗	Survey	Lacks integration with fog, AI, BCT
^29^	2012	Elsevier	three-layer SIoT architecture	✓	✓	✓	✓	✗	✗	✗	Survey & Conceptual + Simulated	Simulation only; no real-world testing

NN = Network Navigability, SD = Service Discovery, RM = Relationship Management, TM = Trust Management, FT = Fault Tolerance, Fog/CD = Fog/Cloud Computing, BCT = Blockchain. ✓= Present, ✗= Absent, “partial” = Partially addressed.

They^27^ presented a five-layered SIoT architecture by introducing two new layers: the component abstraction layer, which provides object profiling, and the social interaction layer, which provides an interface for social communication between objects and users. The functionality of the remaining layers is similar to that of the IoT reference architecture.

In this paper^29^ they proposed comprises of a three-layered architecture, the SIoT server focuses on the network and application layer to ensure efficient data management, relationship, and service discovery. The network layer is responsible for transferring data across different networks to enable communication among various devices within the IoT, interoperability, and protocol adherence. Each of these sub-layers of Application layer, that is Base, Component, and Interface provide different functionalities such as managing database for social profiles, object activities, and human data. Ontologies such as OWL-S can be used to represent semantic relationship, while component sublayer implements core functions such as ID management, Profiling, Owner control, Relationship Management, Service Discovery, Service composition, and Trustworthiness Management. The top Interface sublayer provides functionalities to connect to the third-party entities to objects, it can be either human/ services, which can enable flexible implementation (local, distributed, or cloud-based). The Gateway and object they vary in their combination of layers based on device capabilities. This SIoT architecture fosters interoperability, semantic understanding, and autonomous service discovery among IoT devices. The Social Internet of Vehicles (SIoV)^28^ architecture is a complex framework that facilitates communication and data exchange between vehicles, infrastructure, and the cloud, allowing a variety of services and applications. At its foundation, the Physical World Layer collects data from various sensors and devices, while the Gateway Layer acts as an immediate point of communication, comprising the Smart Vehicle module and the Roadside Unit. The Fog layer processes larger data sets, analyzing data locally at the edge of the network, and the Cloud Layer is a centralized, remote facility optimized for robust computations and long-term data management. The Application Layer provides a variety of services and interfaces to users through applications running on vehicles or in the cloud, handling real-time and historical data while balancing performance and capacity, and raising privacy concerns due to data sharing with third-party entities According to^12^, this architecture consists of four components such as actors, users, and smart devices that interact via social network, providing services such as status updates and recommendations, The intelligent system which controls interactions, and managing services. The interface layer enables user- device interaction which offers services like discovery and data analytics. The Internet ensures global connectivity, open access, and real-time communication between SIoT devices. Table 3 shows the comparative analysis of SIoT architectures.

#### Social relationships

Social relationships are formed through shared interests that lead to communities that collaborate and share information. For example, a book club brings together individuals who enjoy reading and discussing literature, fostering friendships and knowledge exchange among members. The Internet connects these communities locally and globally, fostering belonging and cooperation. This interconnectedness forms the Social Internet of Things (SIOT), where people, devices, and services interact to create a collaborative ecosystem. Various studies^8,30^ have examined different types of social relationships between objects and their users, which are consolidated in Table 4.

**Table 4:** SIoT relationship types: definitions, examples, and security implications.

Relationship type	Semantic definition	Example	Security implications (attack surface & trust signals)
Ownership Object Relationship (OOR)	Objects continue to interact despite ownership changes	Smart car retaining traffic data after resale	Data leakage risk across owners; requires secure data wiping, provenance, and access control
Social Object Relationship (SOR)	Objects interact via owners’ social connections	Friends’ fitness wearables syncing stats	Vulnerable to impersonation or Sybil attacks; trust inferred from social graph strength
Sibling Object Relationship (SIBOR)	Objects owned by the same user communicate frequently	Smart home devices (thermostat, lights, sensors)	Lateral compromise risk; trust derives from shared owner identity and credentials
Parental Object Relationship (POR)	Sibling devices connected via a parent entity	Fleet of connected vehicles	Centralized control introduces single point of failure; parent trust determines child reliability
Co-location Object Relationship (CLOR)	Objects interact due to spatial proximity	Factory robots working together	Susceptible to spoofed/relay attacks; trust based on verified physical presence
Co-Work Object Relationship (CWOR)	Objects collaborate to complete a task	Robotic arms and conveyors in packaging	Attack surface in coordination sabotage/DoS; trust validated through task success consistency
Guest Object Relationship (GOR)	External objects interact with restricted access	BYOD devices in enterprise	Higher risk of rogue devices; needs strict authentication, sandboxing, and access policies
Stranger Object Relationship (STGOR)	Limited interactions with unknown devices	Unknown IoT object in range	High uncertainty and unpredictability; requires anomaly detection and adaptive trust mechanisms
Service-Oriented Object Relationship (SVOR)	Objects interact with external service providers	Smart meters subscribed to weather/utility services	Exposure to API/service misuse; trust depends on authentication, SLA compliance, blockchain logging

### IoT vs. SIoT: a comparative perspective

The Internet of Things (IoT) is a network of physical devices connected to the Internet that collect and exchange data. IoT features include device communication with servers or cloud platforms, focusing on machine-to-machine interaction, centralized control, and security trust models based on authentication and encryption. The Social Internet of Things (SIoT) expands the IoT by enabling devices to interact in a social-like manner, similar to human social networks, emphasizing peer-to-peer relationships, trust-based collaboration, and decentralized smart device cooperation.

### Key research directions in the SIoT

This section addresses **RQ**9. The aforementioned challenges have sparked diverse research efforts. This section highlights key directions addressing those issues, as explored in recent literature.

**Service Discovery**  Service discovery is the process by which a device or user in a network finds and connects to services offered by other devices or systems automatically and dynamically. For example, consider a vending machine application installed on your smartphone. This automatic finding and matching process is service discovery—your app is discovering services (snack vending) being offered by a device (vending machine); it detects nearby vending machines (using Bluetooth, Wi-Fi, GPS), once connected to that specific machine, it shows available items (chips, juice, chocolate, etc.). To ensure trustworthy and efficient service discovery in SIoT^31^, proposed a three layered model that combines social trust and QoS prediction. In this study, the authors^32^ proposed and implemented a decentralized service registry built on the DSF-IoT framework and a S/Kademlia-based Distributed Hash Table (DHT). The approach ensures integrity and trust through the use of signature chains and cryptographically derived identifiers. Service registration and discovery are facilitated through tertiary pages, which support context-based queries and enable efficient, verifiable indexing. Despite these advances, most current approaches still risk exposing sensitive identity or location attributes during discovery. Future protocols must therefore emphasize *privacy-preserving service discovery*, enabling secure interaction without requiring disclosure of user or device identities.

**Trust management**  Trust management has become a critical component of SIoT systems, aiming to ensure secure and reliable interactions among heterogeneous and socially connected devices. Reference^2^ emphasize that trust management mechanisms involve four core phases: trust composition, trust aggregation, trust propagation, and trust update. These phases help assess node behavior, detect malicious activity, and maintain dynamic trust scores within SIoT networks. The authors also propose a blockchain-powered methodology that integrates decentralized architectures with graph-based trust models (e.g., using Neo4j) to enhance scalability, transparency, and resilience against trust-related attacks. Their survey identifies key open challenges such as real-time trust updates, trust-related attack mitigation, and the integration of smart contracts and consensus protocols for trust evaluation. However, many existing schemes remain static or computationally heavy, which makes them unsuitable for resource-constrained SIoT devices. This highlights the need for *lightweight, adaptive trust strategies* that can dynamically evolve with device behavior while maintaining low computational overhead.

**Network Navigability**  Network navigability refers to the ability of devices to efficiently discover and connect with relevant peers or services within a dynamic social graph. Challenges such as device mobility, evolving contextual dependencies, and spatiotemporal heterogeneity often degrade navigability in large-scale SIoT networks. To tackle this, TAGLLM^33^ introduces a trajectory framework that models devices dynamics and contextual relations using a hybrid graph encoder and LLM guided token alignment strategy, significantly improving relation classification and routing performance.

**Relationship management**  In SIoT, relationship management refers to how smart devices establish, maintain, and update social-like relationships with each other similar to human social networks. The relationships often managed in SIoT are OOR, CLOR , CWOR, SOR, and POR. RM ensures reliable, secure and meaningful interactions between devices by continuously assessing trustworthiness and context. To address trust exploitation and privacy leakage, they^34^ proposed an F-TRM model that classifies relationships and updates them dynamically using trust values and feedback and secures interactions using cryptographic methods. They also demonstrate effective relationship evolution and management in dynamic environments.

**Explainable and accountable AI/ML integration**  Machine learning (ML) has been widely applied to SIoT for anomaly detection, trust prediction, and adaptive service management. However, a major limitation is the lack of transparency in ML-driven decisions, which hinders accountability and user trust. Most existing solutions function as black boxes, providing little insight into why a node or service is flagged as malicious or untrustworthy. To bridge this gap, explainable AI (XAI) techniques such as SHAP and LIME should be integrated into SIoT security workflows. These methods can provide human-understandable justifications for anomaly detection or trust evaluations, improving confidence and enabling fair decision-making in multi-stakeholder environments. Incorporating explainability into SIoT ML pipelines is crucial for accountable security and ensuring that automated trust management aligns with user expectations and regulatory requirements.FedXAIIDS is an intrusion detection system that combines federated learning with SHAP-based explainability to preserve privacy and improve transparency. Tested on the CICIoT2023 dataset, it achieved  88% accuracy while identifying UDP traffic as a key attack feature, offering a scalable and interpretable IDS for IoT networks^35^. XAI-IoT, proposed by^36^, is a framework combining anomaly detection models with seven XAI methods (e.g., SHAP, LIME) to enhance transparency in IoT security. Evaluated on MEMS manufacturing and N-BaIoT botnet datasets, it achieves high accuracy (up to  0.99) and reveals key features for failures and attacks, supporting accountable and interpretable IoT anomaly detection.

**Cross-domain interoperability and unified policies**  One of the key open challenges in SIoT is ensuring seamless interoperability across heterogeneous domains (e.g., smart healthcare, transportation, and industrial IoT), where devices often follow distinct trust policies, access-control rules, and security protocols. The absence of standardized frameworks limits secure collaboration when nodes from different administrative or application domains interact. Current studies largely focus on intra-domain trust or discovery, leaving cross-domain integration underexplored. To address this, future efforts should aim at designing unified policy frameworks and interoperable trust models that allow heterogeneous SIoT systems to share resources and services securely while maintaining autonomy. Such frameworks should also incorporate decentralized enforcement mechanisms (e.g., blockchain-based access-control or distributed identity management) to guarantee auditability and resilience against policy conflicts.

### Merits and challenges of social internet of things

#### Main merits

1. **Enhanced Resource Discovery:** Enhanced resource discovery within the social Internet of Things (SIoT) identifies a sophisticated, context-aware, and socially influenced approach to locate pertinent resources, including services, data and other devices, in a dynamic IoT environment. By integrating social relationships, trust, semantic considerations, and contextual factors, it facilitates efficient, precise, and secure discovery. Resource discovery functions by identifying devices or objects, as well as the social relationships among these objects and their users-such as parental, co-location, and co-work relationships. Discovery methodologies can be classified into centralized, decentralized, or hybrid approaches. Various types of discovery include context-aware discovery, which is based on situational reading of data; trust-based discovery, which relies on past history, recommendations, and reputation scores; social-aware filtering, which considers relationship types; and semantic discovery and ontologies, which use semantic models (RDF and OWL) based on meaning, not just keywords. Furthermore, machine learning may be employed optionally for predictive purposes, along with scalable and decentralized discovery methods, such as distributed hash tables or blockchains for scalable decentralized and resource discovery. This paper^3^ discusses various categories of discovery techniques, including data-based and object-based approaches. It also examines different types of discovery architecture and various protocols for discovery, such as CoAP, MQTT, and UPnP. Additionally, the paper addresses several challenges encountered in resource discovery.
2. **Enhanced Interoperability and Collaboration:**
  Interoperability refers to the capability of different types of objects, devices, or applications to work together to exchange information within the SIoT network seamlessly. This ensures that they can communicate and collaborate effectively by speaking the same “language.” For example, different healthcare hospitals using various software should be able to share information securely, and an Android mobile device and a Windows laptop should be able to transfer information via Bluetooth. Enhanced interoperability means improved communication with fewer compatibility issues, faster data sharing, and secure and reliable support for a greater variety of devices or systems. This paper^37^ reviews existing research on semantic interoperability and reusability in IoT, focusing on RDF, OWL, SPARQL, ontologies, and the semantic sensor network ontology. They^38^ proposed a new architecture referred to as Ontology as a Service. They developed a lightweight ontology for context-aware systems, based on existing models but customized to their needs. Their results show that their system successfully mapped and translated between two different systems: smart home and healthcare. They^39^ proposed a transparent translator that addresses interoperability at two levels. The messaging protocol layer handles communication protocols such as COAP, MQTT, and HTTP, while the syntactic layer converts JSON, XML, and CSV using SSN ontology.
3. **Improved Scalability:** Billions of interconnected devices form autonomous, dynamic social relations, collaborating in real time for services like traffic control, public safety, and home automation. These devices generate data simultaneously, leading to numerous dynamic stream queries and frequent topology changes as they join, leave, or move. Scalability is crucial for managing increasing devices, users, and interactions without compromising performance, security, or reliability. AgileDART^40^ introduces a decentralized edge stream processing engine that significantly improves scalability in dynamic, heterogeneous environments. Its architecture eliminates the single-point bottleneck through distributed operator placement and bandit-based routing, making it suitable for high-throughput, low-latency applications in SIoT ecosystems.
4. **Dynamic Trust Management:** A system that continuously evaluates, updates, and adapts the trustworthiness of entities in real time. It considers their environment, context, and behavior to identify and assess new threats and operational conditions.Secure interactions between different IoT devices that belong to different people are essential in the SIoT world (smart homes, healthcare, smart cities). For instance, devices like a smart fridge or smart lock may need to communicate with devices owned by friends, family, or even unknown users. Trust needs to be continuously evaluated and updated based on behavior, rather than assumed. Devices can join or leave the network, behave suspiciously, update their firmware, or experience security breaches. This necessitates dynamic rather than static trust management, where the system automatically recalculates trust levels and quarantines untrustworthy devices. Interoperability between different trust models is also important, as not all devices calculate trust in the same way—some use reputation, risk scores, or certificates. To address this, they^41^ have built RTrustSim, a simulation of the SIoT environment where the trustworthiness of devices can change, be measured, and inform decisions such as joining, staying, leaving, or being quarantined automatically. They demonstrated this framework through three use cases: Smart home, Preventive Health Monitoring, and Dynamic Device Integration.
5. **Security and Trust:** Security in SIoT defends against technical threats such as unauthorized access, data tampering, spying, and attacks, involving authentication, authorization, confidentiality, integrity, and availability. Trust, on the other hand, pertains to evaluating the reliability and honesty of another device to determine whether to interact and share information. It helps devices establish safe and intelligent social connections. Research^42^ has introduced a novel model known as self-adaptive trust management for SIoT. By integrating MAPE-K and machine learning to manage trust, the trust evaluation task was assigned to fog nodes. Their simulation within an SIoT network effectively detects malicious devices.
6. **Self-Organisation:** Smart objects can autonomously locate, connect, and interact with other devices without human intervention, akin to how individuals establish friendships in real life. These devices form social relationships based on criteria such as shared ownership, common location, and similar interests (e.g., devices that support similar services). Interaction history also plays a role in the formation of these connections.
7. **Context-Aware Service: **Devices are designed to be aware of their surroundings and respond intelligently to various situations, much like humans adjust their behavior based on their perception of their environment. These devices employ sensors that detect, comprehend, and adapt to contextual information such as location, time, environmental conditions (e.g., hot, cold, or noisy), user activity (e.g., sleeping, walking, or driving), and device status (e.g., low battery, active sensor).

#### Critical research challenges in SIoT systems

The Social Internet of Things (SIoT) faces a range of persistent challenges rooted in its scale, heterogeneity, and decentralized nature. These include secure trust management, dynamic mobility, interoperability, data privacy, resource constraints, and complexity in service discovery, all of which impact the resilience, reliability, and usability of SIoT environments^9^.

1. **Trust, Security, and Privacy:** SIoT networks are vulnerable to trust manipulation, identity spoofing, inference attacks, and unauthorized data access. The absence of standardized trust metrics and robust authentication frameworks exacerbates these issues, especially under dynamic mobility.
2. **Scalability and Network Management:** As SIoT networks grow, challenges arise in efficient routing, service discovery, and congestion management. Real-time navigation through dynamic social graphs, particularly under dense device populations, remains complex and costly.
3. **Interoperability and Standardization:** Fragmented communication protocols, vendor-specific social models, and the lack of universal standards hinder seamless integration across heterogeneous platforms and devices.
4. **Relationship Modeling and Management:** Devices must dynamically form, update, and revoke social links. Preventing relationship flooding and distinguishing relationship types (e.g., ownership vs. co-location) remains an open challenge.
5. **Autonomy and Intelligence:** Equipping SIoT devices with intelligent decision-making capabilities is constrained by limited computing power, vulnerability of AI models to adversarial inputs, and the complexity of real-time context awareness.
6. **Data Ownership and Governance:** Managing sensitive, distributed data in multi-owner scenarios poses concerns about ownership rights, synchronization consistency, and access control in decentralized environments.
7. **Legal, Ethical, and Social Issues:** Ensuring user-centric SIoT systems demands mechanisms for informed consent, accountability for device actions, and mitigation of embedded social biases.
8. **Service Discovery and Composition:** Discovering reliable services in dynamic, decentralized graphs is challenged by fake service advertisements, limited trust indicators, and context-aware recommendation bottlenecks.
9. **Integration with Emerging Technologies:** Incorporating blockchain, edge/fog computing, and federated learning into SIoT introduces new challenges ranging from blockchain scalability to energy-efficient trust models at the edge.
10. **Mobility and Dynamicity:** Frequent changes in device locations and interactions complicate service continuity, object tracking, and context adaptation across constantly evolving environments.

## Security in SIoT

### Key security requirements and challenges in SIoT

This subsection addresses **RQ**3, which focuses on the security requirements and challenges associated with SIoT systems. Table 5 provide a detailed mapping of these requirements, the corresponding challenges, and potential solution approaches.

#### Security requirements in SIoT

In the Social Internet of Things (SIoT), primary key security requirements are the essential principles that a secure SIoT system must uphold to safeguard users, data, and device interactions. These requirements serve as foundational elements that form the core security framework for SIoT architecture. Additional requirements, such as trust management and non-repudiation, build upon or enhance these primary pillars.

1. Privacy : To protect personal and sensitive data from being exposed or misused, systems focus on giving users control over their information and ensuring it isn’t collected or shared without permission. Techniques like data minimization and anonymization reduce the amount of identifiable data stored or processed, limiting exposure risks. Differential privacy adds noise to datasets, allowing useful insights while preserving individual privacy. Consent-based data access ensures that users actively allow how and when their data is used, supporting ethical and transparent data practices.
2. Trustworthiness: To ensure that devices, users, and services behave reliably and securely, systems use trust mechanisms that evaluate and predict how entities operate, especially in dynamic or social settings. Trust evaluation models based on reputation scores or behavior analysis—help assess reliability over time, while blockchain-based trust logs provide tamper-resistant records of past actions to support transparency and accountability. Federated learning further enables decentralized behavior prediction by training models across multiple devices without exposing sensitive data, reinforcing trust without sacrificing privacy. The^43^ paper proposes a trust management framework in SIoT that enhances trustworthiness evaluation through the integration of social similarity, feedback, and honesty. It introduces a novel trust propagation technique leveraging social relations and contextual data to disseminate trust effectively and identify untrustworthy nodes. Evaluation using real datasets (Sigcomm and Epinion) demonstrates accurate trust estimation and secure interaction facilitation.
3. Integrity: To prevent unauthorized modification of data, systems use techniques that ensure the information remains accurate and intact during transmission and storage. Hash functions like SHA-256 provide a unique fingerprint of the data, helping detect any changes. Digital signatures verify both the origin and integrity of the content, confirming that it hasn’t been altered. Message authentication codes (MACs) add another layer of protection by allowing verification between trusted parties, ensuring that only data untouched by tampering reaches its destination. The authors^44^ propose and implement a framework for analyzing and exploiting smart home IoT firmware. Using reverse engineering, static analysis, entropy assessment, and emulation tools (e.g., QEMU, Radare2), they identify ten critical network-based vulnerabilities—five scoring CVSS 10.0 and five scoring 9.8. The analysis reveals widespread use of unsafe functions (sprintf, strcpy) and absence of security hardening features (NX, PIE, RELRO, Stack Protection). The study offers best practices to secure firmware, emphasizing authenticated updates, strong passwords, and secure boot mechanisms. In this^45^ study proposes a lightweight and secure FOTA mechanism for IoT devices to counter man-in-the-middle (MITM) attacks using a dual-XOR encryption technique, DEFLATE lossless compression, and multi-channel key transmission. Compared to AES-based methods, the proposed model significantly reduces latency, power consumption, and memory usage while maintaining accuracy and resistance to brute-force attacks in constrained environments.
4. Authentication: To confirm the identity of a user or device, systems rely on various authentication methods that verify an entity is genuinely who it claims to be. Common techniques include passwords or PINs for basic access, digital certificates for cryptographic validation, and biometrics like fingerprints or iris scans that link identity to unique physical traits. Multi-factor authentication (MFA) adds extra layers by combining two or more of these methods, making unauthorized access significantly harder and strengthening overall system security. Reference^46^ proposed a solid identity and access control framework that blends decentralized identifiers (DIDs), soulbound tokens (SBTs), and zero-knowledge proofs (ZKPs). The system protects user privacy by allowing selective sharing of credentials, uses ERC721-based SBTs to bind identity in a way that can’t be transferred, and ensures verification through Ethereum smart contracts. These features are a strong match for SIoT environments, helping prevent identity spoofing, safeguard privacy during social exchanges between IoT devices, and support decentralized, trust-driven service access. The inclusion of audit trails and credential tracking also makes it suitable for regulated and sensitive areas like healthcare, logistics, and smart cities.
5. Authorization: To enforce rules about who can access what and what actions are allowed, systems use access control mechanisms that ensure authenticated users or devices only perform permitted operations on specified resources. This is typically achieved through models like Role-Based Access Control (RBAC), Attribute-Based Access Control (ABAC), or Context-Aware Access Control (CAAC), which define permissions based on roles, attributes, or environmental context. Policy enforcement tools apply these rules across systems, while smart contract-based permissions can automate and secure access decisions in decentralized environments using blockchain technologies. A fog-based adaptive context-aware access control framework (FB-ACAAC) enhances traditional XACML by incorporating dynamic, context-driven policy adjustments at the fog layer. It mitigates threats such as man-in-the-middle, privilege escalation, and masquerade attacks through TLS-encrypted communication, least privilege enforcement, and context-aware access decisions. Performance evaluations demonstrate lower latency and adaptive responsiveness compared to standard XACML approaches^47^.
6. Confidentiality : To prevent unauthorized access to data, systems implement mechanisms that ensure only authorized users or devices can view or read sensitive information. This is achieved through encryption methods like AES and ECC, which secure the data itself; access control strategies that regulate who can interact with the data; and secure communication protocols such as SSL/TLS and DTLS, which protect data in transit across networks. Together, these techniques form a robust defense against data breaches and privacy violations.^48^ proposed a lightweight authentication protocol that ensures confidentiality through session key establishment using hashed credentials and XOR operations, without relying on heavy cryptographic primitives like ECC or AES.
7. Availability: To ensure continuous access to services and data when needed, systems adopt strategies that keep operations running smoothly even in the face of faults or attacks. Redundancy and failover mechanisms provide backup components and automatic switching to maintain uptime during failures. DDoS protection helps defend against traffic overloads caused by malicious attacks, while load balancing distributes incoming requests across multiple servers to prevent bottlenecks and improve system responsiveness. Together, these techniques support high availability and reliable service delivery. A trust and QoS-based service recommendation model for SIoT is proposed, incorporating availability, reliability, and efficiency in service prediction using an RSS-based algorithm and trust-aware community clustering^31^.
8. Scalability: To support growth in users, devices, or data without degrading performance, systems employ scalable solutions that maintain efficiency as demand increases. Distributed architectures like fog, cloud, and edge computing spread processing across different layers to avoid bottlenecks. Lightweight protocols and microservices reduce communication and computation overhead, making it easier to handle more connections and transactions. Modular expansion strategies allow systems to add new components or services incrementally without disrupting existing functionality, ensuring smooth scaling across diverse environments. The authors in^49^ propose FLCoin, a scalable blockchain-enabled federated learning architecture for IoT edge networks. They use a committee-based BFT consensus, elected via the FL process, and a sliding window mechanism to limit consensus scope. Combined with linear communication and block pruning, their design achieves a 90% reduction in communication overhead and 35% lower training time compared to PBFT, ensuring high scalability in large networks.
9. Accountability : To trace actions back to responsible users or devices, systems use techniques that ensure every operation is recorded and accountability is maintained. Logging and auditing tools capture detailed records of activities for review and analysis, while blockchain-based immutable ledgers provide tamper-proof evidence of actions. Digital signatures and identity binding link each action to a verified entity, making it possible to detect misbehavior, investigate incidents, and apply penalties when needed—all supporting transparency and trust in the system. In this work, Ref.^50^ propose a hybrid IoT security framework that integrates deep learning for anomaly detection with blockchain for tamper-proof logging. The system simulates sensor data and diverse attack scenarios using SimPy, and leverages blockchain to ensure that all events—including sensor malfunctions and detected threats—are immutably recorded for auditability. The framework achieves 98% detection accuracy across various network sizes and attack types such as DDoS, MITM, and unauthorized access, thereby supporting accountability through traceable, verifiable, and non-repudiable event logging.
10. Interoperability: To enable different systems, devices, and platforms to work together seamlessly, systems use interoperability techniques that allow data to be exchanged and understood across diverse networks and vendors. Standardized communication protocols like CoAP, MQTT, and HTTP ensure consistent message formats and connectivity. Middleware platforms such as FIWARE and Node-RED act as bridges, integrating heterogeneous devices and services. Additionally, ontology and semantic web approaches provide common vocabularies and reasoning frameworks, helping systems interpret and use shared data meaningfully in distributed environments.

#### Security challenges in SIoT

This subsection addresses the research question: **RQ**10. SIoT introduces unique challenges due to its social, dynamic, and decentralized nature. These challenges highlight the complexity of securing SIoT systems, requiring innovative solutions that balance security, performance, and usability. While the previous section highlighted general research challenges in SIoT, this section narrows the focus to security-specific concerns, outlining the essential requirements and unique threats inherent to securing dynamic and decentralized SIoT environments. As shown in Table 5, we map each requirement to challenges, metrics, and solutions, and indicate lightweight feasibility (✓), partial feasibility ($\triangle$), or heavy/unsuitable (✗) for constrained SIoT nodes.

1. ** Dynamic Trust Relationship:** In SIoT, devices and entities interact dynamically, making trust relationships challenging to manage. Trust levels can change rapidly due to various factors, such as device behavior, user interactions, or context. This complexity requires adaptive trust management systems.
2. **Identity Spoofing and Sybil Attack:** Malicious entities impersonate legitimate devices or users, or create fake identities to manipulate the system. This can lead to unauthorized access, data theft, or disruption of services.
3. ** Scalability of Security Mechanism** As the SIoT network grows, security solutions must scale accordingly. However, heavyweight security mechanisms may not be feasible due to resource constraints, requiring lightweight, efficient solutions.
4. **Heterogeneous Devices** Devices in SIoT have varying capabilities, security levels, and protocols. This heterogeneity creates challenges in implementing uniform security measures, making it essential to develop adaptable security solutions.
5. **Data Privacy** Increased sharing can lead to unintended data exposure, compromising user privacy. This requires robust data protection mechanisms and fine-grained access control.
6. **Collusion Attacks:** Multiple devices collaborate to launch coordinated attacks or deceptions, exploiting trust relationships and potentially causing significant harm.
7. **Decentralized Management** Complexity without a central controller, managing security policies and enforcing them consistently becomes increasingly complex, requiring distributed security management solutions.
8. **Context-Aware Access Control** Access control decisions depend on dynamic attributes like location, time, and trust levels. This requires adaptive access control systems that can respond to changing contexts.
9. **Limited Resources** Resource-constrained devices may not support strong encryption or analytics, making it challenging to implement robust security measures without compromising performance.
10. **Malicious Relationship propagation** Attackers exploit trust-based relationships to spread influence, potentially leading to widespread security breaches. This requires proactive measures to detect and mitigate such threats.

**Table 5:** Security requirements (Authentication–Interoperability) with challenges, metrics, solutions, and feasibility.

Ref.	Requirement	Challenges	Metrics/equations	Solutions	Feasible?
^46^	Authentication	Identity spoofing; Sybil; decentralized ID	FAR, FRR; Accuracy = (Valid $\div$ Total) $\times$ 100%	DID; blockchain-based ID; lightweight crypto tokens	DID = $\triangle$; Blockchain = ✗; Tokens = $\checkmark$
^47^	Authorization	Context-aware access; collusion; malicious propagation	Access Effectiveness = (Unauthorized $\div$ Total) $\times$ 100%; Policy latency (ms)	ABAC, CAAC, RAAC; smart contracts; edge/fog enforcement	ABAC/RAAC = $\checkmark$; Smart contracts = ✗
^48^	Confidentiality	Privacy in sharing; weak encryption	Leakage *I* = MI(User; Leaked); Overhead = Cipher $\div$ Plaintext	E2EE; ECC; AES-CCM; DTLS/CoAP; blockchain access control	ECC/AES/DTLS = $\checkmark$; HE = ✗
^45^	Integrity	Tampering; misuse; malicious behavior	Integrity Rate = (Unmodified $\div$ Total) $\times$ 100%; Detection latency (ms)	MACs; signatures; BC-logging; SHA-256; secure boot/updates	MACs/Hashing = $\checkmark$; Signatures = $\triangle$; Blockchain = ✗
^31^	Availability	DoS/flooding; collusion in dense SIoT	Availability = (Uptime $\div$ Total) $\times$ 100%; MTTF; MTTR; PDR = rx $\div$ tx	Rate limiting; edge filtering; redundancy; AI/ML DoS detection; resilient CoAP	Filtering/Redund = $\checkmark$; AI/ML = $\triangle$; Blockchain = ✗
^43^	Trustworthiness	Dynamic trust; false trust; unreliable reputation	Trust Accuracy = (Correct $\div$ Total) $\times$ 100%; False Trust Rate; Convergence rounds	Reputation models; ML-based anomaly detection; decay functions; consensus validation	Reputation/Decay = $\checkmark$; ML = $\triangle$; BC logs = ✗
^51^	Privacy	Graph inference; contextual leakage; excessive sharing	Privacy loss $(\varepsilon )$ in DP; Inference probability; Minimization ratio	Data minimization; DP; edge/fog processing; HE; blockchain selective disclosure	Min/DP = $\checkmark$; Edge = $\checkmark$; HE = ✗
^49^	Scalability	Heterogeneous devices; growth; resource limits	Latency growth $L(N+\Delta )/L(N)$; Throughput (TPS)	Edge/fog computing; federated learning; blockchain sharding; MQTT/CoAP	MQTT/CoAP/Edge = $\checkmark$; FL/Sharding = $\triangle$
^50^	Accountability	No audit trails; decentralized control; absent monitoring	Auditability = (Logged $\div$ Total) $\times$ 100%; Verification success	Blockchain logging; secure audit trails; TEEs; anomaly logs; policy engines	Trails = $\checkmark$; Blockchain = $\triangle$/✗; TEEs = $\triangle$
^18^	Interoperability	Protocol mismatch; heterogeneous devices; cross-domain access	Success Rate = (Successful $\div$ Attempts) $\times$ 100%; Translation latency (ms)	Middleware; IoT-Lite; translators; blockchain federation; W3C APIs	Middleware/Trans = $\checkmark$; Blockchain = $\triangle$

$\checkmark$ = lightweight; $\triangle$ = partial; ✗ = heavy.

### IoT-based attacks relevant to SIoT

This subsection addresses **RQ**4, focusing on conventional IoT attacks that equally affect SIoT due to shared networking protocols, resource constraints, and communication models. Table 6 presents a structured mapping of key threats, corresponding attacks, and their mitigation strategies. Furthermore, Table 7 extends this analysis by categorizing prevalent SIoT attacks across the communication protocol stack, thereby providing a layer-wise perspective on vulnerabilities and potential countermeasures.

This paper^10^ reviews key IoT application-layer attacks, including spyware, malware, flooding, spoofing, code injection, message forging, brute-force, access control, sniffing, and intersection attacks. These attacks target device software, user credentials, and data transmissions, leading to threats against confidentiality, integrity, availability, authentication, authorization, and privacy. The authors categorize attacks by type (active/passive), affected layer, and security impact, offering a clear taxonomy for understanding IoT vulnerabilities. Reference^9^ provide a detailed survey of attack vectors specific to Social-Internet-of-Things (S-IoT), highlighting threats such as Sybil attacks, self-promotion, ballot stuffing, on-off targeted transmission, bad-mouthing, and opportunistic service attacks. The paper reviews mitigation strategies through trust management frameworks, lightweight cryptographic protocols, and blockchain-enhanced authentication. Security vulnerabilities are also evaluated using fuzz testing, game-theory-based attack trees, and GAN-based intrusion detection in edge-enabled S-IoT contexts.

**Table 6:** Recent research on SIoT security: mapping threats, attacks, and existing solutions.

Studies	Threats	Attack	Solutions
^52^	Identity spoofing	DNS/ARP spoofing for redirection or MitM	ML-based IDS using Random Forest, XGBoost, and SMOTE
^53^	Data integrity and Confidentiality	MitM, ARP spoofing/flooding	CNN-based IDPS with SDN Ryu; blocks ports, clears ARP entries
^54^	Trust exploitation	Malicious trusted relationships	Zero Trust via SDN; cert-based auth, peer policy control
^55^	Privacy inference, trust exploitation	Privilege escalation	Anonymization, traffic obfuscation, MAC randomization
^56^	DDoS attacks	Service degradation via IIoT DDoS	MTDTM with ODENet+LSTM; dynamic traffic control, SDN routing

**Table 7:** Layer-wise mapping of prevalent attacks in SIoT communication protocol stack.

Protocol layer	Common attacks in SIoT environments	Example reference
Physical layer	Jamming, Eavesdropping, Radio interference, Signal manipulation	^57^
Data link/MAC layer	Replay attacks, Collision attacks, Identity spoofing at MAC, Selective forwarding	^58^
Network layer	Sybil attack, Sinkhole attack, Wormhole, Blackhole, Routing table poisoning, Selective packet dropping	^59^
Transport layer	Flooding (SYN/UDP), Session hijacking, DoS/DDoS, TCP reset attacks	^60^
Application layer	False data injection, Malicious code injection, Privacy leakage, Malware/botnet attacks, Unauthorized service access	^6^

1. **Node Capture Attacks: ** It involves an attacker gaining physical access to an SIoT device, extracting sensitive information like keys or credentials. The device may continue to function normally unless reprogrammed. This type of attack is common in remote or unattended SIoT deployments, such as in agriculture or logistics, where devices are more vulnerable to physical tampering. Physical tampering with or stealing of devices. Reference^61^ proposed a lightweight authentication scheme tailored for smart home environments, specifically designed to withstand node capture attacks. Their protocol ensures user anonymity, resists key compromise, and uses randomized temporary identities to prevent adversarial exploitation of captured nodes.
2. **Fake Node Insertion :** Adding unauthorized devices to the network. Imitating legitimate devices to blend in, these malicious nodes appear as trusted participants, exploiting weak identity verification in SIoT systems. This enables trust deception and network infiltration. The paper by^62^ introduces a lightweight authentication mechanism combining hash-chains and Bloom filters to mitigate Sybil attacks, wherein attackers inject fake node identities into RPL-based IoT networks. The scheme ensures only legitimate, pre-registered nodes are authenticated, effectively countering fake node insertion and DIS flooding attacks.
3. **Eavesdropping:** Intercepting raw signals (e.g., RF, IR). Eavesdropping is a threat that can arise at both the physical and network layers. For example^63^ demonstrate a practical attack at the physical layer using IR remotes, and propose a lightweight encryption countermeasure to secure device communications. This paper^57^ proposes an effective and accurate method for detecting active eavesdropping in wireless IoT networks by leveraging a deep learning classifier. Features are extracted directly from wireless pilot signals, allowing the system to enhance physical layer security by enabling real-time detection of eavesdroppers before communication is compromised.
4. **Side-channel attacks:** These attacks exploit physical leakage (e.g., power, timing, electromagnetic, or cache patterns) or fault injection to extract sensitive information from IoT devices. For instance, Correlation Power Analysis (CPA) leverages repeated AES key usage by correlating observed power traces with hypothetical key values to recover secret keys. Kuo et al. ^64^ proposed a dynamic AES key replacement mechanism that combines Moving Target Defense (MTD) with lightweight Diffie–Hellman exchange, demonstrating strong resistance even after 20,000 CPA attempts. In the SIoT context, physical and side-channel attacks at the device and control layers can compromise cryptographic keys, enabling long-term infiltration, identity theft, and replayed trust manipulation. These threats are particularly severe in mobile or resource-constrained devices that cannot rely on heavy cryptographic protections. Mitigation approaches include PUF-enabled blockchain frameworks^20^ that apply physical unclonable functions and role-based verification to resist differential fault analysis (DFA) and cache-based exploits, validated through Py-EVM simulations of mobility-driven IoT scenarios.
5. **DDoS Attacks:** A Distributed Denial-of-Service (DDoS) attack involves multiple compromised devices (like a botnet) coordinating to flood a target with traffic, overwhelming it. Since the traffic comes from many IP addresses, it’s hard to block without affecting legitimate users. This type of attack disrupts communication and makes services inaccessible by flooding devices with requests. For example, thousands of IoT devices might be used to flood a DNS provider, taking down major websites. To address the increasing threat of Distributed DoS attacks in smart home IoT systems, Karmous et al.^65^ proposed the SDN-ML-IoT framework that integrates machine learning with software-defined networking to detect and mitigate various types of DDoS attacks, including SYN floods, CoAP floods, and MQTT broker overloads, achieving 99.99 percentile accuracy.
6. **Denial of Service (DoS) Attack:** A single-source attack involves flooding a server or network with excessive requests from one machine or internet connection, overwhelming it and preventing legitimate access. Since the traffic comes from one source, it’s easier to detect and block. This type of attack can disable services, cause timeouts or inaccessibility, and lead to resource exhaustion, commonly affecting cloud-connected IoT systems and public service endpoints. For instance, a single computer might send thousands of fake login attempts to crash a website. Reference^60^ present a lightweight IDS/IPS mechanism integrated into the firmware of Teltonika GPS IoT devices to detect and mitigate denial of service (DoS) attacks, including TCP session hijacking. By employing packet validation and rate-limiting techniques, the proposed solution ensures real-time protection and preserves telemetry functionality through backup routing, enhancing resilience in intelligent transportation systems.
7. **On-Off Attack:** This attack involves a node behaving well initially to gain trust, then suddenly misbehaving, repeating this cycle to evade detection. The inconsistent behavior makes it hard to identify as malicious, exploiting trust in long-term SIoT interactions. It’s a sneaky way to avoid being flagged as a threat. Authors^66^ IV-based model mitigates on-off attacks by detecting trust value fragmentation—a pattern where nodes frequently switch between trustworthy and untrustworthy behavior. Once detected, the system can act to block, penalize, or distrust these devices.
8. **Wormhole attack:** Wormhole Attack involves two colluding nodes creating a tunnel to rapidly transfer packets, falsely advertising shorter routes and distorting the network topology. This routing manipulation allows attackers to bypass normal paths, evade trust mechanisms, and disrupt network operations, commonly affecting wireless SIoT networks that rely on distance-based routing. In this^67^ paper authors have addresses two types of network-layer security threats: wormhole attacks, where malicious devices create a secret shortcut to manipulate data routing, and blackhole attacks, where a malicious node pretends to be trustworthy and then drops all packets instead of forwarding them. Inorder to mitigate these type of attacks they have proposed a cross-layer defense mechanism using an ehnanced support vector machine based framework that leverages physical, MAC, and network layer interactions to detect and isolate malious nodes forming virtual tunnels, though designed for wireless ad-hoc networks, the models protocol independence and lieghtweight behavior analysis make it well suited of SIoT environemnts vulnerable to similar routing attacks.
9. **Sybill attack:** It is a identity based attack where multiple malicious devices create multiple fake identites called as sybill node to manipulate, disrupt, or control a network. Consider that you are using a ride-sharing app that relies on nearby smart cars to find the best route. A Sybil attacker injects many fake “smart cars” into the network, all controlled by them. The system thinks a road is busy or safe when it’s not causing wrong routing or traffic manipulation. This study^68^ proposes SybilPSIoT, a hybrid decentralized method for prevention and detection, leveraging technologies like smart contracts for secure access control, web of trust for relationship verification, Bayesian inference and structural balance for Sybil detection, and game theory for modeling owner behavior and dynamically adjusting thresholds.
10. ** Man-in-the-Middle (MitM):** Intercepting/modifying communication, In a Man-in-the-Middle (MitM) attack, a hacker intercepts and possibly alters the communication between two parties, making it seem like they’re communicating directly with each other when, in fact, the attacker is secretly in control. In order to mitigate attacks on ZigBee/CoAP-based IoT systems via MQTT, the authors in^69^ proposed two intrusion detection systems (IDS). They successfully demonstrated the effectiveness of these systems by intercepting both Denial of Service (DoS) and Man-in-the-Middle (MitM)/masquerade attacks in a real-world experimental setup. Reference^53^developed a CNN-based Intrusion Detection and Prevention System integrated with Software-Defined Networking to mitigate ARP spoofing and ARP flooding MitM attacks in smart homes, achieving 99.96% detection accuracy and 0.02% FAR across scalable SIoT topologies.
11. **Profile Inference Attack: ** It involves analyzing SIoT interactions and behavior patterns to infer sensitive details about users or devices without directly stealing data. This can lead to privacy leakage and behavioral profiling, commonly affecting smart homes, connected vehicles, and SIoT-based services. Attackers piece together seemingly harmless data to uncover privat attributes. Reference^70^ proposed TrafficDiary, a traffic analysis attack that infers user demographic attributes (e.g., age and career stage) in smart homes by analyzing encrypted traffic metadata. Using a dual-channel neural network, it achieves 98.68 percent event detection and 100 percent profile inference accuracy, revealing significant privacy risks even without decrypting content.
12. **Location tracking:** Location Inference Attack involves analyzing communication patterns, timestamps, or signal strength (RSSI) to determine the physical location of users or devices. This passive attack is hard to detect and can lead to privacy breaches and user profiling, often targeting mobile SIoT devices and vehicular networks. Recent advancements in passive Wi-Fi signal analysis have led to sophisticated location tracking attacks. For instance, RFTrack^71^ enables attackers to infer indoor movement patterns of Wi-Fi devices by analyzing unlabeled RSSI values. It utilizes a reinforcement learning-based approach to reconstruct device trajectories and build fingerprint maps without physical access to the target environment.
13. **Replay Attacks :** Reusing captured data to spoof interactions. Replay attack occurs when an intruder captures a valid transmission and maliciously retransmits it later to trick a system into gathering unauthorized access or performing unintended actions. Consider for an example when someone recording your voice saying a seceret password and then playing it back later to fool a system into thinking its still you, even though you are not there anymore. To prevent replay attacks, researchers^58^ they’ve proposed a lightweight authentication protocol called LCAP for SIoT environments. The protocol assigns each device a unique, time-sensitive token—like a digital ID card that changes over time. This dynamic token is generated using the device’s Physical Unclonable Function (PUF) and a timer, ensuring that even if someone captures the token, it’ll be invalid next time. A Merkle tree serves as a ledger of valid tokens, allowing the system to verify and reject any tokens that don’t match.
14. **Service degradation attack:** Service Degradation Attack involves reducing the quality of service without completely disrupting it. Attackers delay responses, introduce jitter, or manipulate partial data flows, causing the system to slow down or become erratic. This stealthy attack affects industrial SIoT systems and smart infrastructure, compromising performance without being overtly noticeable. In industrial IoT (IIoT) and SIoT environments, service degradation attacks can target the communication and control layers of smart devices. The study by^56^ proposed an adaptive Moving Target Defense (MTD) architecture that mitigates service degradation attacks in IIoT by dynamically filtering traffic, performing service migration, and preserving resource availability under DDoS conditions.
15. **Fake service advertisement:** This an active attack targets the registry/discovery layer, where a malicious node registers a non-existent or malicious service (e.g., a fabricated “air-quality sensor feed”). Legitimate users and devices may unknowingly query such entries, leading to wasted resources, privacy breaches, or cascading trust failures. This undermines both service availability and user confidence in SIoT platforms. Recent studies have also explored the use of machine learning for detecting deceptive behaviors such as fake news, bot profiles, and misleading content in online social systems, which can be adapted to address similar challenges in SIoT, including fake profile creation, bullying, and misinformation spread^4^. Blockchain-enhanced Sensor-as-a-Service (SEaaS) frameworks^22^ provide concrete defenses by enforcing provenance, non-repudiation, and ledger-backed validation of service registrations, with effectiveness demonstrated in smart city case studies involving 200 simulated nodes.
16. **Privilege Escalation: **Gaining higher access levels than authorized personnel. In support of the growing concerns around privacy leakage in smart home environments, Ref.^55^ proposed IoTBeholder, a low-cost snooping attack that can infer users’ habitual behaviors and automation rules by analyzing encrypted Wi-Fi traffic. Their findings reveal that attackers do not need network access or prior knowledge to compromise user privacy, thus exposing a significant threat in smart home IoT ecosystems.
17. **Authentication and Authorization attack: **^72^ proposed an EducationalSIoT platform that provides robust authentication and authorization mechanisms for educational IoT environments. Devices are authenticated using digital certificates, ensuring secure identity verification. For authorization, the platform extends the XACML model by incorporating social attributes such as trustworthiness, contact frequency, and social relationships enabling context-aware access decisions. The access and delegation mechanisms are implemented on a Cosmos SDK-based blockchain, and validated through realistic classroom simulations. The authors in^73^ proposed a decentralized solution in which a user needs to register only once and can use a single identity or credential to access multiple service levels offered by various providers. This is achieved by leveraging blockchain technology, smart contracts, the Hyperledger Fabric SDK, and non-interactive zero-knowledge proofs^74^.
18. **Malicious use of trusted relationship attack:** In SIoT or IIoT, devices establish trusted connections, such as a smart lock trusting a smartphone or a medical sensor trusting a hospital server. However, attackers can exploit these trusted relationships to gain unauthorized access or perform malicious activities. Reference^54^ mitigate the malicious use of trusted relationships by eliminating implicit trust between IIoT nodes through micro-segmentation and Zero Trust principles. Peer-to-peer SDN and mutual certificate-based authentication ensure that only explicitly authorized interactions occur, preventing unauthorized trust exploitation.
19. ** Malicious Code Injection Attack :** Code Injection Attack involves exploiting vulnerabilities in firmware or apps to insert malicious scripts or binaries, allowing attackers to execute harmful instructions on target devices. This can lead to remote code execution, malware infection, and device malfunction, often occurring through over-the-air updates or smart apps in SIoT. The malware can remain dormant or cause immediate damage.^6^ reviewed code injection attacks in wireless IoT systems, highlighting vulnerabilities in protocols like Wi-Fi and Zigbee. They demonstrated real-world attacks using Raspberry Pi and reverse-engineered IoT firmware to detect malicious code. The study applied IMECA to assess attack severity, offering both theoretical insights and practical implementations.
20. **Data Leakage attack:** Exposing private or sensitive user data. This paper presents a practical implementation to detect False Data Injection Attacks (FDIAs) in smart grid networks using various machine learning algorithms. The authors simulate FDIA on power data from a 10kV solar PV system in a lab environment and evaluate six ML models (e.g., decision tree, logistic regression, autoencoder). A hybrid ensemble of decision tree and logistic regression achieves the highest detection performance, with an F1-score of 1 and model accuracy of 0.99, demonstrating the effectiveness of machine learning for securing smart grid infrastructures against data manipulation threats^75^.
21. **Data poisoning attack:** Data Poisoning Attack involves injecting manipulated data into machine learning or trust models, corrupting their decision-making. This causes models to make inaccurate or biased decisions. Common in SIoT systems with AI/ML-based recommendations or decisions, these attacks distort trust and compromise model integrity by inserting malicious data into training datasets. The authors^76^ propose VMGuard, a novel four-layer security framework designed to counter data poisoning attacks in the vehicular Metaverse. The attack scenario involves malicious SIoT devices injecting false or misleading data into the system to degrade service quality and user experience. VMGuard uses a reputation-based incentive mechanism powered by subjective logic to assess and manage the trustworthiness of participating SIoT devices, thereby ensuring secure and reliable data collection and service delivery.
22. **Black hole Attack:** In this attack, a malicious node pretends to be the best route for data, but then secretly drops all incoming packets, disrupting communication. This can cause a Denial of Service (DoS) attack, where data is lost without any feedback. This type of attack is common in IoT/SIoT environments that rely on routing. Reference^77^ proposed a novel algorithm to detect and mitigate Black Hole attacks in IoT networks. The approach relies on node authentication, active monitoring by the sink node, and dynamic routing table updates to isolate malicious nodes. Simulations using NS2 and Simulink showed that the algorithm significantly restores throughput and packet delivery ratio (PDR) close to normal network conditions, achieving a PDR of 98.21%, thus demonstrating its effectiveness against Black Hole attacks.
23. **Ballot Stuffing : **Artificially boost a node’s trust or reputation. Colluding nodes give each other positive feedback regardless of their actual behavior. Gives high trust scores despite misbehavior. It could lead to Reputation inflation, trust system manipulation. Reference^78^ proposed a Multi-Dimensional Trust model (MDT) that effectively mitigates trust-based attacks such as ballot stuffing and bad-mouthing in VANETs. The model dynamically adjusts trust weights using an entropy weight method and filters out anomalous recommendations through the Median Absolute Deviation (MAD) algorithm. This helps prevent malicious nodes from falsely inflating the trust of colluding partners, a typical strategy in ballot stuffing attacks, thereby enhancing the robustness and accuracy of trust assessments across the network.
24. **Zero day attack :** It is a cyberattack that takes advantage of a previously unknown vulnerability in software, hardware, or firmware. Since the vulnerability is unknown to the vendor or developer, they have zero days to patch or fix it before the attack occurs. To tackle the difficulty of detecting zero-day attacks in edge-based SIoT systems, a heuristic intrusion detection system named DQN-HIDS was proposed. It employs a Deep Q-Network (DQN) integrated with an LSTM-based learning module to adaptively improve malicious traffic identification under insufficient training data, demonstrating superior detection performance compared to conventional methods^79^.
25. **Bad-mouthing:** In order to lower trust score of honest nodes, malicious nodes submit unjust negative feedback regardless of actual behavior, trustworthy nodes appear as untrustworthy. Reference^78^ mitigate bad-mouthing by using a median absolute deviation filter to discard anomalous indirect trust values, while^80^ apply evidence theory fusion to reduce the weight of untrustworthy feedback.
26. **Identity Spoofing attack:** Spoofing attacks involve masquerading as a legitimate device or user to deceive systems, gaining unauthorized access or trust. By faking identities like IP or MAC addresses, attackers trick systems into accepting them as trusted. This active attack poses threats like data integrity loss, access control breaches, and trust exploitation, especially in IoT where malicious devices can send false data to manipulate systems. This^52^ research article developed an end-to-end ML-based framework to detect spoofing attacks in IoT environments, providing a unified detection model for both DNS and ARP spoofing.
27. **Snooping attack (Eavesdropping) :** Eavesdropping involves intercepting and gathering sensitive information from someone else’s data or communication without authorization. Attackers often passively listen in, capturing data like passwords or messages without altering it. For instance, an attacker might capture unencrypted Wi-Fi traffic to steal login credentials. This passive attack poses threats like privacy leakage and data theft. The IoTBeholder system, proposed by^55^, is a privacy snooping attack tool designed to infer user behaviors in smart homes by passively sniffing encrypted Wi-Fi traffic (802.11 packets), without any physical access or network credentials.
28. **Selective Forwarding Attack:** Selective Packet Drop Attack involves a compromised node in an SIoT network forwarding some packets while silently dropping others. This partial cooperation makes detection challenging. The attack degrades trust and causes packet loss, commonly affecting sensor-based SIoT communication. Reference^81^ proposed FL-DSFA, a federated learning-based model that detects selective forwarding attacks (SFA) in RPL-based IoT networks. It trains local ML classifiers on RPL traffic features (e.g., DIO, DAO, transmission rates) and aggregates them using Federated Averaging, preserving data privacy. The system achieves 95% accuracy and 97% recall, effectively identifying SFA while minimizing communication overhead.
29. **Sinkhole Attack:** It involves a node falsely advertising itself as the most efficient path, attracting and rerouting traffic through the attacker’s node. This enables traffic interception, manipulation, or denial of service (DoS). Such attacks are common in trust-based SIoT routing protocols, where nodes rely on trustworthiness to make routing decisions. Researchers have proposed a hybrid edge-assisted intrusion detection system (EaHIDS) to detect and mitigate Sinkhole Attacks in 6LoWPAN-based IoT networks. This system, introduced by^59^, utilizes a Gaussian Mixture Hidden Markov Model (HMM) to classify node behavior as Normal, Attacker, or Attacked, based on both host and network-level parameters. By integrating SHAP-based feature selection and a lightweight blacklisting mechanism at the edge, the solution enhances detection accuracy and minimizes false positives, energy consumption, and packet loss. According to the study, the proposed method significantly outperforms existing approaches in terms of precision, recall, F1-score, and network efficiency, as validated through Contiki Cooja simulations and FIT IoT-LAB testbed experiments.
30. **Coordinated / Collusive (Sybil-like Misbehavior)**
  In coordinated or collusive Sybil-like attacks, multiple compromised SIoT nodes cooperate at the social or service discovery layer to artificially boost each other’s reputation. When a new service request is issued, these colluding nodes upvote one another, biasing the trust engine so that malicious actors dominate and legitimate nodes are sidelined. The impact is a distorted trust landscape where attackers gain control over service provisioning. Detection and mitigation approaches include game-theoretic intrusion detection models such as the Strategic Game Model (SGM)^82^ and multi-agent IDS frameworks^83^, which simulate collusive behavior and apply counter-strategies. Typical validation datasets involve simulation traces under random deployments with thousands of iterations.

### Layered categorization in SIoT communication protocols:

In this subsection, we discuss the communication architecture in the Social Internet of Things (SIoT). In SIoT, the communication infrastructure is tailored for resource-constrained devices, dynamic social interactions, and scalability across heterogeneous networks. To meet these requirements, a simplified and layered communication model is often adopted, one that diverges slightly from the traditional 7-layer OSI model. The most practical and widely adopted layers for communication in the SIoT environment are summarized in Table 8. SIoT applications span multiple domains and are built upon these foundational layers.

**Table 8:** SIoT protocol stack layers and their functions.

Layer	Roles in SIoT	Handled By
Application Layer	Manages device services,user interaction, social logic,message formatting	MQTT, CoAP, HTTP, JSON, authentication models
Transport Layer	Ensures message delivery(reliability, order, or speed); provides security	TCP, UDP, TLS, DTLS
Network Layer	Routes data using IP addressing	IPv4, IPv6
Data Link Layer	Device-to-device data transfer, MAC addressing	WiFi, Zigbee, BLE

**Application Layer:** The application layer in SIoT manages device-level services, user interactions, and social relationship management. Protocols like MQTT, CoAP, and HTTP operate here, defining how messages are formatted and exchanged using models like publish/subscribe or request/response, while also supporting authentication and trust. Once a message is generated using one of these protocols, it is transmitted over the network using either TCP (for reliable, ordered delivery) or UDP (for lightweight, fast communication). Finally, the message reaches its intended destination via the network layer, where IP addressing and routing (IPv4/IPv6) ensure proper delivery across the internet or local networks.

- **MQTT (Message Queuing Telemetry Transport):** A lightweight publish/subscribe protocol operating over TCP, suitable for smart homes and wearables where reliable delivery is crucial.
- **CoAP (Constrained Application Protocol):** A RESTful protocol operating over UDP, optimized for constrained, low-power devices that require minimal overhead.
- **HTTP (Hyper Text Transfer Protocol):** A request/response standard web protocol operating over TCP, commonly used for IoT dashboards and cloud services, though less efficient for constrained devices.

**Transport Layer:** The transport layer ensures the delivery of messages either reliably or with low latency, using protocols such as TCP, UDP, TLS, and DTLS. Two main transport protocols are used here:

- **TCP (Transmission Control Protocol):** Provides reliable, connection-oriented, end-to-end communication, supporting features like acknowledgment, retransmission, and message ordering. It is used by protocols such as MQTT and HTTP, which require assured delivery.
- **UDP (User Datagram Protocol):** Fast and connectionless, offering low overhead but no delivery guarantees. It is used by CoAP, where quick delivery is prioritized over reliability, making it suitable for constrained environments.
  To secure communication at this layer:
  - **TLS (Transport Layer Security):** Operates on top of TCP, encrypting protocols like HTTP and MQTT, and providing confidentiality, integrity, and authentication.
  - **DTLS (Datagram TLS):** Operates on top of UDP, encrypting CoAP traffic and offering the same level of security as TLS, but tailored for the unreliable nature of UDP.

**Network Layer:** Manages routing and addressing in large, dynamic Social IoT (SIoT) networks. It utilizes key protocols such as:

- **IPv6:** Provides a vast address space.
- **6LoWPAN:** Compresses IPv6 for low-power links.
- **RPL:** Enables routing in lossy and constrained networks.

These protocols ensure efficient and secure routing across mobile and intermittently connected SIoT nodes.

**Link Layer:** Handles direct device-to-device communication, media access control, and error detection. Technologies at this layer include:

- **BLE:** Optimized for energy savings.
- **Zigbee:** Suitable for mesh-based automation.
- **LoRa:** Ideal for long-range, low-power applications.
- **Wi-Fi:** Supports high-bandwidth cloud access.

### Layer-based existing security protocol in SIoT

This subsection addresses **RQ**4, highlighting how security is implemented across various layers of the SIoT architecture. Each layer incorporates specific security protocols aligned with its roles and inherent vulnerabilities. Table 9 provides a comprehensive mapping of SIoT layers, associated security protocols, their primary security objectives, and example attacks—illustrating how prevalent threats are distributed across the protocol stack.

1. Perception layer: When we collect data from devices like RFID tags, sensors, and actuators, we face some serious security threats. These devices can be compromised through node capture, fake node injection, or even physical tampering. To tackle these issues, we are turning to lightweight cryptography protocols such as PRESENT, HIGHT, or TEA which are designed specifically for devices with limited resources. To prevent manipulated or maliciously crafted service announcements in resource-constrained environments, lightweight cryptography solutions like SIMECK^24^ ensure strong ciphertext randomness and structural unpredictability, thereby resisting false service advertisement attacks. Although the Tiny Encryption Algorithm (TEA) is fast and simple, making it suitable for software implementation, it exhibits weaknesses in randomness. On the other hand, SIMECK is a lightweight cipher, but its security can be compromised with few rounds. Existing reduced-round ciphers, such as SPECK-R, require security enhancements to match the strength of their full-round counterparts. To address these limitations, the SIMECK-T construction has been proposed, which combines the strengths of both SIMECK and TEA. SIMECK-T employs an outer layer of SIMECK rounds and an inner layer of embedded TEA rounds. This “nesting” of TEA within SIMECK enhances randomness, resistance to cryptanalysis, and security without relying on substitution boxes (S-boxes). SIMECK-T achieves these security benefits while remaining lightweight, making it suitable for resource-constrained environments.
  In SIoT environmet where devices are resource constrained, light weight cryptographic protocols like PRESENT are particularly suitable. The work by^84^ demonstrates a high-throughput hardware implementation of the PRESENT cipher optimized for medical IoT applications, highlighting its potential for secure and efficient communication in latency-sensitive and resource-limited SIoT systems.
  Advanced Encryption Standard(AES): Ref.^85^proposed a Relativity Strength Security Framework for SIoT that uses AES-256 encryption with relationship-derived keys to secure device communication. The framework integrates Q-learning for adaptive routing and decision trees for service availability prediction, achieving 88.75% security effectiveness and 97.5% service availability. The novel use of social relationship strength as a basis for encryption makes it context-aware and more resilient to attacks.
  Eliptic Curve Cryptography (ECC) is a type of public key cryptography known for offering strong security with smaller key sizes, making it particularly suitable for lightweight environments such as IoT and SIoT. The work by Yang et al.^86^ demonstrates a hardware implementation of a lightweight two-phase authentication mechanism for Industrial IoT (IIoT) using ECC and trusted tokens to ensure secure communication and data integrity in resource-constrained environments. The proposed scheme offers robust security, effectively withstanding various types of attacks, including replay attacks, eavesdropping, man-in-the-middle attacks, and simulation attacks, while also providing strong mutual authentication and ensuring forward secrecy.
  RFID security protocols protect the communication between RFID tags, readers, and backend servers, ensuring confidentiality, authenticity, integrity, and privacy. Given the limited computational power and storage of low-cost RFID tags, these protocols are designed to be efficient and effective.In scenarios where lightweight batch detection of counterfeit tags is required without revealing or managing individual hash functions, the Group-based Slot Constraint (GSC) scheme^87^ offers a scalable solution by exploiting slot correlation among tags within trusted groups. The authors^88^ proposed an ultra-lightweight RFID authentication protocol (ULRARP+) suitable for low-cost RFID tags, addressing limitations of earlier schemes like HB+, which require multiple rounds, are vulnerable to man-in-the-middle attacks, and do not support mutual authentication or key updates. The proposed protocol uses only minimal operations such as XOR, rotation, and permutation. The authors justify the protocol’s security through both informal analysis (covering 12 types of attacks) and formal verification using GNY logic, and explicitly compare ULRARP+ with existing lightweight RFID protocols such as LRSAS+, LRARP, and LRARP+, demonstrating superior security and lower computational cost.
  Physical Unclonable Functions (PUFs) are like a unique DNA for electronic devices. They leverage tiny imperfections that occur during manufacturing to create an unclonable “digital fingerprint” that’s exclusive to each device. This provides a robust way to identify and authenticate hardware. The authors in^89^ propose a three-factor blockchain-based mutual authentication system that leverages Physical Unclonable Functions (PUFs) for hardware-based identity generation, providing resistance against cloning and impersonation attacks. The system includes both formal and informal security analyses, employs PBFT for consensus, and utilizes smart contracts for transaction validation and member verification. It also compares security features such as anonymity, revocation, confidentiality, and resistance to replay attacks with existing approaches.
2. Network Layer : This layer is responsible for transmission of data between devices and servers. Various threats include Eveasdropping, Sybill attack, wormhole and DDOS.To handle these types of attacks various security protocols are discussed.
  IPSec (Internet Protocol Security): Secure communication at IP layer, to keep communication secure in SDN-controlled networks, the cryptographic keys used in IPsec must be updated periodically a process known as rekeying. While traditional IPsec deployments rely on IKEv2 to manage this process, IKE-less SDN environments delegate rekeying responsibilities to the SDN controller. Reference^90^ addressed this by designing, implementing, and evaluating four distinct rekeying algorithms. All four approaches aim to securely replace expired IPsec Security Associations (SAs) without disrupting ongoing communication. These algorithms differ primarily in two aspects such as the timing of inbound and outbound SA installation, and the mechanism used to remove old SAs—either explicitly or implicitly.
  DTLS (Datagram Transport Layer Security): They proposed a modified^91^ DTLS handshake protocol that replaces traditional X.509 certificates with LightCert4IoT, a compact, self-signed certificate framework verified through the Ethereum blockchain. This approach reduces cryptographic overhead, energy consumption, and handshake delay, making it suitable for resource-constrained IoT devices. By leveraging blockchain-verified lightweight certificates and decentralized device registration, LightCert4IoT mitigates common IoT authentication threats, including rogue certificates, DoS vulnerabilities, and central PKI compromise.
  6LoWPAN Security Extensions: Low-power IoT devices have some challenges such as they run on tiny batteries, have limited memory, and send small packets of data (like 127 bytes). But when they need to talk to the internet, things get tricky. IPv6, the standard internet protocol, has big headers (40 bytes) that don’t fit well with these tiny packets. That’s where 6LoWPAN comes in, it’s a special protocol that helps IPv6 work efficiently over low-power wireless networks, like those used in Zigbee and Thread devices. It makes it possible for these small devices to communicate with the internet without wasting energy or resources. Reference^92^ propose SLGAS, a lightweight group authentication protocol for 6LoWPAN networks with PMIPv6 support. SLGAS uses temporary IDs, alias identities, and aggregated MACs to provide secure mutual authentication for resource-constrained sensor nodes, while protecting against threats like key leakage, replay attacks, and impersonation. RPL Secure Mode: Secure routing in low-power networks, the secure mode of RPL has some security limitations, like relying on static symmetric keys that can be vulnerable to attacks. To address this, ref.^93^ came up with a new solution that uses ECDH (a type of cryptographic technique) to dynamically generate session keys and add extra layers of authentication. This approach makes it harder for attackers to intercept or impersonate devices, while also being efficient in terms of computation and communication resources.
  OAuth 2.0 / OpenID Connect: OAuth 2.0 allows third-party apps to access a user’s resources without sharing their login credentials, granting limited access instead. OpenID Connect builds on OAuth 2.0 by adding a layer of authentication, enabling apps to verify a user’s identity and retrieve basic profile information. Reference^94^ OIDC $^2$ enhances OpenID Connect by introducing short-lived, JSON-based Identity Certification Tokens (ICTs) for secure, end-to-end user authentication. They implemented the protocol by extending existing OIDC servers and evaluated its use in real-world applications such as video conferencing, instant messaging, and email. Their approach effectively addresses security threats like token replay, impersonation, and key compromise through mechanisms like proof-of-possession and ephemeral keys, offering a user-friendly alternative to PGP and S/MIME.
3. The Transport Layer: It is responsible for ensuring reliable data transmission, flow control, error checking, and congestion avoidance between socially connected IoT devices over a network. Unlike traditional internet applications, SIoT nodes are often resource-constrained, mobile, and interact in dynamic social relationships, which places unique demands on this layer.
  TLS/SSL:^95^ propose a TLS 1.3 handshake extension that replaces traditional X.509 certificates with Verifiable Credentials (VCs), enabling Self-Sovereign Identity (SSI) authentication directly at the transport layer. Their approach, fully compliant with RFC-8446 and RFC-7250, minimizes changes to the TLS state machine by leveraging existing extensions and introduces a new ($did_methods$) field for decentralized identifier resolution. Implemented in OpenSSL and tested on IoT hardware using the IOTA Tangle as the DLT, the solution demonstrates competitive handshake latency, hybrid authentication support, and scalable identity management for large-scale IoT deployments.
  DTLS : Recent efforts like LightCert4IoT^91^ propose replacing traditional X.509 certificates with lightweight, blockchain-stored credentials, significantly reducing DTLS handshake time and energy overhead, while supporting decentralized trust—an important advancement for constrained SIoT environments.
4. Middleware/Service Layer: The Middleware/Service Layer handles data processing, service discovery, and trust management to mitigate threats like malicious service ads, privilege escalation, and profile inference. The security protocols employed at this layer are reviewed below.
  XACML (eXtensible Access Control Markup Language): XACML is a standard access control policy language developed by OASIS. It’s primarily XML-based (with adaptations for JSON possible) and supports attribute-based access control (ABAC). This makes it highly flexible and capable of fine-grained control, allowing for precise and detailed access control decisions. Reference^72^ came up with a new access control framework for educational IoT settings, building on the XACML model. Their approach adds a social twist by considering device relationships and trust levels when making authorization decisions. They also introduced a way for delegation and prioritized rules, all secured through blockchain technology. This setup ensures decentralized and tamper-proof access control, effectively protecting against threats like Man-in-the-middle and Replay attacks.
  RBAC/ABAC(Role/Attribute-Based Access Control): Policy enforcement mechanisms, Ref.^96^ proposed a multi-factor authentication and key negotiation scheme for smart factories, combining Role-Based Access Control (RBAC), Elliptic Curve Cryptography (ECC), secret sharing mechanisms, and access control lists (ACLs). The scheme supports mutual authentication, session key agreement, forward secrecy, and user anonymity, and is resistant to replay attacks, masquerading, and smart card/device theft. Formal security analysis using BAN logic confirms the correctness of the authentication and key establishment processes.
  Blockchain-Based Access Control: Immutable logs and smart contract enforcement, although the system^97^ does not implement traditional access control models like RBAC or ABAC, it achieves blockchain-based enforcement through cryptographic authentication (ECDSA), smart contract logic restriction, and immutable logging. Only entities with valid private keys can invoke contract functions, and all actions are traceable on the Ethereum ledger, enabling tamper-proof, auditable enforcement suitable for secure IoT data management.
  Reputation-based Protocols: Trust scores are based on device behavior history. Reference^76^ proposed VMGuard, a four-layer reputation-based security framework that mitigates data poisoning attacks in the vehicular Metaverse. The system uses blockchain to maintain immutable reputation profiles for SIoT devices and enforces trust-based access to semantic data sharing. A subjective logic model evaluates SIoT behavior across interactions, and only trusted devices are allowed to contribute data, achieving effective access enforcement through decentralized trust evaluation.
5. Application Layer: The application layer provides the user interface, data presentation, and application logic, making it vulnerable to threats like spoofing, data leakage, and user impersonation. To mitigate these risks, implementing robust security protocols is essential.
  SSL/TLS: Secure communication channels, Ref.^98^ introduced Threat-TLS, a network tool that detects suspicious TLS connections by analyzing traffic for threat patterns like outdated protocols, weak ciphers, and flawed certificates. Integrated with popular tools, Threat-TLS validates threats using CVE data and actively checks for vulnerabilities, enabling near-real-time detection of compromised TLS configurations.
  JSON Web Token (JWT): Authentication and secure session management. Reference^99^ introduced a secure cloud data storage system utilizing JSON Web Tokens (JWT) for stateless, token-based authentication. This approach, when applied to IoT and Social IoT environments, supports scalable, lightweight, and secure access control mechanisms—offering interoperability with multi-cloud storage, time-based OTP, and TLS channels to ensure confidentiality and authenticity across distributed nodes.
  Two-Factor Authentication (2FA): Enhanced user verification. Reference^100^ introduced a two-factor authentication scheme utilizing smart cards for IoT-enabled Telecare Medical Information Systems (TMIS). Designed with resource-constrained biomedical devices in mind, the scheme leverages Hyperelliptic Curve Cryptography (HECC) to reduce computational and communication costs. It achieves mutual authentication, secure session key establishment, user anonymity, and strong resilience against various attacks such as replay, impersonation, and denial-of-service. The scheme’s security is validated through both informal analysis and the formal Real-Or-Random (ROR) model, while performance comparisons demonstrate its enhanced efficiency and cost-effectiveness over traditional ECC-based approaches.
  Digital Certificates / PKI: End-to-end encryption and device identity validation. Reference^101^ present TinyOCSP, a lightweight certificate revocation protocol designed for constrained IoT devices. By optimizing PKI validation workflows using CBOR/CoAP and Bloom-filter-based CRLs, they enable scalable, energy-efficient digital certificate management in resource-limited environments, facilitating end-to-end PKI deployment in IoT.
6. Social Layer (SIoT-Specific):This layer oversees the social dynamics among IoT devices—such as ownership ties, friendships, and neighbor associations. It faces specific threats like trust manipulation attacks (e.g., ballot stuffing and bad-mouthing) and the creation of fraudulent social links. To ensure reliable interactions, appropriate security protocols or models must be integrated to detect and mitigate these risks.
  Trust Management Models (e.g., TIRec, F-TRM): Infer and update trust scores. Trust Management Models for SIoT include solutions like TIRec and F-TRM. The F-TRM model^34^ dynamically evaluates trust based on device friendliness and transactional feedback. It incorporates privacy-preserving mechanisms using GA-based pseudorandom encryption and Attribute-Based Encryption (ABE), offering robust defense against false recommendations, impersonation, and social trust attacks. Its adaptability and layered cryptographic protections make it a suitable trust model for SIoT security protocols.
  Social Relationship Validation Protocols: Authenticate and validate declared relationships, Trust–SIoT^102^ integrates social relationship validation into its trust framework by modeling object-to-object ties (OOR, POR, CLOR, etc.) as a knowledge graph. Using RotL-based knowledge graph embeddings, the framework quantifies relationship strength (C-DoR), which is then incorporated into a neural network-based trust classifier. This approach serves as an implicit social relationship validation protocol in the trust evaluation process.
  Blockchain with Smart Contracts: Record social interactions immutably SCoTMan, proposed by^103^, is a blockchain-based trust model for Social Internet of Things (SIoT) that leverages smart contracts on Hyperledger Fabric. By combining Bayesian trust evaluation with social similarity-based recommendations, SCoTMan tackles scalability and security issues in resource-constrained IoT environments, ensuring robust trust management and low overhead.
  Game-Theoretic Approaches: Detect and mitigate collusion in trust feedback, building on game theory, Ref.^104^ developed GAZETA, a zero-trust authentication framework for 5G IoT networks that effectively counters lateral movement attacks. By integrating Markov games and Bayesian updates with multi-source evidence, GAZETA enhances cyber resilience and optimizes access control based on dynamic trust scores.
  Interoperability and discovery protocols: Key discovery and interoperability protocols in SIoT include mDNS, DNS-SD, UPnP, and DDS, which enable devices to identify and interact with socially relevant peers. mDNS( multicast DNS) allows devices to resolve names without a central DNS server by using multicast within local networks (e.g., finding smartlight.local), while DNS-SD( DNS based service discovery) works alongside mDNS to advertise device services such as a smart fridge offering temperature monitoring. UPnP (Universal plug and play) facilitates automatic discovery and interaction among devices, commonly used in home automation despite some security concerns. DDS (Data distribution service ) is a real-time, high-performance publish/subscribe middleware with built-in Quality of Service (QoS), widely used in robotics, autonomous systems, and industrial IoT for reliable and scalable data sharing.In SIoT communication, certain OSI layers are typically excluded or abstracted due to the nature of constrained devices and simplified architectures. The session layer is often merged into the application layer, as explicit session control is minimal. The presentation layer is also bypassed, with functions like data formatting and encryption handled directly by application (e.g., JSON) or transport protocols (e.g., TLS). Similarly, the physical layer is implicitly managed by link-layer technologies such as BLE and Zigbee, and is rarely exposed or configured directly in SIoT protocol stacks.^105^ implemented and evaluated a low-cost smart refrigerator system that enables users to interact with the device through a mobile application and voice commands. The system captures fridge contents using a Night Vision camera and performs object detection using a lightweight YOLOv5n model, which was deployed on both Raspberry Pi and Android platforms using TensorFlow Lite. Their work focuses on application-layer functionalities such as remote access, natural language interaction, and cloud-based image retrieval over HTTPS.

**Table 9:** Security mechanisms/protocols and threat mapping with referenced studies.

Exemplar Study	SIoT Layer	Key Security Mechanisms / Protocols	Primary Security Goal	Example Threats
^24^	Perception Layer	AES-CCM, ECC/ECDH, PUF-based keying, PKI, HMAC-SHA-256, secure boot / TrustZone-M	Authentication, data confidentiality, device integrity/identity	Device spoofing, physical tampering, key extraction
^92^	Network Layer	IEEE 802.15.4 security (AES-CCM*), 6LoWPAN, RPL (secure modes), IPsec/ESP (IPv6), Thread, LoRaWAN 1.1 security	Secure data transmission, routing integrity, link-layer confidentiality	Eavesdropping, wormhole, Sybil, link replay
^91^	Transport Layer	TLS 1.3, DTLS 1.3, QUIC/HTTP/3 (where applicable)	Encrypted transport, session security, forward secrecy	Replay, man-in-the-middle, downgrade
^94^	Middleware / Service Layer	OAuth 2.0, OpenID Connect, ACE-OAuth profiles, UMA 2.0, XACML (ABAC), Macaroons, Zero-knowledge proofs	Access control, delegated authorization, privacy-preserving authorization	Privilege escalation, token theft/misuse, profile inference
^101^	Application Layer	OSCORE+COSE/CWT, CoAP+DTLS, MQTT/MQTT-SN over TLS, LwM2M Security (DTLS/OSCORE), OPC UA Security, DDS Security, XMPP+TLS, JWT	End-to-end/object security for application data, secure messaging, session integrity	Spoofed messages, data leakage, injection/replay
^103^	Social Layer	Blockchain smart contracts, Verifiable Credentials / DIDs, reputation systems (Beta, EigenTrust, Subjective Logic), Sybil-resistant graph methods (e.g., SybilRank/Guard), game-theoretic trust models	Trust management, reputation validation, social relationship integrity, Sybil resistance	Bad-mouthing, ballot-stuffing, fake relationship creation, collusion

OSCORE = Object Security for Constrained REST; COSE = CBOR Object Signing and Encryption; CWT = CBOR Web Token. Stack items like 6LoWPAN, IEEE 802.15.4, Thread, and LoRaWAN are listed as mechanisms where their security modes are employed.

## Emerging trends and applications of SIoT

**Figure 12:**
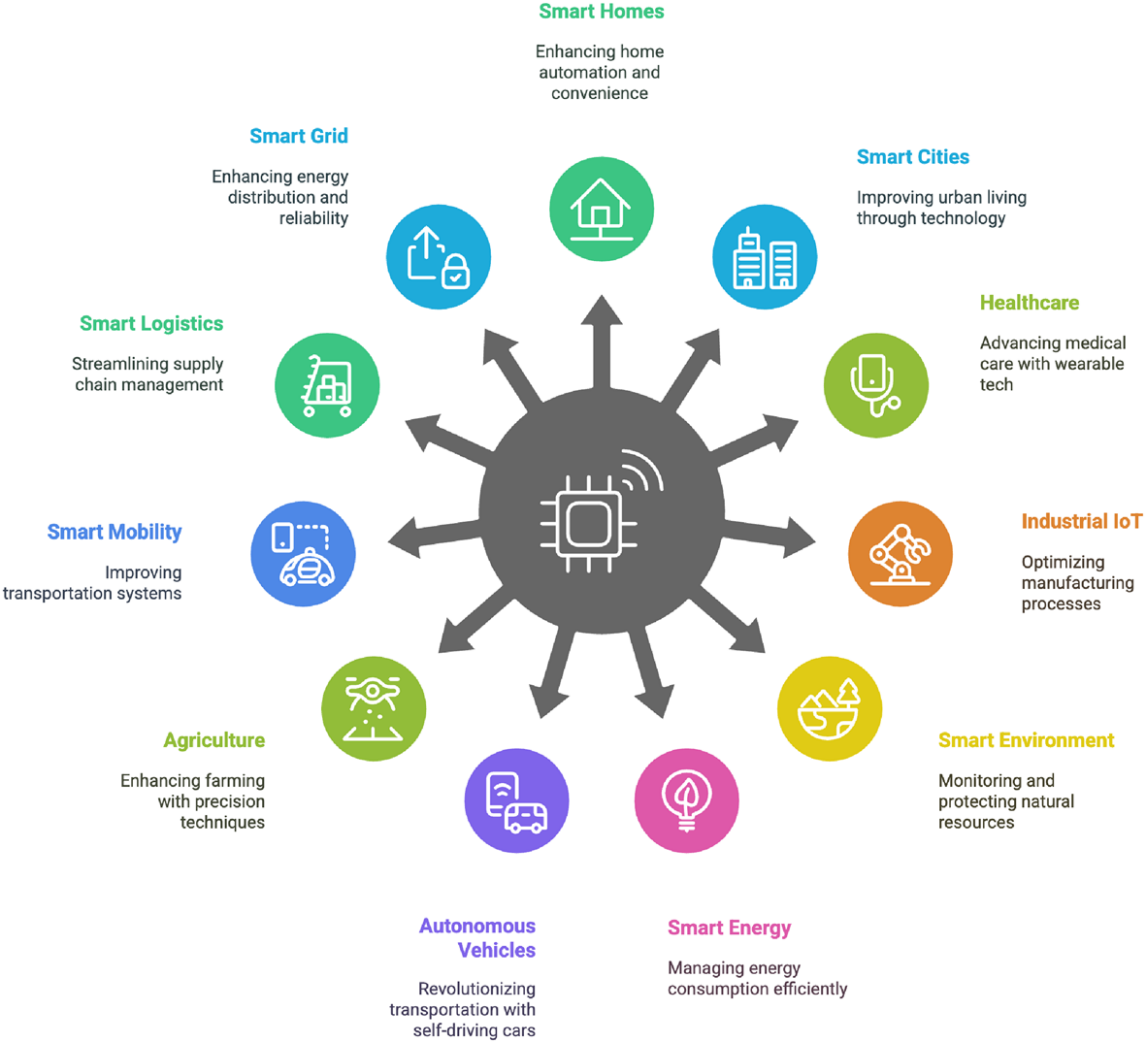
Applications of social internet of things (SIoT).

This section addresses **RQ**5, focusing on emerging trends in SIoT research and their reflection in practical, real-world applications. As shown in Table 10 and Fig. 12, various SIoT applications are categorized and illustrated, including domains such as smart healthcare, transportation, logistics, and industrial IoT.

### Emerging trends in SIoT

**1. AI-Driven SIoT**

The work in^106^ implemented an AI-driven digital city platform leveraging various technologies such as IoT, AI, cloud computing, big data, and cybersecurity to create an intelligent and data-driven urban management system. Specifically, focusing on Indonesian cities to enable them to regulate the data-based governance system with real-world implementation in Semarang city.

**2. Blockchain for SIoT**

The research proposed in^107^ develops a simulation-based blockchain SCM to improve trackability, security, and efficiency in shipment tracking; they have implemented a digital ledger where each item has been assigned a unique ID, ensuring immutability through the hash pointer connecting transaction blocks. In addition, they have implemented IoT base real-time tracking using navigation and communication sensors to monitor shipment and detect lost items. They integrate machine learning technique for backorder prediction using customer data from Kaggle to train models like SVM, KNN, Random Forest and AdaBoost, the performance evaluation shows that random forest shows surpass over other models. Reference^108^ proposed a decentralized Ethereum-based payment framework tailored for low-connectivity environments. The model integrates auxiliary nodes, smart contracts, and incentive-driven auditors to enhance trust. Empirical results showed a 79% reduction in block time, 28% increase in throughput, 30% lower energy consumption, 68% shorter confirmation time, 63% reduced execution time, 46% higher block production rate, and 82% reduced network variability, offering a resilient and secure architecture applicable to SIoT contexts.

**3. Edge and Fog Computing**

This paper^109^ proposes a double auction-based incentive mechanism called Truthful Auction for Fog Systems (TAFS) to enhance IoT applications in offloading computing networks. TAFS incentivizes fog nodes to share idle resources, maximizing resource utilization while maintaining fairness and truthfulness. It ensures economic properties such as truthfulness, individual rationality, and budget balance. The paper also presents a heuristic algorithm to efficiently allocate resources, minimizing latency. Simulations demonstrate that TAFS improves system efficiency, user experience, and fairness compared to previous methods.

**4. Integration of 5G and SIoT**

The analysis presented in^110^ demonstrates the integration of blockchain and the Social Internet of Things (SIoT) by proposing a hybrid trust management system. This system ensures secure, decentralized and reliable communication between autonomous devices within 5G networks, which is optimized for environments with constrained devices and can function effectively even in partial or no coverage scenarios using local trust mechanisms. In addition, it provides security against Sybil attacks and malicious tampering through the application of blockchain technology.

**6. Digital twins in SIoT:**

They^111^ have introduced a new innovative approach, a software-based security layer called ’CommandFence’, a framework based on the concept of digit twin for smart home systems compared to the existing access control mechanism, which significantly improves security without requiring hardware changes; that prevents risky states from being encountered when an application command interacts with human activities and environmental variations. In^112^, they have designed and developed a Digital Twin Authoring Tool (DTAT) that creates real-time digital replicas of physical objects to facilitate smart cities in optimizing transportation and urban planning using 3D modeling, VR and simulation techniques.

### SIoT applications

The applications of SIoT span across various industries, enabling connected devices to communicate, coordinate, and make autonomous decisions while integrating social interactions. SIoT is expanding across various industries such as industrial manufacturing, retail and e-commerce, automotive and transportation, smart homes, etc. As shown in Fig. 12, various SIoT domains exist.

1. **Smart Homes and Personal Assistants ** According to this analysis^111^, they have examined the access control mechanism in voice-controlled systems within multiuser environments, specifically focusing on Amazon Alexa. They have identified two critical vulnerabilities, such as the use of simple commands and targeted commands. They have revealed security flaws in the existing voice control mechanism by highlighting its risk and providing recommendations to enhance security while also encouraging service providers to improve access control. They^113^ proposed 2FIDS, a fog-based federated learning intrusion detection system for smart homes. It allows IoT devices to collaboratively train a deep learning model (LSTM) without sharing raw data, preserving privacy. The system operates at the fog layer using secure communication (TLS over gRPC), ensures trusted client registration, and applies model compression to reduce latency and overhead. It is tested on three real IoT datasets (BoT-IoT, TON-IoT, MQTTset), achieving high detection accuracy (>96
2. **Smart Cities**
  This study demonstrates^114^ by the integration of RPA, LCDP, ISSP to optimize smart city automation. Robotic Process Automation (RPA), which is a software technology that automates repetitive, rule-based digital tasks (more like a bot that mimics human actions on a computer), acts as a sensing tool alongside technologies like IoT devices, sensors, and APIs, it works within the ISSP. It is used for automating rule-based digital tasks (e.g., web scraping, data entry). RPA mimics human interaction with web portals and databases to collect and integrate data (e.g., extracting flood report from government websites). Low-Code Development Platforms (LCDP) and the Integrated Smart System Platform (ISSP) to optimize smart city automation. LCDP enables non-programmers to design automation workflow using a drag and drop interface. ISSP is the core framework for automation by integrating different data sources, IoT devices, AI while RPA can help extract and process unstructured data that traditional IoT cannot handle.They^115^ proposed an IoT-enabled LSTM-based model for predicting pressure anomalies in urban water supply systems. Using real-time sensor data and seasonal time features (month, hour, day type), their optimized LSTM model achieved a MAPE of 4.79%, enabling early accident detection and faster emergency response. The study used a real-world prototype in Gomel, Belarus, and highlighted the benefits of using deep learning for smart city infrastructure monitoring, though it was limited by dataset duration and lack of standard simulation tools. In this paper they^116^ proposed a graph-based scalability enhancement scheme for Ethereum blockchains, introducing a Proof-of-Validation (PoV) consensus with auditor nodes, hash-binding, and efficient replication/retrieval to reduce storage overhead. The scheme was implemented in Python and simulated with 2000 nodes, 10–50 transactions per block, and up to 50,000 blocks. Results show that nodes require only 15 MB storage overhead (vs. $\sim$8 GB in conventional Ethereum), new node configuration takes $\sim$370 ms ($\approx$ 320$\times$ faster than 4–5 h in baseline Ethereum), and retrieval/validation achieves significantly higher throughput, >30% lower latency, and reduced processing time compared to EBC, ESSE, and ESM models. These improvements make the design well-suited for IoT and smart city SIoT deployments where scalability and trust are critical.
3. **Healthcare and Wearable Technology **
  In their study^117^ they provide a valuable insight into comparative performance analysis of popular wearable devices such as Fitbit Sense, Empatica E4, and GSR3 plus to monitor electrodermal and cardiac activities.^118^ provides detailed information on current technologies in AI and available wearable devices dedicated to sexual health. This study^119^, indicates that by incorporating technologies such as machine learning, the social Internet of Things, and cloud architecture, one could improve the provision of healthcare service in cities by ensuring accurate and time-sensitive data distribution, leading to efficient healthcare management. It provides a detailed review while emphasizing Internet of medical Things, its emerging technologies, also provides analysis of for disease prediction and remote monitoring innovative approaches for integration of ML and AI in IoMT. Privacy and security measures by providing advanced cryptographic solutions and incorporating blockchain technology for data protection. In this work^120^ authors proposes ML-RASPF, a machine learning-based framework for real-time and rate-adaptive IoT service provisioning in smart healthcare environments. It utilizes a mist-edge-cloud architecture and integrates LSTM for traffic prediction, GBDT for delay estimation, and Deep Q-Network (DQN) reinforcement learning for adaptive control. The framework jointly optimizes latency and service delivery rate, outperforming four baselines in simulation. It achieves up to 20% lower latency, 18% higher throughput, and 19% reduced energy consumption, making it suitable for dynamic, QoS-critical healthcare applications.
4. **Industrial IoT (IIoT) and smart Manufacturing:**Their paper^121^ introduced “federated learning” for privacy-aware decentralized training. This method optimizes communication and power resources while ensuring high data transmission for device-to-device communication and cellular users in 6G IIoT digital twin edge networks. Improve network throughput and reduce inference.
5. **Smart Environment ** The authors^122^ developed a low-cost, real-time computer vision system for a small humanoid robot using an ESP32-CAM and a lightweight tiny-YOLO model, enabling accurate object detection and decision-making in crowded environments with minimal hardware requirements.
6. **Smart Energy **
  The paper^123^devises an integrated MILP-based solution to optimize the deployment of Smart Mobile Power Banks (SMPBs) for on-demand electric vehicle charging and vehicle-to-grid (V2G) support, leveraging a bi-level optimization framework and real-world data for simulation-based evaluation. A blockchain-enhanced AI framework has been proposed to improve power consumption prediction in smart grids. The system incorporates Z-Score normalization and spatial-temporal correlation (STC) for data preprocessing and feature extraction, while forecasting is handled by an LSTM-RNN optimized using the Improved Sparrow Search Algorithm (ISSA). To ensure secure and decentralized data exchange, it integrates a blockchain-based authentication and authorization (DAA) mechanism. The BSET-AVVO protocol enables low-latency communication and adaptive Volt-VAR optimization for real-time demand response. According to^124^, this model demonstrates superior performance over existing methods in terms of MSE, energy efficiency, latency, throughput, and response time. Reference^125^ developed a decentralized application using Ethereum smart contracts to manage power balancing in renewable energy grids with prosumers. Their prototype, tested on the Volta testnet, ensures non-repudiable command dissemination, trustless modulation execution, and prosumer accountability. The system supports up to  290 distributed energy resources (DERs) before hitting performance limits due to transaction delays, gas costs, and network congestion. The study emphasizes the suitability of blockchain for secure, scalable energy coordination, but also highlights the constraints of public Ethereum networks under time-sensitive loads.
7. **Autonomous Vehicles and Smart Transportation: **
  This study^126^ provides an in-depth analysis of the Tesla Model 3 standard range with lithium iron phosphate cells. Provides open-source experimental data on powertrain efficiency, range, and operation strategies. This could be valuable information for improving electric vehicle technology, mitigating environmental impact, and supporting the transition to clean energy.
8. ** Agriculture and Precision Farming:** The research presented in^127^ provides us with comprehensive bibliometric analysis to explore global trends, and emerging research gaps in IoT-driven soil less farming, it highlights various aspects of opportunities and challenges in using IoT-enabled smart precision farming in soil less agriculture, analyzed technological advances for real-time monitoring automation and AI-driven decision making for improved crop production, and identified obstacles (such as energy consumption, technological dependency, and the need for specialized expertise), also they have examined the leading nations and institutions at the cutting edge of research in this field. They emphasize that interdisciplinary research, policy support, and technological advancement will be essential for developing global smart farming solutions. They^128^ have provided a better solution for a smart farming using machine learning techniques by developing a Smart farming System. The workflow of this system is done in five stages which includes data acquisition (Rice Seedling and WeedNet datasets), feature extraction (MobileNet architecture), classification (SVM to distinguish between rice and land and crops and weed), segmentation (K-means clustering) and performance evaluation (accuracy, precision, recall, and F1 score). They successfully demonstrated from their result the higher efficiency in classification, weed detection, and precision agriculture. Reference^129^ proposed an AI-driven agricultural intelligence model combining IoT sensors, cloud analytics, edge computing, and blockchain to enable precision farming, pest control, and supply chain transparency, showing improvements like 30% water savings and 20% increase in crop quality.
9. **Smart mobility **
  To address the challenge that traffic outlier detection struggles with real-time performance and fails to properly handle the randomness and uncertainty in traffic flow data, the authors^130^ proposed a solution by combining Stochastic Differential Equations (SDEs) with Gaussian Process Regression (GPR) to create a framework capable of modeling both deterministic trends and random fluctuations in traffic data. They leveraged Bayesian inference, employed the Whale Optimization Algorithm (WOA) for hyperparameter tuning, and used a bootstrapped thresholding method to control the false positive rate. The model was evaluated using real-world traffic data from California’s PeMS dataset and compared with baseline methods such as polynomial regression and standalone GPR. The proposed method achieved high performance with an AUC of 0.938, FPR of 1.95%, and MSE of 0.0013, outperforming the baseline approaches.
10. **Smart logistics**
  The authors^131^developed a reinforcement learning-based framework to dynamically plan and control the paths of multiple automated guided vehicles (AGVs) in smart warehouses. Their model integrates real-time data from IoT and CPS systems to optimize AGV routing, task assignment, and battery management. They validated their approach through simulation, showing improved order completion rates and reduced travel distances compared to traditional optimization methods.
11. **Smart retail and supply chains:** In^132^, they have conducted a game theory based analysis to compare different financing strategies with and without blockchain. They have analyzed the approach of mathematical modeling of financing by considering two scenarios such as non-cooperative vs. cooperative, with and without blockchain. They have proposed a risk sharing mechanism to improve finance coordination in the supply chain, with the numerical analysis they were able to realize that the impact of blockchain adoption would have potential benefits and provide decision making insight for financial managers and supply chain participants.
12. ** Smart Grid **: The authors^133^ developed a novel optimization algorithm to assess and manage smart grid operations under emergency conditions. Their method integrates a Mixed-Integer Linear Programming (MILP) optimizer with Artificial Neural Networks (ANNs) to forecast renewable energy (PV and wind) production. The goal is to maximize grid autonomy while minimizing $\hbox {CO}_{2}$ emissions and energy curtailments. The system dynamically adapts to the disconnection of key components (e.g., PV, BESS, diesel generator) and evaluates the impact of 15 emergency scenarios on autonomy, sustainability, and post-emergency recovery. The solution is tested on a real smart grid model in Spain using PowerFactory, forming part of the EU Horizon 2020 TIGON project.

**Table 10:** Review of application-based studies in SIoT.

Ref. No.	Domain	Use Case	Technique	Proposed Method	Key Features	Outcome / Benefit	Limitation
^120^	Smart Healthcare IoT	Smart hospital provisioning (ECG, kiosks)	LSTM (Traffic), GBDT (Delay), DQN (Rate), ML-RASPF	Mobility-aware provisioning	Latency and SDR co-optimization, predictive AI	$\downarrow$20% latency, $\uparrow$18% SDR, $\downarrow$19% energy	Simulation-only; no adversarial evaluation
^115^	Smart City	Urban water pressure anomaly prediction	IoT, LSTM, MQTT	Optimized LSTM with seasonal time features	Real-time forecasting, median filtering, rolling window	4.79% MAPE; faster emergency response	Limited dataset; no external simulation tools
^129^	Smart Agriculture	Precision farming with supply chain transparency	AI, IoT, Edge/Cloud, Blockchain, Drones	AI-based SoS framework (Agricultural Intelligence Model)	Sensor monitoring, predictive analytics, traceability, precision irrigation	30% water savings, 20% yield quality improvement, 40% pesticide reduction	Connectivity issues; high adoption cost; limited expertise
^131^	Smart Logistics / Smart Factory	Dynamic path planning for AGVs under real-time warehouse conditions	Reinforcement Learning (Categorical DQN), CPS	DSSIF framework integrating CPS + RL for AGV routing, coordination	Real-time data integration, multi-AGV coordination, battery-aware dispatching	$\uparrow$Order completion ( 20%), $\downarrow$travel distance, better adaptability	Uneven workload among AGVs; local optima; simulation-only
^133^	Smart Grid	Emergency-aware energy optimization	ANN, MILP, PowerFactory	EMS optimizer + ANN-based RES forecast	Autonomy maximization, CO_2_ impact analysis	Up to 46% autonomy loss recovery; 25% RES curtailment	Single-grid focus; lacks real-world deployment
^122^	Humanoid Robotics	Real-time object detection and interaction for a small humanoid robot	Tiny-YOLO, ESP32-CAM, Python, HC-SR04	Lightweight vision system with decentralized control	Real-time streaming, object detection, distance estimation, modular setup	High accuracy (up to 0.99), low latency (0.028s), low-cost design	Limited range ($\le$200 cm); relies on external PC for decisions
^113^	Smart Home	Intrusion detection in fog-assisted IoT networks	Federated Learning, LSTM, Fog Computing	2FIDS: Fog-Federated Intrusion Detection System	Decentralized, privacy-preserving, secure gRPC+TLS, scalable to 15 nodes	>96% accuracy (BoT-IoT, TON-IoT); 86% (MQTTset); reduced latency	Lower accuracy on rare attacks; non-IID impact not analyzed
^123^	Smart Energy	On-demand EV charging and energy delivery via SMPBs	Bi-level Optimization, MILP	Coordinated routing and scheduling of SMPBs for EV and V2G	Integrates EV demand, traffic, and grid signals	$\uparrow$Charging efficiency, $\uparrow$grid reliability, $\downarrow$unmet demand	Relies on accurate demand forecasts; real-time scalability concerns
^130^	Smart Mobility / Traffic	Outlier detection in traffic flow streams	SDE, GPR, Whale Optimization	Bayesian SDE-GPR with sliding window + likelihood test	Captures drift/diffusion; bootstrapped thresholding; adaptive	$\uparrow$AUC to 0.938; $\downarrow$FPR to 1.95%; 13.3% more accurate than baseline	Diffusion instability; high-frequency data complexity

## Technology

This section addresses **RQ**6, focusing on the integration of three key technologies—Blockchain, Edge/Fog/Cloud Computing, and AI/ML—within IoT and SIoT environments. For Blockchain, we discuss core features such as smart contracts, immutable ledgers, consensus mechanisms, blockchain types, and typical blockchain workflows. We then introduce the role of edge, cloud, and fog layers in enhancing SIoT functionality. Additionally, we explore the capabilities of AI/ML, including common ML techniques used in SIoT. For each technology, recent studies (2023–2025) are reviewed to demonstrate their benefits, limitations, and applications in enabling secure, intelligent, and decentralized SIoT operations.

### Blockchain technology

Blockchain in SIoT serves as a decentralized ledger that records interactions, enables secure transaction logging, and supports trust frameworks through smart contracts and consensus mechanisms. It enforces a tamper proof permanent record, enables decentralized trust and identity management, and allows secure service access with detailed interaction logging. This eliminates reliance on central authorities, enhances transparency and data integrity, and helps prevent trust manipulation and data forgery. However, challenges include scalability issues such as latency and energy use on public chains, increased storage and resource demands on IoT devices, and the complexity of integrating smart contracts with flexible trust models. Reference^134^ classify blockchain systems into public and private types, where public blockchains like Bitcoin and Ethereum enable unrestricted participation, while private blockchains such as MultiChain limit access to verified entities. They also examine key consensus mechanisms—including Proof of Work (PoW), Proof of Stake (PoS), Practical Byzantine Fault Tolerance (PBFT), and Delegated Proof of Stake (DPoS)—each offering distinct trade-offs in energy efficiency, scalability, and trustworthiness. The study introduces smart contracts as autonomous, self-executing code blocks embedded within blockchain networks, allowing agreements to unfold without third-party intervention. Additionally, it emphasizes the immutable, tamper-proof, and auditable nature of blockchain and IOTA technologies, both of which reinforce data integrity and trust in decentralized environments.

**A. Smart Contract:** A smart contract is like a digital agreement that automatically executes when certain conditions are met, with rules and actions written into code. It’s similar to a vending machine where you put in money, select a snack, and the machine dispenses it, automating processes and ensuring all parties follow the rules without intermediaries.

**B. Immutable ledger:** An immutable ledger is a record book that can’t be altered or deleted once something is written in it. It’s like a permanent, transparent diary that keeps a history of all transactions or events, ensuring their accuracy and trustworthiness. Think of it like a notarized document, but digital, where every entry is time-stamped and linked to previous entries, making it tamper-proof and reliable.

**C. Consensus mechanism:**

The consensus in blockchain is a protocol that ensures that all nodes agree on the ledger’s state, preventing issues like double-spending and maintaining integrity. It’s crucial for trust and security, especially in dynamic environments with resource-constrained devices, where selecting the right consensus algorithm impacts scalability, security, and energy efficiency. Types of Consensus Mechanisms as follows :

1. Proof of Work (PoW): Used in Bitcoin, but high computational cost makes it unsuitable for SIoT due to energy and time constraints.
2. Proof of Stake (PoS): Validators are chosen based on their stake in the network, more energy-efficient than PoW, but may require reputation-aware modifications for SIoT contexts.
3. Practical Byzantine Fault Tolerance (PBFT): Tolerates malicious or faulty nodes (up to 1/3), often used in permissioned blockchains, suitable for closed SIoT networks like smart cities or vehicular networks with known node identities.
4. Delegated Proof of Stake (DPoS): Users vote for a small group of validators, offering high throughput and faster consensus, useful for lightweight or semi-centralized SIoT.
5. Proof of Authority (PoA): Consensus is reached by a few trusted nodes, suitable for private SIoT deployments like industrial IoT with known stakeholders.
6. Federated Consensus: Used in systems like Ripple or Stellar, devices agree via trusted subsets or quorum slices, ideal for trust-based SIoT environments.

**D: Types of Blockchain** Different types of blockchain such as public, private, consortium, and hybrid are used based on the specific requirements and trust model of the application, such as data privacy, access control, decentralization level, and scalability. As summarized in Table 11, different blockchain architectures offer varying levels of transparency, scalability, and suitability for SIoT environments. Each type is briefly described below.

1. Public Blockchain:A public blockchain is open to everyone, fully decentralized, and allows anyone to join, view, and participate in the network. Examples include Bitcoin and Ethereum. This type of blockchain is suitable for applications like Open SIoT systems, global data sharing, and cryptocurrencies, where transparency and accessibility are crucial.
2. Private Blockchain: A private blockchain is restricted to a single organization or group, with controlled access and permissions. Examples include Hyperledger Fabric. This type of blockchain is ideal for internal SIoT deployments and secure enterprise applications, where data privacy and access control are essential.
3. Consortium (Federated) Blockchain: A consortium blockchain is controlled by a group of pre-selected organizations or nodes, offering a semi-decentralized structure. Examples include R3 Corda and Quorum. This type of blockchain is well-suited for collaborative SIoT platforms, such as smart cities or joint industry projects, where multiple stakeholders need to work together while maintaining some level of control.
4. Hybrid Blockchain: A hybrid blockchain blends public and private blockchain features, allowing for both transparent and restricted data access. Examples include Dragonchain and IBM Food Trust. This model is ideal for SIoT applications requiring a balance of transparency and privacy, such as supply chain management with IoT devices.

**Table 11:** Blockchain types and their relevance to SIoT applications.

Blockchain type	Examples	When to choose
Public	Ethereum, Polygon	Open networks where high transparency and decentralization are needed
Consortium	Hyperledger Fabric	Enterprise or research environments with multiple known stakeholders
Permissioned	Hyperledger Indy, Corda	Applications requiring strong identity management and privacy
Lightweight (IoT)	IOTA, Algorand, Nano	Ideal for low-power SIoT devices; supports DAG or fast consensus mechanisms

**E: Typical Blockchain Workflow** The typical blockchain workflow in SIoT begins with a transaction initiation phase, where a user or IoT device, such as a smart sensor, actuator, or camera, generates a transaction. This transaction could involve sending sensor readings, requesting access to a service, or initiating device-to-device communication. Each transaction includes essential information such as the initiator’s identity, the intended action, and a digital signature that authenticates the request. Once created, the transaction is broadcast to all nodes in the peer-to-peer (P2P) blockchain network. This ensures that every participating device or node becomes aware of the request, promoting transparency and decentralized handling. In this phase, all nodes in the network receive the transaction and prepare to validate it without relying on a centralized authority. The next step is validation, which is handled by the blockchain’s consensus mechanism. This is a critical phase where the network agrees on the legitimacy of the transaction. Different types of blockchains use different consensus algorithms. For instance, Proof of Work (PoW) relies on solving complex mathematical puzzles, while Proof of Stake (PoS) selects validators based on their economic stake in the network. In permissioned or consortium blockchains often used in SIoT, protocols like Practical Byzantine Fault Tolerance (PBFT) are preferred for their efficiency and fault tolerance. Through consensus, the transaction is either approved or rejected by the network.

**Figure 13:**
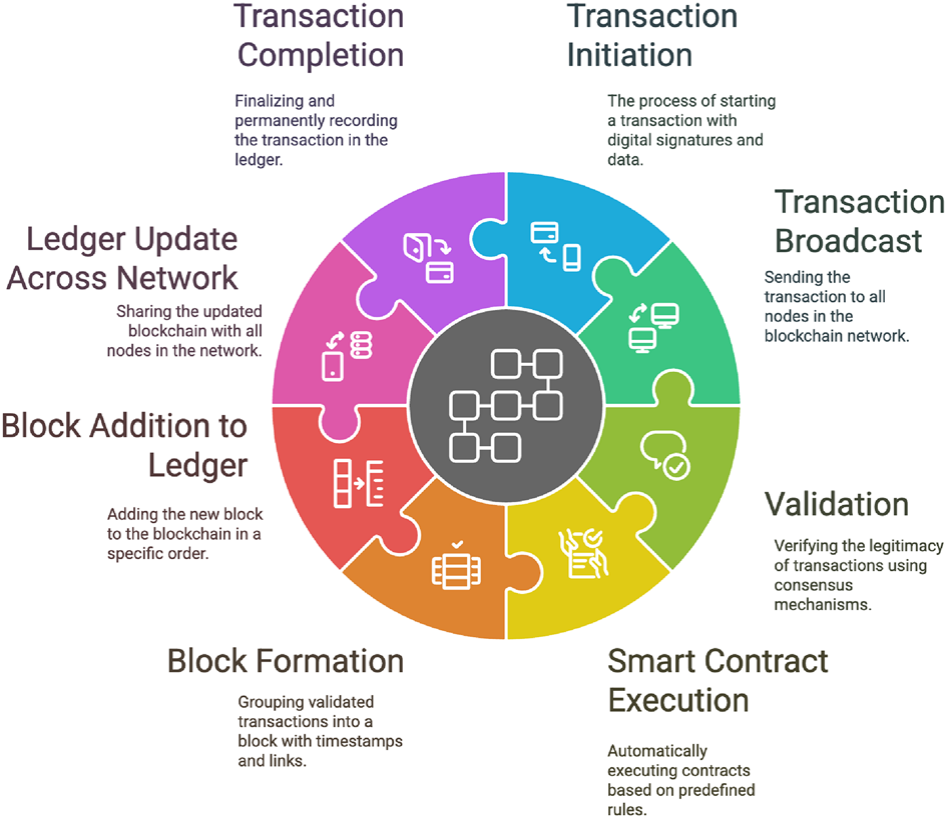
Blockchain workflow cycle.

If the transaction involves predefined logic or conditions, it may trigger a smart contract. Smart contracts are self-executing code segments embedded in the blockchain that automatically perform actions when certain criteria are met. For example, if a temperature sensor detects that the reading exceeds a threshold (e.g., $50^{\circ }\hbox {C}$), the smart contract could automatically trigger an alert and activate a cooling mechanism. This enables automation and trustless execution within SIoT ecosystems. After successful validation, the transaction is grouped with other verified transactions to form a new block. This block includes a timestamp, the hash of the previous block, and all the validated transactions thereby maintaining a tamper-proof chain of events. Once the block is created, it is appended to the blockchain in a sequential, chronological manner, ensuring the immutability of past records. This immutability is vital for SIoT systems, as it guarantees that historical data such as trust ratings, service logs, or identity records cannot be altered without detection. Following this, the updated blockchain ledger is propagated across the entire network, ensuring that all nodes remain synchronized. Each node thus retains an identical, verified copy of the ledger, enabling decentralized auditability and verification. This distributed nature makes blockchain inherently resilient to failures and attacks, as there is no single point of control or compromise. Finally, the transaction is marked as complete. It becomes a permanent part of the ledger and can be used for future queries, audits, or cross validation in trust management protocols. Throughout this workflow, blockchain enforces several key security principles: cryptography ensures confidentiality and authentication; decentralization eliminates single points of failure; and transparency coupled with immutability upholds data integrity and traceability. This entire workflow collectively enhances the trustworthiness, autonomy, and security of interactions in SIoT networks. Shows the typical Blockchain workflow cycle refer to Fig. 13. Reference^135^ illustrate the blockchain transaction process using a block structure that includes cryptographic hashes, timestamps, Merkle trees, and consensus validation. Each transaction is recorded immutably, forming a tamper-proof, distributed ledger that supports secure data acquisition and storage in decentralized environments.

#### Modular integration strategy for application-specific SIoT systems

Not all SIoT applications require all three technologies simultaneously. Depending on system constraints and priorities such as latency sensitivity, resource availability, or privacy developers may combine any two technologies for a customized solution. This modular approach allows scalable and cost-effective SIoT deployment tailored to specific needs. As summarized in Table 12, recent SIoT systems integrate **Blockchain**, **AI/ML**, and **Edge/Fog/Cloud** technologies to achieve trustworthy, real-time, and privacy-preserving analytics across domains such as healthcare, smart manufacturing, and urban waste management, while facing trade-offs in latency, synchronization, and energy efficiency. Overall, these hybrid integrations illustrate the evolving trend of multi-technology convergence in SIoT, seeking to balance *trust*, *explainability*, and *efficiency* within heterogeneous environments.

1. **Blockchain + AI/ML + Cloud or Edge/Fog Integration** This integration combines blockchain’s decentralized trust and data integrity features with AI/ML’s predictive intelligence, and anchors them within edge/fog nodes to enable real-time, secure, and autonomous decision-making close to the data source.
  Blockchain enhances SIoT with decentralization, immutability, and automated trust mechanisms, providing benefits like tamper-proof device identities through Decentralized Identity, automated access control via Smart Contracts(enforce access rules and automate trust updates), and secure interaction logs on an Immutable Ledger. However, it faces limitations such as high latency and energy consumption, particularly on public chains, and not adaptability and intelligence on its own, making it less suitable for real-time, resource-constrained IoT environments. Reference^25^ proposed SecureSIoTChain, a blockchain-based security framework for SIoT that integrates Graph Neural Networks (GNN) and R-ECDSA for relationship-aware trust inference, secure communication, and decentralized device authentication. The model achieves 95% accuracy and outperforms existing methods in throughput, latency, and trust metrics. In this study they^136^ proposed a blockchain-based authentication framework for secure IoT networks, using a permissioned ledger to manage decentralized device identities and ensure lightweight authentication. The system improves identity verification and reduces reliance on centralized authorities, but still faces challenges in scalability and storage overhead.
  In this work^137^ they proposed CyberGuard, a hybrid framework that integrates blockchain-based trust management with machine learning (SVM, KNN, RF) for secure and efficient resource allocation in edge and fog computing. The system leverages Trust2Vec embeddings and ensemble learning to achieve high prediction accuracy (F1-score: 98.18%) while ensuring data integrity through blockchain. Though effective, the framework introduces computational and storage overhead, and its scalability in real-time environments remains a challenge. Ideal for systems needing trust analytics and audit trails (e.g., autonomous fleets).
  This paper^138^ introduces a blockchain and AI-driven secure communication framework for smart home networks, integrating Firebase-based blockchain authentication to maintain tamper-proof transaction records and leveraging neural networks with the Dragonfly Algorithm to classify transactions as Smart T (trusted), Mod T (moderate), or Avoid T (risky). Cloud-based data processing supports real-time evaluation and ranking, achieving 96.54% accuracy in detecting false authentications while reducing computational complexity by 10.14% compared to existing methods. The proposed solution, validated through 20,000 simulations on Matlab and Google Colab without a physical testbed, outperforms RTS-DELM and data fusion techniques in both security and efficiency, significantly strengthening smart home security and optimizing IoT communication.
  They^139^ present BlockFaaS, a blockchain-enabled, serverless computing framework designed for AI-driven IoT healthcare applications, specifically targeting heart disease risk prediction. The framework integrates a high-performance XGBoost ML model, a SHA-3 (Keccak)-based blockchain module for ensuring data immutability, and TLS protocols for secure communication. Deployed on Google Cloud Functions, BlockFaaS addresses the limitations of resource-constrained IoT devices by offloading computation to the serverless cloud while preserving data integrity, privacy, and scalability. The authors compare BlockFaaS against existing frameworks (HealthFaaS and AIBLOCK), demonstrating improvements in AUC prediction performance, energy efficiency, and cold start latency analysis under real-world workloads.
  In this study^140^ they proposed a blockchain-based federated learning framework for ECG anomaly detection in IoT, leveraging edge-fog-cloud computing. Their system combines autoencoder-based AI/ML with smart contracts on a Ganache blockchain for secure, decentralized training. Simulation results using iFogSim2 showed edge-layer deployment outperformed fog and cloud in energy efficiency, latency, cost, and execution time while maintaining privacy.
2. **Cloud + Edge/Fog + AI/ML Integration** The integration of Cloud, Edge/Fog computing, and AI/ML technologies forms a synergistic framework that enhances the capabilities of Social Internet of Things (SIoT) applications, particularly in latency-sensitive and intelligence-driven domains like smart healthcare and autonomous systems. Edge and Fog nodes alleviate the computational burden on resource-constrained SIoT devices by performing local processing. This enables real-time decision-making, context-awareness, and bandwidth savings, making them ideal for time-critical applications. However, these nodes often lack built-in mechanisms for transparency, auditability, and secure data anchoring, which raises concerns about data tampering and opaque trust decisions in the absence of blockchain-like support. In contrast, the Cloud Layer offers centralized infrastructure to handle computationally intensive tasks, such as training AI/ML models, performing cross-node data aggregation, and distributing optimized models to edge nodes. While the cloud enables scalability and model refinement, it introduces latency, privacy risks, and centralization vulnerabilities, particularly when sensitive user or device data are transmitted and stored offsite. AI/ML technologies empower the SIoT environment by enabling autonomous behavior analysis, adaptive trust scoring, anomaly detection, and personalized responses. These capabilities allow the system to dynamically learn from interaction patterns, assess trustworthiness, and respond to anomalies in device behavior. However, the deployment of AI/ML in SIoT also introduces challenges. These include a lack of explainability and verifiability of model decisions, susceptibility to adversarial attacks (e.g., data poisoning), and the absence of tamper-proof mechanisms for storing or validating outcomes, which can reduce trust in automated processes. Reference^141^ propose a cloud-fog-edge pipeline for smart agriculture that performs real-time image classification (e.g., tomato disease detection) using a ResNet-based CNN model. The model is trained on the cloud, optimized via TensorFlow Lite and the Tensil framework, and executed on edge FPGA devices (PYNQ Z2). They demonstrate minimal accuracy loss ( 0.83%) and significant latency reduction when executed at the Fog layer. Reference^142^ present an edge-based IoT system using non-wearable sensors and machine learning (Isolation Forest and LSTM) for real-time elderly health monitoring. The system ensures privacy, low latency, and effective anomaly detection, achieving 92.29% accuracy on the CASAS TM029 dataset and featuring a dashboard for caregivers and doctors.^143^ proposed a fault-tolerant fog-based SIoT architecture (FSIoT) that uses Markov chains to model and recover from transient and permanent node failures. By integrating K-means clustering and trust-based node evaluation, the system improves availability, reliability, and fault detection accuracy. The model assumes fog/cloud nodes are fault-free and was validated via simulation on 70 nodes.
3. ** Blockchain + Edge/Fog:** Suitable for secure identity and fast decision-making (e.g., smart factories). For modular integration of blockchain and edge computing in application-specific SIoT systems, Ref.^144^ proposed a blockchain-assisted edge computing architecture tailored for IIoT environments, introducing a novel Proof-of-Authentication (PoAh) consensus mechanism. The architecture leverages smart contracts and lightweight blockchain nodes deployed at the edge, ensuring scalable and trustworthy data sharing, device authentication, and traceability. Their PoAh model, implemented via Hyperledger Fabric and Docker containers, significantly reduces authentication time and energy usage while maintaining a high transaction rate (up to 1273 TPS), making it suitable for resource-constrained industrial systems.
4. **Cloud + edge + AI/ML Integration**
  The authors present a comprehensive framework that integrates IoT, edge computing, cloud computing, and AI/ML to enable real-time, intelligent decision-making. They design and implement hybrid architectures where AI models are trained in the cloud and deployed at the edge for low-latency inference. Practical implementations like DILoCC (for distributed incremental learning) and SHIRS (for smart indoor air monitoring) demonstrate how this integration can be applied in real-world smart city and industrial contexts. The paper also addresses key challenges such as energy efficiency, model compression, federated learning, and ethical AI use^145^.
5. **Digital Twin + Edge/Fog/Cloud + Blockchain:**
  A blockchain-enabled digital twin vehicular edge network (DTVEN) is proposed to enable secure and efficient task offloading in vehicular edge environments. The architecture combines digital twins for real-time monitoring of 3C (computation, communication, caching) resources with a blockchain layer utilizing Delegated Proof of Stake (DPoS) consensus and smart contracts for decentralized coordination. As presented by^146^, an improved cuckoo algorithm is used to optimize task offloading decisions, and a greedy resource allocation strategy is applied to minimize consensus overhead, resulting in lower network cost and enhanced edge cooperation.
6. **Federated Learning + Edge/Fog + AI/ML/ Explainable AI (XAI):**
  This work^35^ introduces a privacy-preserving Federated Learning-based IDS with Explainable AI (XAI), using SHAP values for interpretability across decentralized edge devices. Four clients train ANNs on the CICIoT2023 attack dataset, with FedAvg aggregating models at the server. The global model achieves 88.2% testing accuracy, with UDP identified as the most impactful feature via SHAP. Key metrics include Precision (0.8908), Recall (0.684), and F1-Score (0.705), highlighting strong detection performance and interpretability .
7. **Federated Learning + Edge/Fog :** They^147^ designed a federated blockchain-based authentication scheme specifically for cross-domain IIoT device interactions in smart factories. Their scheme eliminates centralized authorities by using a Hyperledger Fabric consortium chain, with smart contracts handling device registration, mutual authentication, and revocation. Performance evaluation showed the architecture improves trust, security, and scalability, although latency increases with the number of peer nodes. Both works demonstrate the viability of decentralized blockchain-based identity and trust frameworks for IIoT, but differ in consensus design, system scope, and optimization focus.
8. AI/ML+IoT+ Swarm:
  This^148^ study presents a low-complexity waste classification model for smart cities, combining VGG16 feature extraction with a Random Forest classifier optimized by Cat Swarm Optimization (CSO). The model achieves 85% accuracy and 0.85 AUC on a six-class garbage dataset, surpassing SVM, XGBoost, and logistic regression in key metrics. Its efficiency and balanced performance make it suitable for real-time IoT-enabled smart city applications.

**Table 12:** SIoT integration types with purpose, strengths, and limitations.

Work	Integration type	Purpose	Use cases	Strength	Limitations
^140^	Blockchain + AI/ML + Cloud or Edge/Fog	Enhance trust and decision-making through verifiable data and intelligent analytics	Trust score prediction, intrusion detection, anomaly detection	Ensures immutable trust history + intelligent behavior learning	High complexity and model drift; blockchain latency; requires frequent model updates
^146^	Cloud + Edge/Fog + AI/ML	Enable real-time anomaly detection with local edge processing (on Raspberry Pi) and cloud-based visualization	Smart home elderly monitoring system	Real-time ML on Raspberry Pi- Non-wearable passive sensing, Privacy-aware design with dashboard	Used simulated sensor data (not live), Caregiver mobile app not yet developed
^146^	Digital Twin + Edge/Fog/Cloud + Blockchain	Enable cyber-physical mirroring, predictive analytics, and secure control	Smart manufacturing, predictive maintenance, secure twin control	Real-time digital mirroring, decentralized analytics and traceability	High synchronization overhead; model mismatch; digital twin setup cost
^142^	Blockchain + Federated Learning + Edge-Fog-Cloud Computing	Privacy-preserving ECG anomaly detection and real-time decision making	Healthcare IoT; Remote cardiac monitoring with mobile/wearable ECG sensors	Low latency, enhanced privacy, decentralized model training, tamper-proof storage via smart contracts	Increased cost, execution time, energy use due to blockchain overhead; partial energy modeling only
^148^	AI/ML + IoT	Improve waste classification accuracy using deep learning and optimized ensemble learning in IoT-enabled environments	Smart waste management in smart cities with automated waste sorting from images	Low-complexity model with 85% accuracy; CSO optimization improves performance; outperforms traditional models (SVM, XGBoost)	No explicit use of edge/cloud; real-time deployment and scalability not discussed; limited to image-only input

#### Proposed unified conceptual framework for SIoT systems using blockchain–cloud, edge/Fog–AI/ML integration

To overcome the above limitations, we propose a conceptual organization of phases, a unified SIoT framework that leverages the combined strengths of blockchain, cloud computing, edge/fog environments, and AI/ML to deliver an intelligent, secure, and scalable solution for decentralized trust and interaction management. Cloud enables global AI model training and data aggregation, fog and edge layers handle real-time computation and context inference, while blockchain ensures secure identity, immutable logging, decentralized trust, and verifiable service interactions.

- **Blockchain:** Handles identity registration, trust anchoring, and access control through smart contracts.
- **Cloud Infrastructure:** Manages large-scale data storage, global coordination, and long-term trust analytics.
- **Edge/Fog Nodes:** Enable fast, localized decision-making and host AI inference engines.
- **AI/ML Models:** Predict trust, detect anomalies, and assist in smart service recommendations.

**Figure 14:**
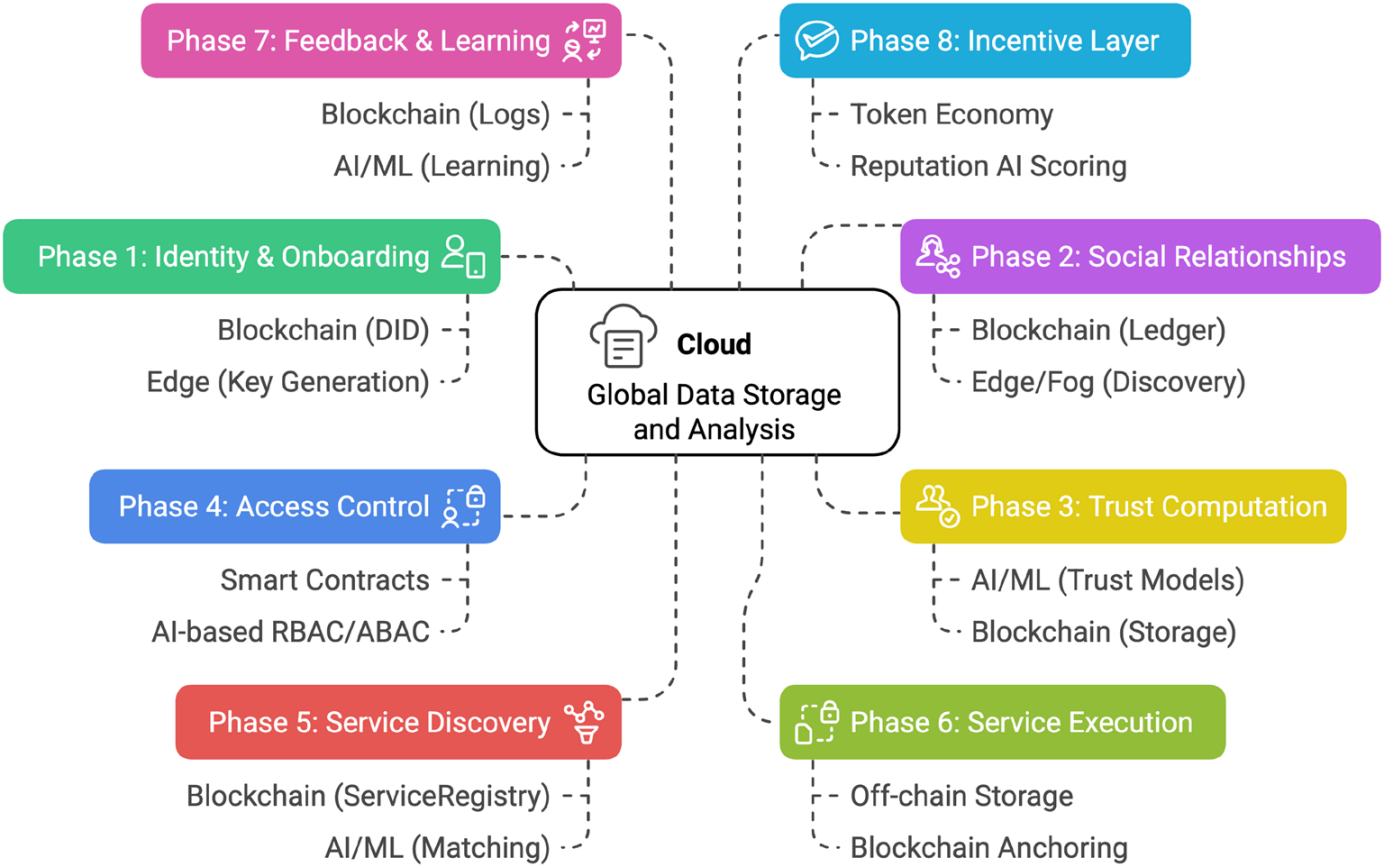
Unified conceptual framework for SIoT.

As shown in Fig. 14, the unified conceptual framework for SIoT is a consolidation of heterogeneous approaches reported in the literature. It is presented as a survey based reference model rather than an implemented design. Prior works propose diverse architectures for blockchain, AI/ML, and edge/fog integration in IoT. For example, They  ^21^ present a three-layer smart city security architecture combining AI-driven anomaly detection with Ethereum based confidentiality and consensus, achieving >98% detection accuracy and  4500 TPS throughput. In this paper  ^19^ they propose an Ethereum based framework for mobile IoT sensors with dual communication modes, trust computation, and cost modeling, demonstrating 38% reduced overhead and 28% lower latency in simulations. Similarly, in this paper they  ^22^ introduced a blockchain enhanced Sensor-as-a-Service (SEaaS) model with modular smart contracts, enabling secure data trading and showing improved efficiency in energy, latency, and throughput metrics. Figure 14 abstracts these functional roles such as identity management, trust evaluation, provenance auditing, and policy enforcement into a single conceptual framework. This consolidation provides a high-level reference view of how these technologies may interoperate in SIoT, without making implementation claims. The diagram illustrates eight key phases.

1. ** Decentralized identity and device onboarding**
  Devices generate decentralized identities (DIDs) and register them on a blockchain, ensuring tamper-proof identification and secure on boarding. This is achieved through local ECDSA (Elliptic Curve Digital Signature algorithm to verify authenticity of data) key pair generation at the edge and registering the DID on a blockchain like Polygon or Fabric, with metadata stored locally or in the cloud if needed, effectively securing against identity spoofing.
2. **Social Relationship Establishment**
  Devices form relationships via edge/fog proximity discovery, registering links on a blockchain as friend, co-location, or service relationships. These relationships are represented as traceable and immutable social links, defined as on-chain mappings that can be optionally tokenized as NFTs for enhanced visibility. Digital signatures facilitate updates and verification, ensuring a secure and transparent network of interactions, while blockchain’s immutable ledger prevents fake or manipulated links, building a reliable social graph.
3. **Trust Evaluation and Reputation Building**
  A dynamic, context-aware trust scoring system is implemented through a smart contract like ReputationManager.sol, storing and updating trust scores based on weighted history, recent, and behavior consistency. Historical interactions calculate on-chain reputation, and malicious behavior is automatically penalized, ensuring a secure and trustworthy network.
4. **Smart Contract Deployment for Access and Service Control**
  Smart contracts like AccessManager.sol, TokenAuthorization.sol, and DelegationManager.sol automate access and service rules, enabling role-based, attribute-based, and token-based access control, as well as social delegation rules. These contracts define service-level access conditions on-chain, eliminating central access control lists and supporting dynamic, rule-based security in a decentralized manner.
5. **Service Discovery and Publishing ** Devices autonomously find and advertise services through the ServiceRegistry.sol smart contract, where they publish services by calling registerService(), emitting events like ServiceRegistered that other devices can listen to. Queryable functions like getServiceByType() and getNearbyServices() enable decentralized lookups, while metadata such as tags, location, and device type enhances matchmaking, ensuring real-time, decentralized, and tamper-proof service discovery.
6. **Secure service interactions** They are enabled through DID-based Verifiable Credentials for device authentication and access control contracts for authorization. Data exchange occurs off-chain, with on-chain anchoring of data hashes ensuring tamper-evidence, and optional encryption provides confidentiality for data in transit and at rest, resulting in scalable, secure, and private service transactions.
7. ** Feedback Logging and Reputation Update** Interactions are logged on-chain for traceability and trust computation, allowing devices to submit feedback that updates trust scores and flags misbehavior, thereby ensuring auditability, accountability, and dispute resolution through immutable records.
8. **Incentivization and Token Economy (Optional)** A token economy is established through an ERC-20 or ERC-721 token contract, rewarding devices for verified services, uptime, and good behavior, while tokens can be used for resource requests, service fees, or trust upgrades, fostering cooperation and sustainability in decentralized environments.

#### Cost and feasibility considerations of blockchain in SIoT

Blockchain adoption in SIoT applications is hindered by regulatory uncertainty, organizational readiness, and cost concerns. Challenges include varying regional regulations, standardization, and lack of internal expertise, while viable workarounds include private blockchains, Layer-2 solutions(eg., Ploygon, IOTA for lightweight deployments), and off-chain data storage . Ultimately, blockchain should be adopted strategically, focusing on applications that require tamper-evidence, decentralized identity, and global trust anchors, rather than universally applying it to all SIoT applications.

#### Other key technologies for decentralized SIoT without blockchain

In scenarios where blockchain integration is impractical due to cost, resource constraints, or energy limitations, several other technologies can support decentralized SIoT architectures. These alternatives help maintain distributed intelligence, trust coordination, and peer-to-peer communication, even in the absence of a global ledger. These technologies offer modular, scalable, and cost-effective pathways to decentralization. While they may not guarantee the same tamper-evidence or global consensus as blockchain, they provide flexible design options for SIoT applications where decentralization is desired but blockchain is infeasible.

1. Federated Learning: Enables multiple devices to collaboratively train machine learning models without sharing raw data, preserving privacy and distributing computational load. Federated learning has been increasingly adopted for privacy-preserving intrusion detection in IoT systems. Reference^149^ proposed an FL-based IDS integrating deep learning and chimp optimization, achieving superior detection accuracy across distributed IoT devices. Reference^150^ proposed a novel FL-IoT framework combining federated learning with TinyML for resource-constrained microcontrollers, enabling efficient, privacy-preserving model training and inference at the edge.
2. Swarm Learning: A fully decentralized variation of federated learning that eliminates the need for a central coordinator by using peer-to-peer consensus. Reference^151^ present MatSwarm, a swarm learning-based framework that integrates federated learning, blockchain, and trusted execution environments (TEEs) to enable secure, decentralized model training across multiple institutions. Unlike traditional FL, MatSwarm removes the need for a central aggregator by using blockchain-based consensus. It employs a swarm transfer learning method to improve generalization on non-i.i.d. datasets and uses Intel SGX to safeguard data integrity and confidentiality. Validated on real-world materials data, MatSwarm demonstrates strong resilience to poisoning attacks and superior performance in accuracy, scalability, and security, highlighting its effectiveness in multi-party computation for sensitive scientific domains.
3. Gossip Protocols: Employ probabilistic message spreading where each device shares updates with a few randomly selected peers, useful for distributing trust scores or alerts. Gossip protocols offer a lightweight, fully decentralized alternative for knowledge dissemination in SIoT environments. Reference^152^ introduced a generic coordination model leveraging a gossip mechanism for decentralized learning in microgrids. Their approach supports two variants such as Gossip Federated Learning (GFDL) and Gossip Ensemble Learning (GEL), allowing nodes to exchange model weights or prediction outputs, respectively. These methods ensure privacy (data never leaves the node), scalability, and flexibility in dynamic edge environments making them promising for SIoT trust-building, anomaly detection, and behavior prediction without relying on blockchain.
  .
4. Multi-Agent Reinforcement Learning (MARL): Distributed agents learn behaviors through local interactions and coordination, suitable for dynamic trust and decision-making.^153^ demonstrated that Multi-Agent Deep Reinforcement Learning (MADRL), when combined with edge computing, significantly improves SIoT network navigability and service recommendation performance by adaptively optimizing friendship paths and enabling real-time, personalized decision-making in decentralized environments.
5. Peer-to-Peer (P2P) Overlay Networks: Enable devices to form mesh-like topologies for direct communication, resource sharing, and decentralized service discovery. Recent advancements in peer-to-peer overlay structures such as TSPeer leverage sensor fingerprinting to enhance trust and reputation in mobile SIoT environments without relying on blockchain mechanisms^154^.
6. Publish–Subscribe Systems (e.g., MQTT): Support asynchronous messaging and event-driven communication among distributed nodes, minimizing centralized dependencies. A secure and decentralized publish/subscribe system was proposed by^155^, integrating topic-based pub/sub messaging with a distributed P2P overlay using hash chains for end-to-end security, suitable for trustable SIoT applications even without blockchain.

#### Recent studies leveraging blockchain technology in IoT and SIoT environments(2023–2025)

**BMIS (Blockchain-based Mobile IoT System)**  logs real-time sensor data on Ethereum for data traceability.^97^ introduced a Blockchain-Based Mobile IoT System (BMIS) that combines a modular multi-sensor device with cloud and blockchain integration. The system enables real-time monitoring through ThingSpeak and tamper-resistant storage via Ethereum smart contracts. This dual-path architecture enhances the trustworthiness, traceability, and mobility of IoT data collection systems. They^156^ proposed DrunkChain, a blockchain-enabled IoT system for preventing drunk driving by continuously monitoring blood alcohol levels and driving behavior. The system ensures secure, immutable data transfer to a central police account using the Algorand blockchain, showcasing the effective integration of blockchain in vehicular IoT and trust-critical SIoT applications.

**Physical Unclonable Functions (PUFs)**  Blockchain-based mutual authentication schemes using PUFs have been proposed to resist cloning and impersonation attacks. A lightweight, blockchain-based mutual authentication and key agreement protocol designed for cross-domain IIoT systems with digital twin integration is presented in^157^. The solution combines Physically Unclonable Functions (PUFs), smart contracts, and a blockchain ledger to ensure decentralized and tamper-resistant authentication. Formal and informal security analyses confirm the protocol’s resistance to various IIoT-specific threats. Recent access control models leveraging blockchain have ensured secure service invocation and user-device interactions in vehicular and smart home SIoT networks. For instance, in^158^ proposed a zero-trust framework combining blockchain, smart contracts, and inner-product encryption to enforce fine-grained access control and decentralized identity management across domains such as smart homes and vehicular systems, ensuring efficient and tamper-resistant service interactions in a 6G-enabled SIoT environment.

### Edge, cloud/ fog computing

In this subsection, we discuss the different types of cloud services and the typical three-layer architecture comprising edge, fog, and cloud computing, as briefly outlined below. Edge, fog/cloud computing collaboratively enhance the scalability, responsiveness, and intelligence of SIoT systems. Cloud platforms provide centralized resources for large scale storage, big data analytics, trust modeling, historical behavior analysis, and global service management. In contrast, edge and fog layers are deployed closer to devices to enable real-time decision-making, local trust evaluation, and context-aware service delivery. This layered architecture reduces latency, bandwidth usage, and response time for sensitive tasks, while offloading computation from resource-constrained devices and supporting hierarchical processing. It also improves fault tolerance and system scalability. However, challenges persist, including maintaining data consistency across layers, enforcing security at multiple distributed points, and managing the cost and complexity of deploying and orchestrating fog and edge infrastructure.

**Figure 15:**
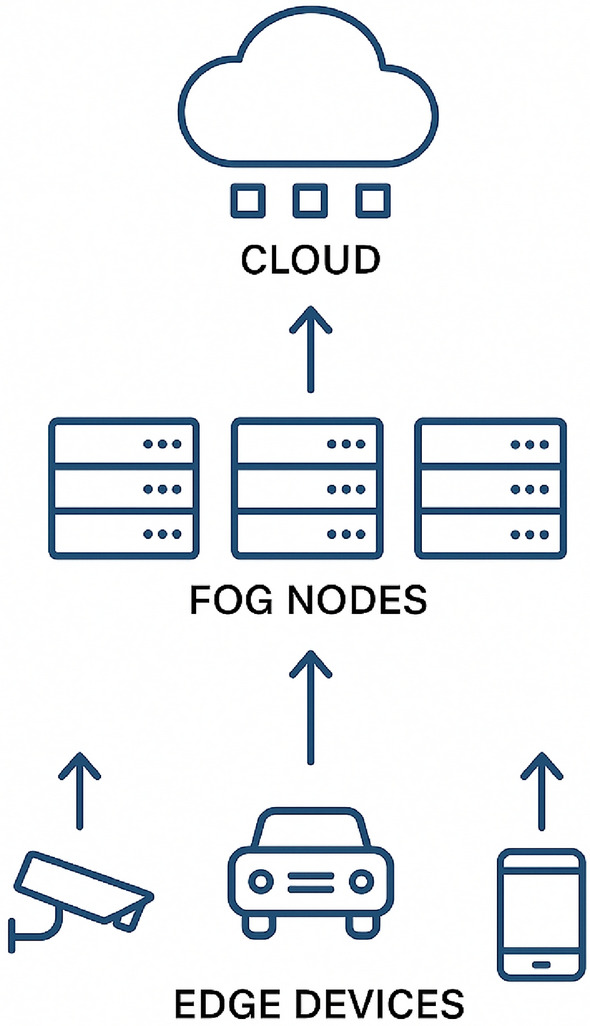
Edge, fog and cloud computing layers.

Figure 15 shows a typical three-layer architecture comprising edge, fog, and cloud computing. Each layer is responsible for specific tasks to ensure efficient data processing, communication, and service delivery within IoT and SIoT environments.

1. Edge devices: This layer captures and generates real-time data, with limited processing power, storage, and computing capabilities. Examples include CCTV cameras, autonomous vehicles, and smartphones.
2. Fog Nodes: Key responsibilities of fog nodes include preprocessing and filtering data, performing latency-sensitive analytics, and acting as an intermediary between edge devices and the cloud. Examples include local servers, gateways, and routers.
3. Cloud: The cloud is responsible for centralized data storage, processing, and long-term analytics, including machine learning model training. Examples include remote data centers and cloud infrastructure. The cloud offers high scalability, massive computational resources, and a global data view, enabling efficient processing and analysis of large-scale data.

**Types of Cloud Services** Different types of cloud services are briefly mentioned in the below.

1. IaaS (Infrastructure as a Service): Provides virtualized hardware and storage (e.g., AWS EC2).
2. PaaS (Platform as a Service): Offers tools for application development and deployment.
3. SaaS (Software as a Service): Delivers end-user services like dashboards and social interfaces.

#### Recent studies leveraging edge and computing in IoT and SIoT environments (2023–2025)

This study^159^ demonstrates how edge–cloud combined can address the computational and latency demands of dynamic IoT applications, particularly in mission-critical domains like smart energy grids. Reference^160^ propose a novel Edge/Cloud architecture tailored for the Social Internet of Things (SIoT), enabling the integration of Virtual Users (VUs) and Social Virtual Objects (SVOs) through a containerized microservices infrastructure. Their solution automates deployment, supports user clustering, and enables low-latency service migration via edge computing. The architecture addresses scalability, security, and automation needs, validated through experimental evaluation using AWS-based environments. The study demonstrates performance improvements over traditional platforms like Google App Engine, making it a key recent advancement in SIoT infrastructure design. Reference^161^ implemented a Cloud-Edge-IoT continuum model for Industry 4.0 using EdgeCloudSim and SUMO simulators. Their work demonstrates how edge-cloud architectures can support low-latency task offloading and intelligent transportation use cases, making it a significant contribution to recent advances in IoT and SIoT edge-cloud integrations.

### AI and ML

AI and ML boost the intelligence and self-sufficiency of Social Internet of Things (SIoT) devices, allowing them to learn from interactions and context. Key AI/ML capabilities in SIoT include:

1. ** Trust prediction models:** Assessing device reliability through machine learning-based analysis of social behavior. Machine learning techniques have increasingly been applied to predict trust in vehicular networks by analyzing behavioral attributes such as packet delivery ratio and interaction frequency. In one such study, a trust management heuristic was developed using supervised classifiers like SVM and KNN to identify malicious vehicles in an Internet of Vehicles environment^162^. The model achieved high classification accuracy by first clustering unlabeled data and then applying classification on mean parametric scores to distinguish between trustworthy and untrustworthy nodes.
2. **Anomaly detection:** Identifying unusual patterns or suspicious communication. Recent advances in explainable AI (XAI) techniques have improved the interpretability of trust prediction models for IoT anomaly detection. For instance, Ref.^36^ proposed an XAI-IoT framework that integrates single and ensemble AI models with XAI tools such as SHAP, LIME, and CEM to accurately detect anomalies and explain model predictions in both sensor-based and network-based IoT systems.
3. **Context-aware recommendations:** Providing personalized service suggestions based on learned preferences and device usage history. Reference^163^ proposed a Triple Attentive Neural Network (TANN) combining context-aware session similarity and frequent graph pattern mining for recommendation in Smart EMS. While not IoT-specific, the AI/ML-driven framework is adaptable for session-based recommendations in SIoT settings involving user-device interactions and contextual session data. They^164^ proposed a new framework called MAFDRL- a recommender system for friendship path selection in SIoT which utilizes optimal policy learning (via DRL and SAC) and preserves privacy (via FL), thereby improving recommendation accuracy and efficiency in large-scale, dynamic SIoT environments.

#### ML techniques

**Figure 16:**
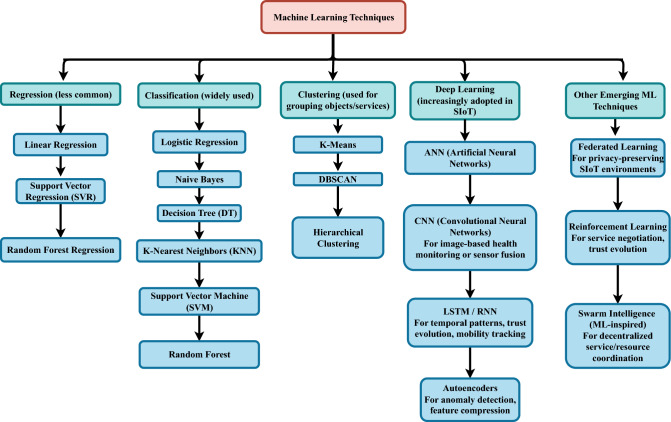
Machine learning techniques commonly used in SIoT.

Machine learning (ML) models in the Social Internet of Things (SIoT) are deployed across edge, fog, and cloud layers to enable dynamic trust evaluation, anomaly detection, relationship classification, and intelligent service/resource discovery. By analyzing historical interactions, social ties, and quality-of-service parameters, ML enhances context-aware service recommendations and improves system responsiveness in dynamic environments. These models classify user-device relationships, detect intrusions through behavioral pattern learning, and support adaptive trust management, reducing reliance on static rules or manual configurations. Despite these advances, ML integration in SIoT faces challenges such as frequent model updates, computational overhead on resource-constrained devices, and vulnerabilities to adversarial inputs and data poisoning. Given SIoT’s heterogeneity and evolving nature, choosing appropriate ML techniques is vital for scalability, privacy preservation, and robust decision-making. Figure 16 categorizes various ML approaches based on their operational goals such as regression, classification, clustering, deep learning, and emerging paradigms. These techniques are widely adopted in SIoT applications to analyze user behavior, predict trustworthiness, group devices, and adapt to temporal patterns in service delivery. A brief description of each technique, along with its typical use in SIoT contexts, is provided below.

1. Regression (less common in SIoT)
  Linear Regression: Linear Regression is a basic model that assumes a straight-line relationship between input and output variables, meaning the output increases or decreases proportionally with the input. In SIoT systems, it can be used to predict trust scores between devices based on the frequency of successful data exchanges, where each additional reliable interaction adds a fixed increment to the trust score, ranging from 0 to 1. This approach is simple, fast, and works effectively as a baseline analysis tool when device interactions follow consistent patterns and the data isn’t too noisy. The researchers^165^ applied linear regression to examine the relationship between several independent variables—perceived usefulness, perceived ease of use, attitude toward SIoT, perceived privacy risk, and trust—and the dependent variable of SIoT adoption. Results showed that perceived ease of use and attitude had a statistically significant influence on SIoT adoption, while perceived usefulness, privacy risk, and trust did not. These findings underscore how regression analysis can isolate the most impactful predictors in user behavior modeling.
  Support Vector Regression : Support Vector Regression creates a prediction model that fits data within a margin of tolerance, forming a “trust tube” around expected values that helps focus on significant inconsistencies while ignoring minor deviations. In SIoT systems, it is especially useful for predicting trust scores between devices that may experience occasional delays or minor glitches, as it flexibly handles small hiccups like one-time misfires and flags major issues such as repeated misinformation. SVR excels in managing uncertainty since IoT networks often produce noisy data, it learns from social interaction patterns by modeling relationship strength over time, and it avoids overreacting to isolated bad behavior, making it highly suitable for dynamic and unpredictable environments.
  Random Forest Regression: Random Forest Regression is an ensemble model that uses multiple decision trees, each trained on different features and logic paths, and then averages their predictions to produce a final output. In SIoT systems, it can forecast trust scores by analyzing factors such as interaction frequency, data accuracy, battery level, and network latency, with each tree emphasizing different attributes—for instance, one focusing on latency and another on reliability. This collaborative approach results in a robust trust estimate that effectively captures complex, nonlinear relationships, resists outliers, and adapts well to messy or incomplete device data.
2. Classification
  Logistic Regression: Logistic Regression is a linear classification model that estimates probabilities by fitting input features—such as authentication success rate, interaction duration, energy consumption, and firmware update history—to a logistic curve. In SIoT systems, it’s commonly used to classify devices as “trusted” or “untrusted,” offering quick and interpretable decisions ideal for lightweight, resource-constrained environments. While it’s fast and well-suited for binary classification in linearly separable data, it tends to underperform with complex or nonlinear trust patterns due to its inherent simplicity and limited decision boundaries. A dual-layer strategy to enhance survivability in Industrial IoT systems is proposed through the combination of machine learning-driven device identification and a blockchain-enabled smart contract framework. Reference^166^ evaluated 12 ML models—including LR, SVM, KNN, RF, GB, CNN, and LSTM—and identified LSTM as the most accurate, achieving 98.96% accuracy. Their smart contract architecture further supports secure data exchange and access control in smart home data marketplaces. This hybrid approach reinforces IIoT resilience, trust, and security.
  Naive Bayes: Naive Bayes is a probabilistic classifier built on Bayes’ Theorem, making rapid predictions by assuming that each input feature—such as device manufacturer reputation, data transmission integrity, sensor context (e.g. location and temperature), and unauthorized access frequency—contributes independently to the final trust estimate. In SIoT systems, this model excels in early-stage trust screening using sparse or categorical metadata, offering scalable and efficient performance. While it’s well-suited for tasks like spam detection and document classification, its core independence assumption can oversimplify complex interactions between trust indicators, potentially limiting accuracy in nuanced environments. To address the increasing prevalence of diabetes,^167^ developed a supervised machine learning model to predict diabetes using the Pima Indians Diabetes dataset. The study compares the performance of k-Nearest Neighbors (KNN) and Naive Bayes classifiers across multiple data splits and concludes that Naive Bayes consistently outperforms KNN in terms of accuracy, precision, and recall.
  Decision Tree(DT):Decision Tree (DT) is a highly interpretable, tree-structured classification model that splits data based on feature conditions—perfect for rule-based trust systems in SIoT. It can assess trustworthiness using inputs like peer interaction frequency, security patch history, latency thresholds, and anomaly scores, leading to decisions such as “If latency $> 400$ms and anomaly score $> 0.6$ $\rightarrow$ Untrusted.” DTs are versatile, handling both numerical and categorical data, and are valuable for exploratory analysis and rapid prototyping. However, they are prone to overfitting and may generalize poorly in complex or noisy SIoT environments unless combined with techniques like pruning or ensembles.
  K-Nearest Neighbours(KNN): It is a lazy learning algorithm that classifies devices by comparing them to the closest instances in feature space—leveraging patterns like firmware version, network reliability, trust rating history, and CPU usage. In SIoT systems, it supports neighborhood-based trust inference where a new device inherits trust labels from its most similar peers; if most neighbors are trusted, it likely is too. This simplicity allows KNN to adapt dynamically without a training phase, making it suitable for well-clustered trust datasets. However, it becomes computationally heavy with large data and is sensitive to irrelevant features and inconsistent scaling, requiring careful preprocessing to maintain accuracy. Building on traditional KNN approaches, this study^168^ adapts the algorithm to handle mixed data types—numeric and string—allowing for more nuanced classification of plant commodities based on environmental profiles
  Support Vector Machine(SVM):
    SVM is a powerful classification model that identifies the optimal boundary—or hyperplane—to separate data points, often using kernel functions to handle complex, non-linear relationships. In SIoT systems, it’s effective for discerning trustworthiness based on rich features like signal strength anomalies, conflict resolution history, access control outcomes, and multimodal trust scores. By mapping data into higher-dimensional space, SVM creates clearer separation between trusted and untrusted devices, yielding high accuracy for binary trust classification. Though it thrives in feature-rich environments, SVM can be computationally intensive and demands careful kernel and parameter tuning, making it better suited for smaller, high-stakes datasets like bioinformatics or nuanced trust modeling. Reference^169^ applied an SVM classifier optimized using the Pelican Optimization Algorithm for real-time gender identification from facial video frames. While the work is not SIoT-specific, its use of human-centered, real-time visual data and edge-deployable machine learning aligns with many SIoT use cases such as surveillance and social device interaction, showcasing how SVM can be enhanced for improved classification accuracy and reduced latency in intelligent IoT systems.
  Random Forest: Random Forest is a collective approach that builds multiple decision trees using varied subsets of trust features—such as interaction frequency, data accuracy, battery status, and network latency—and then combines their predictions to produce a strong, reliable classification. In SIoT systems, this method captures diverse aspects of trust, with each tree focusing on a different priority (e.g., one may highlight latency while another weighs energy reliability), resulting in robust trust evaluations even with noisy or incomplete data. Random Forest models are especially effective for handling complex classification tasks and collaborative trust analysis, though they tend to require more computational power and can be harder to interpret compared to using a single decision tree. This^170^ paper presents a predictive framework that combines IoT-based environmental sensing with machine learning models to forecast forest fires. The study utilizes weather and historical fire data, employing algorithms like Random Forest, XGBoost, KNN, Decision Trees, and Logistic Regression. The XGBoost and Random Forest models achieved the best performance, with accuracies up to 97.52%. IoT devices (e.g., sensors and drones) provide real-time environmental data (temperature, humidity, wind speed, etc.), enhancing the model’s responsiveness and accuracy. The integration supports early fire risk detection, resource optimization, and improved decision-making in wildfire management.
3. Clustering
  K-means:K-means is a partition-based clustering algorithm that divides data into k distinct groups, each defined by a central point called a centroid. It works by iteratively assigning data points—such as SIoT device metrics like communication latency, interaction frequency, and energy usage—to the nearest centroid and adjusting those centroids until the clusters stabilize. This makes it useful for grouping similar devices (e.g., low-energy sensors vs. high-throughput routers) to optimize trust assessment and service efficiency. While K-means is fast, scalable, and simple to implement—especially effective for spherical clusters—it requires predefining k, struggles with irregular cluster shapes, and is sensitive to outliers and how centroids are initially placed. It’s well-suited for behavioral clustering, performance-based device grouping, and load balancing in IoT networks. A novel cluster-based aggregation model for the Social Internet of Things (SIoT) was proposed that integrates relationship-aware cluster head selection using Decision Tree algorithms with K-Means clustering and Huffman coding for data compression. The model selects cluster heads based on object relationships and profiling features, compresses data at the cluster head, and forwards it to the sink node, significantly improving energy efficiency, network lifetime, and aggregation accuracy. Simulations conducted using the SIoT-CCN simulator demonstrated superior performance over existing clustering and aggregation methods in terms of BIC scores, silhouette scores, and transmission overhead^171^.
  DBSCAN : DBSCAN (Density-Based Spatial Clustering of Applications with Noise) is a clustering algorithm that groups closely packed data points together while marking isolated or low-density points as outliers—making it especially useful for discovering natural data structures without needing to predefine the number of clusters. In SIoT systems, it can effectively detect clusters of trusted devices based on trust score proximity and highlight compromised or anomalous behavior as noise. DBSCAN excels at identifying irregularly shaped clusters and automatically detecting outliers, which is ideal for anomaly detection and spatial analysis in smart environments. However, its performance depends heavily on selecting appropriate distance and density parameters, and it may struggle with datasets where cluster densities vary significantly. In their study^172^ they applied DBSCAN clustering to group geographically proximate IoT-enabled bike-sharing stations, enabling socially contextual forecasting of shared mobility demand. Though not explicitly framed within the SIoT paradigm, the clustering of physical IoT nodes and subsequent demand prediction reflects spatial-social grouping logic relevant to SIoT applications in smart transportation. A two-step community detection algorithm was proposed for efficient service provisioning in SIoT, combining Louvain for social structure analysis and DBSCAN to refine communities by removing outliers and merging spatially dense device clusters. Reference^173^ demonstrated that this DBSCAN-enhanced stage improves modularity, execution time, and the quality of service composition, enabling faster and more scalable service discovery within socially connected IoT networks.
  Hierarchical Clustering : Hierarchical Clustering builds a layered structure of clusters by either progressively merging similar data points (agglomerative) or recursively splitting larger groups (divisive), often visualized through dendrograms that depict nested relationships. In SIoT systems, this approach supports creating trust hierarchies—such as grouping devices first by manufacturer and then further dividing them by interaction behavior or security profiles—making it valuable for semantic reasoning and ontology structuring. While the method offers interpretable cluster trees and avoids needing a predefined cluster count, it can be computationally intensive, sensitive to noise and feature scaling, and challenging when deciding where to “cut” the hierarchy to form final clusters. It shines in domain-driven clustering, trust modeling, and taxonomy development. Reference^174^ introduced a hierarchical clustering-based FL framework (FedCHAR) that identifies similarities among distributed users to improve personalization and robustness. Their method, though designed for HAR, is adaptable to SIoT, where clustering socially connected objects can improve collaborative intelligence, fairness, and security in a decentralized setting.
4. Deep learning
  ANN: Artificial Neural Network (ANN) ANNs are the foundational deep learning models inspired by biological neurons. They consist of multiple interconnected layers of nodes that learn patterns by adjusting weights during training. In SIoT systems, ANNs can predict trust scores or detect anomalies from features like transmission rates, authentication logs, and latency patterns. They are flexible for various tasks, easy to scale, general-purpose model however may require large data for training, less suited for spatial or sequential patterns . They are suitable for basic trust estimation, sensor fusion, generic pattern recognition.^175^ propose semantic rules for service discovery in SIoT, and evaluate multiple ML classifiers - Decision Tree, Naive Bayes, KNN, and ANN to predict health services and discover context-aware resources based on object relationships. ANN, KNN, and DT showed high performance (100% in most test ratios). The model leveraged object-object and user-object relationships for context-aware service discovery.
  CNN: Convolutional Neural Network (CNN), CNNs are specialized for spatial data and use convolutional layers to detect local patterns. Though famous for image tasks, they’re also effective in analyzing structured data like time-series sensor outputs. In SIoT, CNNs can detect localized anomalies or behavioral deviations in network traffic graphs or sensor maps. Excellent at capturing local features, efficient with grid-like data . However less suited for sequential data without modifications. They are well suited for Visual data analysis, spatio-temporal trust modeling, anomaly detection in device states. Reference^176^ proposed a feature selection framework for SIoT that integrates TransCNN—a deep learning model combining Transformer and CNN layers—with the Chaos Game Optimization (CGO) algorithm. TransCNN extracts robust features from both text and numerical data, while CGO identifies the most relevant ones to boost classification performance. By blending representation learning with heuristic-driven selection, the model adapts to diverse SIoT tasks. Tested on eight datasets, it surpassed ten leading methods in accuracy, sensitivity, specificity, feature count, and fitness value, proving its effectiveness in reducing dimensionality while preserving predictive strength.
  LSTM/RNN: Recurrent Neural Networks (RNNs) and their enhanced variant Long Short-Term Memory (LSTM) networks are deep learning architectures specifically designed to handle sequential and time-dependent data. Unlike traditional models that treat each input independently, RNNs maintain internal states that persist across input steps—allowing them to capture temporal dependencies. LSTMs improve upon standard RNNs by integrating memory cells and gates that help them remember long-term relationships and prevent issues like vanishing gradients during training. In SIoT systems, these models are ideal for modeling evolving trust behaviors, forecasting device interactions, or detecting changes in communication patterns over time. Their strength lies in effectively learning patterns from sequences such as authentication histories or periodic sensor anomalies. However, training these models can be computationally intensive, and managing long sequences or tuning parameters may require expertise and careful design. A hybrid LSTM-RNN model optimized with Lion Optimization is proposed for IoT-based cardiac patient monitoring, achieving 99.99% accuracy. The system enables real-time analysis of vital signs and enhances prediction performance through intelligent feature selection^177^.
  Autoencoders:Autoencoders Autoencoders learn to compress and reconstruct data, capturing essential features while filtering noise. They’re powerful for unsupervised anomaly detection in SIoT, flagging deviations in trust scores or sensor data. A well-trained autoencoder reconstructs typical behavior accurately, while anomalies trigger reconstruction errors. They are unsupervised learning, effective in anomaly detection and dimensionality reduction . They May fail with highly noisy data, interpretability can be limited . Suitable for feature compression, trust anomaly detection, privacy-aware representation learning. The study^178^ propose an autoencoder-based malware analysis framework that leverages grayscale and RGB imagery representations of malware to enhance IoT security in Smart Cities. Their approach investigates various autoencoder (AE) architectures, including convolutional variational AEs, to classify malware efficiently. Experiments demonstrate that the method is robust across different input shapes and supports multi-label classification, making it suitable for complex Smart City IoT environments.
5. Other Emerging ML Technique
  Federated Learning: A decentralized approach to training machine learning models across multiple devices or nodes without transferring raw data to a central server. Instead, each device trains its own model locally and shares only the learned parameters, preserving data privacy. This is particularly powerful in SIoT systems, where trust evaluation or behavior analysis can be collaboratively learned across edge devices—such as smart sensors or IoT gateways—without compromising sensitive personal or network-level information. It addresses both scalability and privacy concerns, though challenges like non-uniform data distribution and limited computational resources across devices require thoughtful model design and aggregation strategies. To mitigate the high communication burden typically seen in decentralized federated learning (DFL) systems with numerous interconnected social nodes in Social Internet of Things (SIoT) environments, a dynamic multi-cluster DFL (DMC-DFL) framework was developed by^179^, utilizing a Limited Label Propagation Algorithm (LLPA) for adaptive clustering. This communication-optimized approach is tailored for networks with evolving topologies and features a training workflow comprising local updates, intra-cluster coordination, and inter-cluster communication. Extensive experiments on four datasets showed that the proposed method substantially improves communication efficiency and training performance compared to existing DFL benchmarks.
  Reinforcement Learning: It involves training agents to make sequential decisions by interacting with an environment and receiving feedback in the form of rewards or penalties. In SIoT contexts, RL can be used to optimize trust-aware routing, adaptive access control, or autonomous decision-making for devices that learn from long-term outcomes. For example, a node might learn to avoid untrustworthy neighbors or reward collaborative behavior over time. RL excels at modeling dynamic interactions but often demands substantial exploration and tuning, making it computationally intensive and occasionally unstable in large, noisy environments. Reference^153^ devised an edge-centric service recommendation framework for Social IoT (SIoT) systems, leveraging Multi-Agent Deep Reinforcement Learning (MADRL) to optimize friendship path routing and service discovery. The system incorporates decentralized edge caching and cooperative multi-agent learning to enable scalable, low-latency, and context-aware recommendations. Experimental results reveal that the framework outperforms existing approaches in accuracy, operational efficiency, and adaptability across dynamic SIoT environments.
  Swarm Intelligence: Draws inspiration from collective behavior in nature—like ant colonies or bird flocks—to develop distributed problem-solving systems. In SIoT, swarm-based algorithms can be used for decentralized trust computation, resilient network formation, and resource-aware task allocation where individual devices act locally but contribute to a coherent global strategy. The strength of swarm intelligence lies in its adaptability, fault tolerance, and scalability, especially in unpredictable or resource-constrained conditions. However, designing effective coordination mechanisms and ensuring convergence across diverse agents can pose challenges as system complexity increases. Reference^180^ proposed a swarm intelligence-driven method for feature selection in SIoT systems by integrating quantum-inspired enhancements into the Artificial Hummingbird Algorithm (AHA). The upgraded Quantum AHA (QAHA) achieved a more effective exploration–exploitation trade-off and surpassed eight competing metaheuristic algorithms in terms of accuracy and dimensionality reduction across benchmark and real-world SIoT datasets.

#### Recent studies leveraging AIML in IoT & SIoT environments (2023–2025)

Federated learning approaches have been explored to preserve user privacy while training models across distributed SIoT devices.^181^ proposed and implemented SIDS, a trust-aware federated intrusion detection system for SIoT. They introduced a GAN-based poisoning attack to demonstrate federated learning vulnerabilities and validated their approach using real-world datasets. This study implements a federated hybrid deep learning framework tailored for distributed IoT edge environments, preserving privacy and improving intrusion detection accuracy through FHDBN and optimization techniques^182^. Deep learning-based frameworks have been adopted for profile inference and malicious node detection. Reference^183^ RIOT-ML provides an open-source toolkit that enables the deployment, evaluation, and secure updating of TinyML models on low-power IoT devices, contributing to real-world applications of AI/ML in resource-constrained environments. A deep learning-based framework called EITM was proposed and implemented for effective node identification in SIoT networks, which employs node embedding techniques and LSTM models to select influential nodes^184^. The method was evaluated using real-world SmartSantander datasets and compared against conventional baselines using multiple performance metrics.

## Security techniques

This section addresses **RQ**7, by reviewing various security techniques employed in SIoT systems, including data protection mechanisms, blockchain-based solutions, access control models, trust management strategies, privacy-preserving methods, and secure communication protocols. These techniques form the foundational components for securing the Social Internet of Things (SIoT), as supported by the cited literature. A detailed comparison of evaluation strategies is presented in Table 13, while a comprehensive review of the security techniques is summarized in Table 14.

**Table 13:** Summary of evaluation of security techniques in SIoT literature.

Ref.	Domain	Eval. type	Tools used	Lang.	Metrics	Result
^185^	Smart Grid	Prototype	Raspberry Pi 4B, Flask	Python 3.11	Auth. time (ms)	$\sim$46 ms @256-bit; scales to 2048-bit
^26^	Healthcare (EHRs)	Simulation	Hyperledger Caliper, Solidity	Solidity (Eth)	Throughput, cost, utilization	Secure, efficient; fixed signature size
^25^	Social IoT	Simulation + Comparative	Python stack (SciPy, SKO, etc.)	Python	Accuracy, latency, trust, privacy	95% acc., 280 Tx/s, 2.2s latency, high trust/privacy scores
^186^	Healthcare	Simulation	Smart Contracts, IPFS, Ethereum/Polygon	Solidity	Doc. verify time ( 2.2s), security, automation	Fast, decentralized, tamper-proof verification
^187^	Access Control	Prototype	Hyperledger Fabric/Besu, CouchDB, AWS	Solidity/Go	Access latency, trust accuracy, cert verify time	<200ms avg. access; fast onboarding, RSA+KYC used
^188^	Group Decision	Simulation + Case Study	MATLAB (custom)	Not specified	GCD, ICD, preference plots	GCD $\ge 0.901$ in 2 rounds; outperforms baselines
^189^	Cloud Offloading	Conceptual projection	SSIM, C2C, RMSE, diff. ops	Python, MATLAB	Reduction ratio, recon. accuracy	High data savings; generalizable across IoT types
^190^	Gateway Security	Real Testbed	Raspberry Pi-2, Mbed OS, Wireshark	C	MPT, TPT, DTLS overhead, Power	Scales linearly; TPT $\approx$ 2.5s; $\sim$3.8MB/thread; low power overhead; blocks illegal clients

### Data security mechanisms

This subsection presents a review of existing security mechanisms namely, authentication and authorization, encryption techniques, and methods ensuring data integrity and confidentiality as referenced in the cited literature.

1. **Authentication and Authorization** Authentication verifies the identity of a device or user (e.g., a smart lock recognizing the owner’s phone). Authorization determines what actions the authenticated entity is allowed to perform (e.g., only the homeowner can unlock the door, while guests are restricted). In the context of secure device access for IoT-based smart home environments, Ref.^191^ introduce an authentication and key agreement scheme based on Modified Honey Encryption and Elliptic Curve Cryptography. The scheme ensures authorized access through session key negotiation and identity verification, while resisting various attacks such as replay, eavesdropping, and impersonation. A smart delegation mechanism was proposed to enhance authorization in SIoT by selecting delegatees based on social links, delegator behavior, and history, reducing overhead while maintaining flexibility^23^.The authors propose a novel quantum-resistant authentication system for smart meters in smart grids, using a hybrid RSA with One-Time Pad (OTP) approach. Unlike traditional RSA, their method changes the encryption key modulus ($n_i$) for every session and never discloses it, achieving dynamic authentication as required by the EU NIS2 directive. They introduce an efficient key exchange protocol based on transmitting only the difference ($\Delta n_i$) between session keys, ensuring absolute security even against quantum attacks. The system was implemented and tested on a Raspberry Pi, showing practical performance ($\sim$50 ms) for 256-bit security, suitable for low-power IoT devices ^185^.
2. **Encryption**:It is the process of converting readable data (plaintext) into an unreadable format (ciphertext) to protect it from unauthorized access. Only someone with the correct decryption key can convert the ciphertext back into plaintext.^26^ developed a blockchain-based hybrid encryption scheme combining ECDSA and Dilithium to enhance data security and resist quantum attacks, offering valuable encryption mechanisms for SIoT systems.
3. **Data integrity and confidentiality** These are pivotal concepts in SIoT to ensure that transmitted or stored data remains accurate, consistent, and unaltered, and to guarantee that sensitive information is kept private and accessible only to authorized devices or users. In the event that a device receives tampered data, it might make erroneous decisions, such as mistakenly unlocking a smart door. Reference^192^ proposed a blockchain-based security framework that integrates decentralization, smart contracts, and federated learning. It is designed to offer a tamper-proof, scalable, and low-latency solution for vehicular networks, focusing on data confidentiality, integrity and attack resilience in the Internet of Vehicles (IoVT). The authors^25^ present SecureSIoTChain, a blockchain-based security framework for the Social Internet of Things (SIoT). It combines Graph Neural Networks (GNN) for dynamic relationship inference with Relationship-Elliptic Curve Digital Signature Algorithm (R-ECDSA) for secure device authentication and communication. This framework ensures data confidentiality, integrity, and anomaly detection, achieving high performance with 95% accuracy and 96% precision. Simulations demonstrate its superiority in throughput, latency, and security metrics.

### Blockchain based mechanism

This subsection reviews blockchain-based mechanisms, highlighting decentralization for distributed control, immutable ledgers for tamper-proof records, and smart contracts for automated rule enforcement, as discussed in existing literature.

1. **Decentralization** Decentralization means removing dependency on a single centralized entity by distributing control and decision making among several nodes.This allows device to communicate independently without supervision. Blockchain technology facilitate decentralization by providing secure data sharing, automated smart contracts, and establishing trust without the need for central authority. It is necessary to achieve decentralization in SIoT by leveraging blockchain technology in order to avoid single point failure and performance bottlneck faced in centralised system. As described in^193^, have introduced a decentralized blockchain-based solution that incorporates a censorship-resistant mechanism, ensuring unrestricted data flow from sensors to the blockchain and from the blockchain to actuators. The study^7^ evaluated three trust management models (centralized, distributed, and blockchain-based), demonstrating the advantages of blockchain-based trust management. It provided an in-depth analysis of IoT classifications (IoMT, IIoT, IoV, and SIoT), requirements, challenges, and applications of IoT. Reference^194^ Researchers have introduced a blockchain-based consensus protocol for securing IoT networks. Using Ethereum as a decentralized platform, they validated their protocol through simulations. This protocol enables IoT devices to participate in consensus, validate transactions, and maintain the blockchain, ensuring network security even in the presence of malicious devices. The protocol operates in stages, including pre-prepare, prepare, and commit, to achieve secure agreement.
2. **Immutable Ledger**
  Immutable ledger offers a great solution as once data is recorded it cannot be altered, this can be benicial in SIoT environment for securing transactional records, device behaviour logs, and access control events. A blockchain-based solution is developed, leveraging immutability, transparency, and decentralization, combined with soulbound tokens, to create a tamper-proof and privacy-preserving identity verification system. A soulbound tokens (SBTs), a non-transferable application of blockchain technology, on the Ethereum Polygon network, combined with cloud computing and IPFS for off-chain storage of medical documents. This approach enables secure, decentralized, and efficient verification of medical records, surpassing traditional NFT-based methods and offering an autonomous alternative to manual or centralized verification processes^186^.This system has potential applications in integration with AI, biometrics, IoT, and Social IoT (SIoT), offering a robust and secure identity management framework. This study^195^ proposes a novel approach integrating AI-driven task distribution with a decentralized mechanism for task assignment and validation, leveraging the Proof of Authority (POA) consensus mechanism. This POA-blockchain-based system, combined with Cloud Internet of Things (CIIoT), addresses key challenges such as task distribution, resource utilization, transaction processing, and scalability. The proposed system aims to minimize resource waste, improve energy efficiency through Dynamic Voltage and Frequency Scaling (DVFS), reduce operational costs and carbon footprint, and enhance overall efficiency.
3. **Smart contract**
  Smart contract refers to self executing a code when certain condition are met without human intervention. Example dispensing a chocolate bar on a vendeing machine. A novel Sybil detection and prevention method, SybilPSIoT has been developed for SIoT environmet, integrating web of trust, signed social networks, smart contracts and game theory to effectively mitigate Sybil attacks^68^. They have^103^proposed a scalabel trust model SCoTMan, for Social Internet of Things environment that integrates smart contract on Hyperledger fabric to handle both trust computations such as direct and direct , while keeping in mind real world constraints.

### Access control mechanism

This subsection reviews existing access control mechanisms, including Role-Based Control (RBC) ensuring access based on predefined roles, Attribute-Based Control (ABC) ensuring fine-grained access through user attributes, and social contextual access control ensuring permissions based on social relationships and context, as supported by the cited literature.

1. **Role Based Access control:** Role based access control is a security mechanism that restricts access based on roles( Admin, editor, user, viewers ) assigned to individual users. In their study^196^ they proposed an efficient and secure data processing framework for role based access control (RBAC).
2. **Attribute Based Access Control:** In attribute based accces control mechanism, the decisions are made on the combination of attributes such as users, resouces, actions, environment/context. This^197^ study, implements a dynamic (time-based) attribute-based access control(ABAC) framework on a private Ethereum blockchain, demonstrating that while initial deployment of four smart contract is costlier than traditional access control lists(ACLs), the ABAC approach proves more gas-efficient for policy updates and attribute management, offering scalabale and cost-effective access control for growing smart city infrastructure. The authors^198^ proposed a dynamic attribute-based access control mechanism , replacing static mechanisms (MAC, DAC, RBAC, and ABAC) with a more flexible and real-time capable solution for device monitoring and access management. They^187^ propose a dynamic attribute-based access control (ABAC) model using a hybrid blockchain architecture (Hyperledger Fabric + Besu) for smart home energy systems. The system integrates KYC verification, smart contracts, and real-time trust recalibration to securely manage device access, prevent unauthorized interactions, and support adaptive policy enforcement in line with zero-trust principles.
3. **Social contextual Access Control **
  Access decisions are based on dynamic, relationship-driven, and behavior-sensitive information, moving beyond traditional static rules such as Role-Based Access Control (RBAC) and Attribute-Based Access Control (ABAC). The proposed model utilizes a deep learning-based approach, leveraging Graph Neural Networks (GNNs) and attention mechanisms to make context-aware decisions grounded in both users’ social relationships and individual preferences^199^. This method enables personalized and adaptive access control in Social Internet of Things (SIoT) networks, making it well-suited to environments where user behavior and device relationships evolve over time. The CARAC model provides adaptive and fine-grained access control by combining contextual data, mathematical weighting, and fuzzy logic within a game-theoretic framework. This approach enhances safety in high-risk scenarios, outperforming ABAC with minimal impact on performance^200^. In this work^201^, DSA-Block model is proposed as a secure, decentralized access control framework for IoT systems, integrating blockchain and optimization techniques to enable trust-based access delegation and privacy-preserving data sharing. It employs Hyperledger Fabric as the private blockchain platform, leveraging Trusted PBFT for consensus among trusted nodes and HECC for lightweight cryptographic operations. Access requests are filtered using SHA-256 hashing, while Shannon Entropy supports dynamic trust evaluation and user revocation. The model ensures data privacy through Laplace-based differential privacy and selects optimal edge node delegators using the Rock Hyraxes Swarm Optimization (RHSO) algorithm, factoring in trust, energy, and load. The IoT environment and system performance are simulated using NS-3 (v3.26), demonstrating a scalable, attack-resistant, and privacy-aware architecture for secure data sharing and access control.

### Trust management

This section reviews existing trust management approaches, including reputation-based trust, which evaluates trust through past behavior, social trust networks that leverage social relationships, trust propagation that enables transitive trust, hybrid trust that combines multiple sources, and decentralized trust that establishes trust without a central authority, as discussed in the literature.

1. ** Reputation Based System** In trust management, the reputation based system is a method for assessing and measuring the trustworthiness of an enity such as device, user, or a service, based on past interactions and feedback from others in that network. It plays an important role espcially in decentralized environment over centralized authority. To address the issue when VPN collects data through SIoT devices, delivered content might be tampered inorder to degrade QoS and user experience to tackle researchers implemented VMGuard, a four-layer reputation-based incentive framework, to defend against data poisoning attacks in the vehicular metaverse. The framework assesses the trust worthiness of participating Social Internet of Things (SIoT) devices, ensuring reliable data collection and service delivery^76^
2. **Social Trust Network ** By integrating fuzzy logic, trust modeling, network theory, and optimization techniques, this study^202^ introduces a trust-based group decision model that leverages discrete Z-numbers to enhance decision-making in social trust networks.
3. **Trust propagation**
  Trust propagation refers to indirect trust between entities without direct relationships by leveraging trust relationships along network paths. Researchers^203^ have developed SISR, a hybrid trust-aware recommendation system that combines explicit and implicit trust models with latent feature mining. By utilizing trust propagation, SISR extends trust beyond direct relationships, enhancing recommendation accuracy even with sparse trust data. By leveraging concepts from Social Network Analysis (SNA), fuzzy logic, opinion dynamics, and decision theory, the authors^188^ proposed a consensus-reaching model that incorporates a hybrid dynamic trust network along with a trust propagation and aggregation mechanism. These mechanisms ensure that even when decision makers lack direct relationships, trust can still be effectively inferred and aggregated, which is critical in sparse or incomplete networks. The proposed model facilitates reliable trust assessment through the integration of both static social ties and dynamic preference similarities.
4. **Hybrid Trust**
  Hybrid trust management means combining two or more different approaches to evaluate trust between devices in a network especially in Social Internet of Things. To address the challenge of trust evaluation in a heterogeneous Social Internet of Things (SIoT) environment, the authors in^204^ propose a hybrid trust management framework tailored for multi-service SIoT networks. They have considered graph based trust, interaction based trust and human plus device intelligence.The paper introduced AI-SIoT, a hybrid service architecture that integrates heterogeneous IoT devices in smart cities using semantic web technologies and artificial intelligence. By enabling semantic interoperability and AI-based decision-making, the system supports trust-aware service provisioning across diverse environments^205^.
5. **Decentralized Trust** Decentralized trust is a trust management model where trust relationships are established and maintained without relying on a single central authority. Instead, trust decisions are made collectively or based on local information by nodes or entities within the network.This^7^paper conducts an in-depth review of blockchain-based decentralized trust management systems across four IoT classes: IoMT, IIoT, IoV, and SIoT.

### Privacy preserving technique

In this subsection, we discuss three widely adopted privacy preseving techniques such as Differential privacy(used in Healthcare, smarthomes, or social platforms), Privacy preseving data sharing( using Blockchain, homomorphic encryption, or secure APIs) , and minimization(any sensor netowork). These methods applies across the domians are not tied to specific technology. These methods are foundational and versatile, forming core of many SIoT privacy framework. Besides these there are other advanced methods, such as^206^ Zero-Knowledge Proofs(ZKPs)...etc.

1. **Differential privacy**
  This Differential privacy is a mathematical technique that protects individual privacy in datasets while enabling analysis, ensuring that the outcome remains roughly the same whether or not a single individual’s data is included. It’s widely applied in Data Science, AI, and Social Internet of Things (SIoT) to preserve privacy. The proposed^207^ study introduces a solution for data protection, combining Local Differential Privacy (LDP) and Randomized Response (RR) at the user level, with privacy-aware computation techniques (HMM and obfuscation) at the central server level. An additional layer of differential privacy provides extra security. This approach is well-suited for safeguarding data in smart home and smart environment applications. Reference^51^ Proposed a differential privacy preserving solution called as DPSmartCity , an SDN integrated , dynamic privacy preserving mechanism for safe guarding sensitive data in smart city IoT environment.
2. **Annonimization** Anonymization it is a process of removing identifying detials from data to prevent indiviuals from being recognized, it is commonly used to protect privacy in dataset, ensuring personal information cannot be traced back to the specific people. The authors^208^ conducted a comprehensive survey on deidentification, anonymization and psedonymization techniques, evaluating them against smart city specific privacy challenges. When IoT devices communicate with each other over different blockchain networks, it’s difficult to be compatible and private. This article^206^ proposed a privacy protection protocol, using two main techniques, such as Groth16 Zero-knowledge proof, coin mixing technology and virtual external address mapping via generation function, a smart method to move assests across blockchain without revealing their identity.
3. **Privacy preserving data sharing**
  The privacy preserving data sharing is a process that allows data to be shared and analaysed without compramising on its privacy. In^209^, a PP-SVM framework is presented for a privacy-preserving industrial IoT data sharing and analysis, leveraging blockchain-based security and SVM-based machine learning techniques to protect data privacy. The BP3-MTS blockchain-based solution enables secure and private error data sharing for maritime transportation. Advanced sensor-equipped vessels dynamically share GNSS error data with common vessels, allowing them to correct their positioning. This decentralized approach leverages zk-SNARKs, Merkle trees, and Ethereum-based smart contracts to ensure privacy, transparency, and transaction fairness^210^.
4. **Data minimization**
  A privacy preserving princple, in which data collecting, processing and storing only the minimum amount of personal or sensitive data that is required to achieve a specific purpose. To reduce the amount of data transmitted from IoT devices to the cloud, Ref.^189^ proposed a generalized data transmission reduction model. Their approach leverages change detection techniques such as SSIM (for image data), Cloud-to-Cloud distance (for LiDAR point clouds), and absolute difference (for sensor values), along with mathematical formalization to decide when and what data should be transmitted.
5. **Instruction Detection** Intrusion detection means identifying malicious activities or unauthorised access attempts within IoT network. To detect known and unknown attacks, minimise false positive, to enable early and accurate detection of suspicious traffic. To address the security challenges arising from cyberattacks on vulnerable IoT devices, the authors^211^ proposed a framework named HybridChain-IDS. This framework offers an intelligent and privacy-preserving security solution for edge-assisted IoT environments by integrating secure authentication and access control, employing advanced bi-level intrusion detection to reduce false alarms, and enabling real-time threat response through the use of blockchain and graph-based analysis.

### Secure communication protocols

In this subsection, we discuss secure communication protocols such as MQTT over SSL/TLS and DTLS over UDP are widely used protocols in IoT and SIoT systems for secure communication between IoT devices and servers using the MQTT protocol. while these protocols form a foundation for secure communication in SIoT systems. They ensure layered encryption , mutual authentication, and resilience against network level threats such as spoofing, eavesdropping, and DoS attack.

1. ** MQTT with SSL/TLS, DTLS** Instead of TLS/DTLS, resource heavy proptocols for contrained IoT devices the authors^212^ proposed an Ethereum based consortium blockchchain (decentralized brokers), makes MQTT secure and scalabale for real world supply chain by integrating blockchain based trust, OTP based authentication, smart contract automation, while keeping the system lightweight for constrained IoT devices.
2. **Datagram transport layer security over UDP**
  DTLS provides security similar to TLS but operates over UDP, making it ideal for low latency applicaton in SIoT. It safegaurds data exchanges through encryption and authentication while considering constrained networks. The authors^190^ proposed a security enhanced MQTT-SN gateway called as SecGW. This proposed model is a mutithreaded, DTLS-secured gateway which is designed to protect sensor node to gateway hop in MQTT-SN architecture.
3. **COAP Constrained Application protocol with DTLS**
  COAP( Constrainted Application Protocol) is a web-based, light weight protocol designed for contrained devices and networks for IoT devices, allowing efficient communitication protocol over UDP. Combined with DTLS( Datagram Transport Layer Security) it ensures secure bidirectional communication in SIoT environments, protecting against eavesdropping and unauthorized access. The LightCert4IoT model, originally proposed for^91^ securing IoT communications via CoAP over DTLS, can also be extended to Social IoT( SIoT) environments. SIoT, which enables trust based interactions among IoT devices, can benifit from LightCert4IoT’s decentralized authentication mechanism. By eliminating traditional PKI/CA dependencies and leveraging Ethereum based smart contracts for self-signed certificates, SIoT networks can enhance device trust relationships, reduce energy consumption, and secure large-scale IoT deployments while maintaining efficient data.

**Table 14:** Review of security techniques in SIoT.

Ref.	Domain	Purpose	Threat tackled	Core tech	Proposed method	Strengths	Limitations	Future work
^185^	Smart Grid	Quantum-safe auth for meters	Replay, spoofing, quantum threats	RSA + One-Time Pad	Session-based dynamic keying	Quantum-safe, prevents spoofing	Key sync, scaling limits	Optimize for IoT; enable distributed support
^26^	Healthcare	Quantum-safe EHR encryption	Quantum threats, tampering, key misuse	Blockchain, ECDSA, Dilithium	Three-phase hybrid encryption	Strong privacy, quantum safety	Overhead, limited scalability	Combine with AI/IoT for efficiency
^25^	Social IoT	Access control via social trust	Tampering, privacy leaks	Blockchain, Smart Contracts	Relationship-based access rules	Granular access, trust-aware sharing	Latency, complex contracts	Improve dynamic trust updates
^186^	Healthcare	EHR auth and validation	Tampering, unauthorized access	Blockchain, SBT, IPFS, DL	SBT-based selective disclosure	Fast EHR validation, privacy, decentralization	SBTs non-transferable, key loss	Scale and apply cross-industry
^187^	Smart Home Energy	Real-time ABAC via hybrid blockchain	Spoofing, trust abuse	Fabric, Besu, smart contracts, KYC	Modular contracts, trust scoring	Supports zero-trust, adaptive control	Setup complexity, recalibration delay	Add edge AI, FL, DID features
^188^	MAGDM Systems	Enhance consensus via trust	Incomplete trust, low agreement	SNA, trust network, confidence model	Trust feedback via mediator	Better consensus accuracy, real-world proof	Needs accurate input, high compute	Apply to real-time, large-scale cases
^189^	IoT Cloud Offloading	Reduce cloud data transfer load	Latency, energy, bandwidth	Change detection, relational encoding	Only send when change detected	Less data, retains utility	Threshold tuning, depends on cloud decode	Extend to other data types
^190^	Smart Gateway Security	Secure MQTT-SN gateway using DTLS	Eavesdropping, MITM, replay, spoofing, rogue auth, DoS	DTLS 1.2, KMS, multithreading, ClientList	Monolithic, concurrent SecGW with DTLS + mutual auth via KMS	Encrypted SN-GW hop; resilient, scalable, supports multiplexing	Extra memory, slight delay; needs pre-config/KMS	Explore non-GW MQTT-SN security to reduce compute load

## Tools and evaluation metrics in IoT/SIoT environments

This section addresses **RQ**8, focusing on tools and simulation environments commonly used to model, simulate, and evaluate systems within both IoT and SIoT contexts. We review blockchain development tools, AI/ML frameworks, trust, privacy, and security tools, simulation and emulation environments, middleware platforms, as well as ontologies and semantic technologies. Additionally, standard evaluation metrics and validation parameters adopted in recent studies are summarized to highlight common performance assessment strategies. Table 15 presents a summary of tool usage across multiple studies, while Table 16 outlines the evaluation metrics and validation approaches used in recent literature.

### Tools

In this subsection, we provide a comprehensive overview of various categories of tools that support the development and deployment of IoT and Social Internet of Things (SIoT) systems. These tools play a crucial role in simulating network behaviors, enabling intelligent processing, ensuring secure communication, and managing device interoperability across heterogeneous environments. Each of these tool categories contributes to solving specific challenges in IoT and SIoT systems, and the choice of tools largely depends on the application domain, scalability requirements, device capabilities, and the intended use-case scenario. Besides there are other tools too but these are commonly used.

1. Simulation and Emulation Tools—These tools are essential for testing IoT/SIoT architectures, communication protocols, trust models, and network behaviors under different conditions without deploying them in real-world environments. They help validate performance, scalability, and security aspects before actual implementation.
  Cooja(Contiki- NG): A network simulator designed to model low-power wireless protocols, mobility patterns, and trust-based routing mechanisms, enabling the evaluation of IoT protocols in resource-constrained environments and Social IoT (SIoT) trust frameworks. They^91^ developed a blockchain-based authentication mechanism called LightCert4IoT for CoAP/DTLS in IoT environments, leveraging a custom certificate scheme to replace traditional PKI/CA dependency. Their implementation used the Ethereum blockchain (Sepolia testnet) to store device certificates and public keys through smart contracts coded in Solidity, deployed via Remix IDE and managed using MetaMask for secure transaction handling. The Web3 module within their Lightweight Registration Agent (LRA) enabled communication between IoT devices and the blockchain for certificate validation. Performance evaluation using the Cooja–Contiki simulator revealed that LightCert4IoT significantly outperformed conventional X.509-based systems in terms of DTLS handshake time, energy efficiency, and memory usage, highlighting its lightweight and scalable design for resource-constrained IoT settings. This^25^ work proposes a Blockchain-based Federated Learning (BCFL) system deployed across Edge-Fog-Cloud layers to enable secure, decentralized ECG anomaly detection. Using Ganache for smart contract deployment and iFogSim2 for simulation, it compares placement strategies (edge, fog, cloud) and demonstrates that edge deployment achieves superior performance in latency, cost, and energy. The system uses autoencoder models within a Flower-based FL setup, integrating smart contracts to maintain trust and privacy without sharing raw data.
  CCNSim:CCNSim: An artificial intelligence enabled classification, clustering and navigation simulator for Social Internet of Things
  NS-3:A network simulator designed to model low-power wireless protocols, mobility patterns, and trust-based routing mechanisms, enabling the evaluation of IoT protocols in resource-constrained environments IoT/ (SIoT) Social trust frameworks.
  iFogSim /iFogSim2: A fog/edge simulator that emulates fog–cloud architectures while incorporating latency, trust, and access control models, enabling the simulation of resource management strategies and latency-sensitive IoT or SIoT deployments.
    A smart building resource optimization model is designed using real-time data from IoT devices like $\hbox {CO}_{2}$ sensors, CCTV cameras, and light sensors, implemented within a Fog–Cloud architecture and simulated via iFogSim2. The setup is evaluated on a MacBook Pro (2.3 GHz, 8-core Intel Core i9, 16 GB RAM) under fixed and scalable scenarios, with fixed deployments featuring 7 cameras, 17 $\hbox {CO}_{2}$ sensors, and 22 light sensors, while scalable scenarios range device counts from 20 to 200. Device specifications—such as MIPS, RAM, and network parameters—are drawn from manufacturers’ datasheets. Performance is assessed using metrics like energy consumption, latency, and bandwidth utilization, with fog-based configurations in the scalable scenario showing notable improvements: up to 4.35% in energy efficiency, 91.38% in latency reduction, and 38.95% in reduced bandwidth usage^213^.
  YAFS:An event-driven simulator that models dynamic edge/fog deployments, node mobility, and system-level failures, supporting the evaluation of resilience, mobility scenarios, and application behavior in IoT /SIoT environments.
  MATLAB/Simulink:
    A custom modeling tool designed to support mobility modeling, behavioral logic design, and the simulation of trust algorithms, enabling the prototyping of trust frameworks, integration of AI/ML techniques, and exploration of advanced mobility patterns in IoT. Reference^146^ utilized MATLAB R2016a as the core simulation platform to develop and assess their blockchain-enabled digital twin vehicular edge network (DTVEN). The study evaluated performance across various strategies by analyzing task offloading efficiency, latency, energy consumption, and overall network cost. Central to their approach was an Improved Cuckoo Algorithm (ICA) for optimizing task offloading ratios, paired with a greedy strategy to enhance blockchain consensus efficiency. The ICA incorporated sophisticated metaheuristic refinements, including a Levy flight-driven global search, cosine-decreasing discovery probability, Latin Hypercube Sampling for initializing the population, and a hybridization with Golden Sine Algorithm (GSA) and Particle Swarm Optimization (PSO) to boost convergence speed and solution precision. All components were custom-developed and executed within MATLAB, independent of any third-party machine learning or blockchain simulation tools.
2. Middleware and IoT Platforms—Middleware solutions and platforms provide a layer of abstraction between IoT devices and applications, facilitating device integration, data management, interoperability, and service orchestration. These platforms often offer built-in support for device registration, communication protocols, rule engines, and analytics.
  FIRWARE: An open-source IoT middleware platform offering APIs, device management capabilities, and data processing functions, tailored for smart city applications and modular, scalable IoT deployments.
    The framework establishes secure authentication for Digital Twin (DT) ecosystems by employing TinyJAMBU, a lightweight authenticated encryption algorithm, to ensure both confidentiality and device legitimacy during communication. It integrates Eclipse Ditto as the DT platform, utilizing MQTT over TLS/SSL to facilitate secure, bidirectional messaging. Authentication is strictly enforced before any interaction occurs, requiring that the decrypted cipher corresponds to the designated ThingID of the Digital Twin and that the computed encryption tag matches the one received, ensuring precise and reliable identity verification^214^.
    In this work^215^, a Digital Twin Network (DTN) architecture is introduced for Industrial IoT (IIoT) environments, leveraging Eclipse Hono to integrate heterogeneous IoT devices and Eclipse Ditto for managing digital twin entities. The architecture supports real-time synchronization of device states and delivers intelligent services such as predictive maintenance, dynamic resource allocation, security oversight, energy optimization, and adaptive QoS/QoE management. A case study, conducted using the IEEE 802.15.4e TSCH protocol, demonstrates the DTN’s effectiveness in resource scheduling, where policies dynamically adjust in response to changing network conditions through simulation.
  Node-RED: “A flow-based development tool that enables visual programming for interconnecting devices, APIs, and services, supporting rapid prototyping, real-time edge data processing, and streamlined orchestration of lightweight SIoT applications.
  Kaa IoT Platform:An IoT middleware platform offering comprehensive device lifecycle management, seamless data acquisition, and analytics capabilities—tailored for industrial IoT, healthcare applications, and efficient fleet and sensor network operations
  Eclipse Ditto: A digital twin framework that oversees virtual replicas of IoT devices, ensuring real-time synchronization of physical and virtual states—empowering smart factory automation, remote monitoring, and twin-driven control in Social IoT (SIoT) deployments.
  Mainflux :An Industrial IoT platform that provides device management, messaging infrastructure, and robust data security within a microservices architecture—optimized for scalable, secure Social IoT (SIoT) deployments in industrial environments.
3. AI/ML Toolkits for IoT/SIoT Intelligence—The integration of artificial intelligence and machine learning enables IoT/SIoT systems to perform intelligent tasks such as anomaly detection, context-aware decision-making, pattern recognition, and predictive maintenance. These toolkits support training, evaluation, and deployment of AI models either on the cloud, edge, or directly on constrained devices.
  An AI-powered intrusion detection system (IDS) for the Internet of Medical Things (IoMT) is proposed to detect man-in-the-middle (MITM) and spoofing attacks by integrating a secure IoT-edge architecture with machine learning and deep learning techniques. The IDS leverages real-time biometric and network flow data from the WUSTL-EHMS-2020 testbed, with preprocessing done via the ARGUS tool to create a balanced dataset. Eight classification models, including Variational Autoencoders (VAEs), Feedforward Neural Networks (FNN), XGBoost, LightGBM, Random Forest, SVM, and Logistic Regression, were evaluated using accuracy, precision, recall, and F1-score as performance metrics—VAE achieving the highest accuracy at 91.61%. The system was developed using Python libraries such as Pandas, NumPy, Scikit-learn, and Matplotlib, and deployed through a Streamlit-based frontend supporting real-time prediction from eight vital parameters^216^.
  This^217^ study presents a federated intrusion detection system (IDS) that leverages a shallow artificial neural network (ANN) as the global model, collaboratively trained across four virtual clients using the Flower federated learning framework (v1.0.0). The implementation, built in Python, uses Scikit-learn’s MinMaxScaler for preprocessing, and adopts a client–server architecture for distributed training. Experiments ran on a Linux Mint 20.3 Cinnamon setup featuring an Intel Core i7-5960X CPU and 32 GB RAM, with the ToN_IoT and CICIDS2017 datasets supporting both binary and multiclass classification tasks. To evaluate aggregation performance, the study compares FedAvg (as the baseline) with FedAvgM, FedAdam, and FedAdagrad under various training configurations.
  A federated learning based framework is proposed for intrusion and credit card fraud detection, employing IIDNet an enhanced convolutional neural network—as the shared model across 10 clients using the Flower framework. FedAvg acts as the main aggregation method, with FedProx and FedOpt included for performance comparison. Implementation relies on Python with TensorFlow and Scikit-learn, running on Google Colab with NVIDIA GPU support. Two datasets, UNSW-NB15 and a credit card fraud dataset, are used for training and evaluation, with metrics such as accuracy, precision, recall, F1-score, and AUC guiding the assessment of model effectiveness^218^.
  TensorFlow Lite /Micro: A lightweight machine learning inference engine optimized for executing ML models on resource-constrained edge devices, such as microcontrollers—enabling real-time anomaly detection and predictive maintenance in Social IoT (SIoT) environments.
  Federated Learning(e.g., Flower,FedML): A distributed machine learning framework that facilitates collaborative model training across multiple devices while preserving data privacy—empowering personalized intelligence and cross-device learning in Social IoT (SIoT) environments
  Scikit-learn /PyTorch: A general-purpose machine learning and deep learning library offering comprehensive tools for model training and deployment—supporting trust evaluation, pattern recognition, and behavior modeling in Social IoT (SIoT) systems
4. Trust, Privacy, and Security Tools:
  The paper^219^ proposes a secure, cloud-based telemetry framework for drone data that uses hybrid encryption—combining Attribute-Based Encryption (ABE) via OpenABE for session key control and symmetric encryption for efficiency. A Cryptographic Agility Metric is introduced to evaluate encryption performance, access policy enforcement, and system overhead across environments. The framework also compares ABE libraries (Rabe, GoFE, CiFEr, Charm), leverages OpenSSL for RSA-based benchmarking, and references supportive tools like RELIC, MIRACL, and ALE platforms (e.g., LogSentinel, AWS Crypto Tools) to showcase real-world applicability.
  The study^220^ used Ethereum (via Hyperledger Besu) and Hyperledger Fabric as the core blockchain platforms. Hyperledger Caliper was employed to benchmark performance under DDoS attacks. Docker containers hosted network components, while Remix IDE and MetaMask were used for Ethereum smart contract deployment. Chaincode in Hyperledger Fabric was developed using Go, Java, and JavaScript. The benchmarking scripts ran using Node.js, with the entire setup executed on a Debian OS environment through WSL2 on a MacBook Air M1.
  The authors^221^ present a blockchain-enabled extension to IoT platforms by integrating Hyperledger Besu, an enterprise-grade Ethereum client, into the open-source Home Assistant system. Their solution introduces a private blockchain connector, leverages Kafka for data queuing and WebSocket for real-time subscriptions, and uses Solidity smart contracts to securely log IoT transactions. The implementation adopts the QBFT Proof of Authority consensus protocol on Hyperledger Besu and is packaged within a Docker-based environment to facilitate seamless deployment and replication.
  Hyperledger Fabric/ : A permissioned blockchain framework featuring a modular architecture with integrated identity management and privacy-preserving mechanisms—enabling secure and scalable deployments in supply chain IoT, consortium-driven Social IoT (SIoT) systems, and healthcare data exchange
  Hyperledger Besu :An enterprise-grade Ethereum client compatible with both public and private networks, enabling secure and efficient smart contract execution within permissioned Social IoT (SIoT) ecosystems.
  uTrust: A trust management framework that facilitates negotiation, assessment, and policy enforcement across distributed systems—enabling trust-based access control in heterogeneous Social IoT (SIoT) networks
  TruSDN:A blockchain-integrated Software Defined Networking (SDN) security framework that enhances trust, transparency, and access control—supporting secure IoT infrastructure with dynamic routing and policy enforcement.
  OpenABE : An attribute-based encryption (ABE) toolkit that enforces cryptographic access control through attribute-driven policies—enabling fine-grained, secure data sharing in IoT and Social IoT (SIoT) environments.
5. Ontologies and Semantic Tools for SIoT—Ontologies play a significant role in modeling relationships among entities in SIoT, supporting context awareness, semantic interoperability, and trust reasoning. Semantic tools enable the use of shared vocabularies and structured data representation, making interactions more meaningful and automated.
  In this^222^ introduced the Semantic Smart Home System (SSHS), a knowledge-driven home automation framework designed to enhance IoT interoperability and scenario complexity through Semantic Web technologies. The system integrates data from physical and virtual IoT devices using the SAREF ontology and infers actions—like adjusting lighting or activating irrigation—by applying SWRL rules through the Pellet reasoner. It supports scenarios such as energy monitoring, visitor notifications via light signals, and weather-responsive irrigation. Development tools included OWL for device modeling, SWRL and SAREF for semantic rule definition and standardization, and Owlready2 for ontology manipulation within Python, the primary implementation language. Protégé was used for ontology validation, while the OpenWeather API provided real-time environmental data, enabling context-aware automation without vendor lock-in.
  This paper^223^ proposed an OWL- and SWRL-based ontology to classify and detect conflicts among smart home automation rules. Using Protégé, they modeled a comprehensive system that identifies simultaneous execution, chaining, rule redundancy, cross-environmental impacts, and safety violations. The proposed method covers complex interactions often missed in prior models and improves detection coverage across five conflict classes.
  SSN (Semantic Sensor Network): “An ontology standard that formally defines sensors, observations, and associated concepts in a machine-interpretable format—facilitating semantic annotation, interoperability, and automated reasoning in Social IoT (SIoT) systems.
  SAREF:An IoT ontology framework that provides standardized semantic definitions for smart appliances and device interoperability—supporting semantic modeling across smart homes, intelligent buildings, and cross-domain IoT ecosystems.
  IoT-Lite:A lightweight IoT ontology that serves as a streamlined extension of the Semantic Sensor Network (SSN) standard—designed for resource-efficient semantic annotation in constrained Social IoT (SIoT) environments with limited-capacity devices.
  Protege:A graphical user interface (GUI)-based ontology development tool for constructing, modifying, and visualizing ontologies (e.g., OWL)—enabling the design of customized semantic models and reasoning frameworks tailored for Social IoT (SIoT) systems.
6. Blockchain Development Tools for IoT/SIoT—With the rise of blockchain as a decentralized trust infrastructure, development tools such as Ganache, Truffle, Hardhat, and IPFS enable secure data management, smart contract execution, and transparent interactions among IoT devices and users in SIoT environments. These tools support the design and testing of blockchain-based applications tailored to the limitations and needs of IoT systems.
  Ganache : It provides a local, private Ethereum blockchain with pre-funded test accounts and instant mining, allowing for fast and cost-free testing in an isolated environment without relying on a public testnet. The authors They proposed a four-layer architecture (IoT device layer, Edge layer, Blockchain layer, and Application layer) for managing IoT data communication using blockchain and smart contracts. The system is designed using a publish/subscribe model and ensures secure, trustless, and decentralized communication while avoiding direct blockchain interaction by IoT devices to reduce resource consumption^224^.
  Truffle: A comprehensive development toolkit for smart contracts, handling writing, compilation, migration, and testing, with seamless integration with Ganache. They^225^developed a food supply chain management prototype using Ethereum smart contracts, where Ganache was employed as a local blockchain environment to test and deploy contracts. The study demonstrated how Ganache enables controlled testing conditions, including simulated mining, transaction cost tracking, and ether balance updates, validating the deployment and migration of contracts in a secure and isolated setup.
  Remix IDE: A web-based Solidity Integrated Development Environment (IDE) that offers online code, editing, compilation, deployment for Solidity smart contracts, all without requiring local setup. It’s perfect for rapid prototyping, Educational purposes, Debugging small projects . However, it might not be suitable for complex or large-scale projects.
  Hardhat:It provides a development environment & task runner, a modern, highly customizable alternative to Truffle. It facilitates testing, script execution, and contract deployment, with robust plugin support. Widely adopted in professional Ethereum projects due to its greater flexibility compared to Truffle.
  MetaMask: Wallet + Web3 Bridge, a browser extension enabling users to sign transactions, manage accounts, and seamlessly connect to decentralized applications (dApps). Essential for facilitating smart contract interactions through a frontend interface when real users engage via a web browser.
  Web3.js: JavaScript SDK, a JavaScript library designed to connect Node.js or browser-based applications to the Ethereum blockchain. It facilitates transaction signing and smart contract interactions, serving as a bridge between frontend or backend environments and Ethereum. Commonly used alongside tools like Truffle, Hardhat, or MetaMask.
  IFPS: Decentralized File Storage, a peer-to-peer file system that enables off-chain data storage while maintaining on-chain references. Ideal for handling large files, logs, and metadata that are impractical to store directly on the blockchain.

**Table 15:** Tool usage across multiple references.

Tool category/tool name	^224^	^146^	^91^	^222^	^220^	^218^	^215^	^25^	^201^	^138^
**Blockchain development tools**										
Ganache	✓							✓		
Truffle	✓									
Remix IDE			✓		✓					
Hardhat										
MetaMask			✓		✓					
Web3.js			✓							
**AI/ML tools**										
TensorFlow Lite / Micro						✓				
Federated Learning (e.g., Flower)						✓		✓		
Scikit-learn / PyTorch						✓				
**Trust, privacy, and security tools**										
Hyperledger Fabric / Besu					✓				✓	
uTrust										
TruSDN										
OpenABE										
**Simulation and emulation tools**										
Cooja (Contiki-NG)			✓							
NS-3									✓	
iFogSim / iFogSim2								✓		
YAFS										
MATLAB / Simulink		✓								✓
**Middleware and IoT platforms**										
FIWARE										
Node-RED										
Kaa IoT Platform										
Eclipse Ditto							✓			
Mainflux										
**Ontologies and semantic tools**										
SSN										
SAREF				✓						
IoT-Lite										
Protégé				✓						

### Performance metrics and parameters

Evaluation metrics play a vital role in analyzing the performance, reliability, and security of proposed models, architectures, and algorithms. The choice of metrics typically depends on the specific focus of the work—such as trust management, routing protocols, access control, service discovery, or security frameworks—each requiring tailored assessment criteria. In this section we will discuss about some commonly used standard evaluation metrics in IoT/SIoT environment.

1. Trust and Reputation Evaluation :
  Trust Accuracy: How many trust predictions were correct (both positive and negative) out of all predictions.
    $$\textrm{Accuracy} = \frac{TP + TN}{TP + TN + FP + FN}$$
    TP = true positives (correctly predicted trusted);TN = true negatives (correctly predicted untrusted); FP = false positives (incorrectly predicted trusted);FN = false negatives (incorrectly predicted untrusted).
  MAE: Mean Absolute Error (MAE) tells us how far off our trust predictions are, on average. It looks at the difference between what we guessed and what the actual values were, and then averages those differences. Every mistake, big or small, is treated the same.
    $$\textrm{MAE} = \frac{1}{n} \sum _{i=1}^{n} \left| \hat{T}_i - T_i \right|$$
    $n$ is the total number of predictions; $\hat{T}_i$ is the predicted trust score; $T_i$ is the actual (true) trust score; $\left| \cdot \right|$ denotes the absolute value.
  RMSE : Mean Absolute Error (MAE) tells us how far off our trust predictions are, on average. It looks at the difference between what we guessed and what the actual values were, and then averages those differences. Every mistake, big or small, is treated the same.This formula is widely used in evaluating trust models that output continuous trust scores (e.g., values in the range $[0, 1]$) rather than binary labels.
    $$\textrm{RMSE} = \sqrt{ \frac{1}{n} \sum _{i=1}^n \left( \hat{T}_i - T_i \right) ^2 }$$
    Where: $n$ is the number of trust predictions; $\hat{T}_i$ is the predicted trust score; $T_i$ is the ground truth trust score.
  Accuracy, Precision, Recall, F1-score:
    In trust management, entities are typically labeled as Trusted (safe to interact with) or Untrusted (to avoid), and a trust classifier—such as an AI/ML model or a rule-based system—is used to assign these labels. The performance of this classifier is then evaluated using key metrics: accuracy gives an overall sense of how often it’s correct; precision checks whether the system mistakenly trusted untrustworthy nodes; recall measures if it failed to recognize genuinely trustworthy entities; and the F1 score assesses how well it balances precision and recall to ensure reliable trust assessments.
    $$\begin{aligned} \textrm{Accuracy}= & \frac{TP + TN}{TP + TN + FP + FN} \\ \textrm{Precision}= & \frac{TP}{TP + FP} \\ \textrm{Recall}= & \frac{TP}{TP + FN} \\ \textrm{F1}= & 2 \cdot \frac{\textrm{Precision} \cdot \textrm{Recall}}{\textrm{Precision} + \textrm{Recall}} \end{aligned}$$
    $TP$: True Positives — correctly predicted trusted entities; $TN$: True Negatives — correctly predicted untrusted entities; $FP$: False Positives — untrusted entities wrongly predicted as trusted; $FN$: False Negatives — trusted entities wrongly predicted as untrusted.
2. Network and communication performance
  Packet Delivery ratio(PDR): Ratio of successfully delivered packets to total sent.
    $$\textrm{PDR} = \frac{\text {Total Packets Received}}{\text {Total Packets Sent}}$$
  End-to -End Delay: Average latency from source to destination.
    $$\textrm{Delay}_{\text {avg}} = \frac{1}{n} \sum _{i=1}^{n} (t^{\text {recv}}_i - t^{\text {send}}_i)$$
  Throughput:Total successful message delivery over time (bps/kbps).
    $$\textrm{Throughput} = \frac{\text {Total Bits Received}}{\text {Time Taken}}$$
  Message overhead: Additional control or trust messages transmitted in the network.
    $$\textrm{Overhead} = \frac{\text {Control Messages}}{\text {Total Messages}}$$
  Hop Count: Average number of hops between nodes during communication.
    $$\mathrm {Avg\_Hops} = \frac{1}{n} \sum _{i=1}^{n} Hops_i$$
3. Security and Privacy Metrics Used when dealing with identity, access control, and resistance to attacks.
  Attack Detection Rate: Percentage of attacks correctly identified.
    $$\textrm{DR} = \frac{TP}{TP + FN}$$
  False Alarm Rate: Incorrect detection of benign behavior as malicious.
    $$\textrm{FAR} = \frac{FP}{FP + TN}$$
  Resilience to Sybil/On-Off/Bad-Mouthing attacks: Specific to trust systems.
  Entropy: Measures uncertainty or unpredictability in communications (for privacy leakage assessment).
  Anonymity set size: Number of indistinguishable users/nodes for privacy-preserving systems.
4. System and Resource Efficiency: Relevant when SIoT is deployed on edge, fog, or embedded systems:
  Execution Time: Time taken to execute algorithms or protocols.
  Energy Consumption: Especially important for battery-powered IoT nodes.
    $$E = P \cdot t$$
  Memory and CPU Usage: System resource requirements.
    $$\text {CPU Utilization} = \left( \frac{\text {CPU Time}}{\text {Total Time}} \right) \times 100\%$$
  Scalability: Performance as the number of nodes or services increases.
5. Social Relationship Evaluation: Specific to SIoT, where relationships among objects (like ownership, co-location) are used:
  Social Closeness Score: Quantifies relationship strength between devices.
    $$\text {Normalized interaction frequency / duration / proximity} \in [0, 1]$$
  Friendship Ratio: Ratio of direct/indirect socially connected nodes.
  Community Detection Accuracy: Measures accuracy in clustering socially-linked IoT nodes.
    $$\textrm{Accuracy} = \frac{\text {Correctly Clustered Nodes}}{\text {Total Nodes}}$$
6. ML/AI-Based Evaluation(when used): If trust prediction or anomaly detection uses AI/ML:
  ROC Curve and AUC: The ROC (Receiver Operating Characteristic) curve shows the trade-off between True Positive Rate (Recall) and False Positive Rate across different thresholds of a binary classifier. A curve closer to the top-left corner indicates better performance, while the diagonal represents random guessing. The Area Under the Curve (AUC) condenses this into a single score—1.0 means perfect classification, and 0.5 reflects random performance.
    $$\begin{aligned} \text {True Positive Rate (TPR)}= & \frac{TP}{TP + FN} \\ \text {False Positive Rate (FPR)}= & \frac{FP}{FP + TN} \end{aligned}$$
  Confusion Matrix: A 2x2 matrix showing predicted vs actual class counts.
    $$\begin{bmatrix} TP & FN \\ FP & TN \end{bmatrix}$$
  Training Time:Time taken to train the model on data.
  Inference Time: Time taken to make a single prediction (per node/message)

### Performance evaluation review

In this subsection, we review several papers to examine how existing metrics are used to evaluate system performance across IoT and SIoT environments. This analysis helps us better understand and compare different approaches.

**Table 16:** Performance review.

Ref.	Domain	Eval. type	Dataset	Tools used	Lang.	Metrics	Result
^213^	Smart Building	Simulation	iFogSim2 traces	iFogSim2	Java	Energy, Latency, BW	Fog: 91.38% lower latency, 38.95% less BW than Cloud
^25^	SIoT Security	Simulation	16 devs, 10 rel., 21 services	GNN, Blockchain, SciPy stack	Python	Acc., Prec., Trust, Latency, Thrpt.	GNN: 95% acc., 280 Tx/s, 2.2s delay, high sec./priv. scores
^216^	Healthcare IoMT	Testbed	WUSTL-EHMS (16K)	Scikit-learn, ARGUS, Streamlit	Python	Acc., Prec., Recall, F1	VAE: 91.6% acc.; 8 vitals in real-time GUI
^201^	Cloud-IoT Security	Simulation	Synthetic traffic	HECC, HL Fabric, PBFT, DP, RHSO	Python, C++	Acc., Latency, Thrpt.	94% acc., low latency, 80 kb/s throughput
^113^	Smart Home	Simulation (FL)	BoT-IoT, TON-IoT, MQTTset	TensorFlow, Keras, gRPC, TLS	Python	Acc., ROC-AUC, F1	FL: up to 96.5% acc., reduced delay/overhead
^105^	Smart Kitchen IoT	Sim. + Testbed	Custom IR images (13 classes, 1518 aug.)	RPi 3, IR Cam, YOLOv5n, TFLite, Firebase, Android Studio	Python, Java	Prec., Recall, mAP@0.5, F1	mAP@0.5 = 97.1%, Prec. = 91.5%, Recall = 94.6%, Cost = 224
^68^	SIoT Security	Simulation	SWIM + Brightkite (synthetic + real)	Custom Python simulation	Python	AUC, FP, FN, Time, Memory	Higher AUC, lower FP/FN vs. SybilSCAR; similar time; slightly more memory
^21^	Smart City Security	Simulation	CIC-IoT (105 devices, 33 attacks, 7 classes)	Ethereum (Go-Ethereum, Ganache), PySpark, NN	Python, NodeJS	Accuracy, Processing time, Throughput	Acc. 98.4%, up to 99.8% (Spoofing), 0.389s, 4500 tx/s
^22^	IoT / SEaaS	Sim. & Prototype	Synthetic IoT sensing data	Ethereum (SCs), Ganache, Truffle, Remix	Solidity, Python/JS	Latency, Throughput, Gas cost, Scalability	Lat. 1–2s, Thru. 15–20 tx/s, Gas 21k–40k, scalable, secure
^35^	IDS for IoT	Simulation	CICIoT2023 (105 devices, 33 attacks)	ANN + FL (FedAvg) + SHAP	Python	Acc., Prec., Rec., F1, Loss	Train 88.4%, Test 88.2%, Prec. 0.89, Rec. 0.68, F1 0.70

#### Standard evaluation types

In order to ensure consistency across surveyed works, we classify evaluation approaches in IoT/SIoT research into five categories: simulation, prototype/testbed, dataset-based validation, emulation, and analytical/theoretical.

**Table 17:** Standard evaluation type categories in IoT/SIoT research.

Evaluation type	Description/examples
Simulation	Experiments conducted using simulators such as MATLAB, NS-3, OMNeT++, CloudSim, iFogSim, or Google Colab. Typically used for performance studies (e.g., latency, scalability, throughput, energy)
Prototype/testbed	Hardware-based implementations (e.g., Raspberry Pi, Arduino, FPGA, edge/fog nodes, or small-scale IoT deployments). Demonstrates feasibility in realistic IoT/SIoT environments
Real-world dataset	Evaluation performed on public datasets (e.g., UNSW-NB15, CICIDS, IoT-23, UCI IoT datasets) or custom sensor/IoT data collected in the field. Used to validate detection accuracy, trust prediction, etc
Emulation	Virtualized or cloud-based test environments (e.g., Mininet, containerized clusters, digital twins). Offers controlled experiments closer to deployment scenarios.
Analytical/theoretical	Formal analysis, mathematical modeling, security proofs, or purely theoretical validation without experimental deployment

As shown in Table 17, this taxonomy provides a unified lens for comparing diverse studies and clarifying the types of experimental validation adopted. Building on this foundation, the following section summarizes the commonly reported metrics and benchmarking suites that complement these evaluation approaches.

### Summary of standardized metrics and benchmarking suite

To align evaluation practices in IoT/SIoT research, we summarize standardized metric definitions, map tools to evaluation questions, and recommend a minimal benchmarking suite based on recent literature.

**Standardized Metrics.**

- **Latency / Delay:** End-to-end message delay between sender and receiver, reported in milliseconds. Recommended to present as percentiles (50th, 90th, 95th) to capture both median and tail performance.
- **Throughput:** Number of successful transactions or messages per unit time, typically expressed as transactions per second (tx/s) or packets per second (pps). Indicates the system’s processing capacity under load.
- **Scalability:** Maximum number of IoT/SIoT nodes supported while maintaining acceptable latency ($<200$ ms) and throughput. Often plotted as performance versus node count.
- **Trust / Intrusion Detection Accuracy:** Ratio of correct predictions (trusted/untrusted) to total predictions:
  $$\text {Accuracy} = \frac{TP + TN}{TP + TN + FP + FN}$$
  where *TP* = true positives, *TN* = true negatives, *FP* = false positives, and *FN* = false negatives. Other measures may include precision, recall, and F1-score.
- **False Positive Rate (FPR):** Fraction of benign events incorrectly classified as malicious:
  $$\text {FPR} = \frac{FP}{FP + TN}$$
  A low FPR is critical in SIoT, as frequent false alarms degrade trust.
- **Energy Consumption / Processing Overhead:** Energy usage per device (mJ, J, or battery %), along with computational load (CPU time, memory footprint). Reported per operation or per message to compare lightweight versus heavy mechanisms.
- **Availability / Reliability:** Percentage of uptime or successful service delivery under stress/failure scenarios:
  $$\text {Availability} = \frac{\text {Uptime}}{\text {Total Time}} \times 100\%$$
  Reliability may also be reported as Mean Time Between Failures (MTBF) or resilience against attacks/failures.

**Tool-to-Metric Mapping.**

- **NS-3:** Suitable for protocol-level latency, routing overhead, and throughput.
- **iFogSim/iFogSim2:** Used for resource placement, energy–latency trade-offs, and scalability analysis.
- **Ganache/Truffle:** Applied for blockchain auditability, transaction delay, and smart contract cost.
- **Scikit-learn, PyTorch, Flower:** Commonly used for trust accuracy, anomaly detection, and ML-driven evaluation.

**Minimal Benchmarking Suite (Recommended).** For comparability across SIoT studies, we recommend the following minimal set:

1. Latency percentiles (50th and 95th).
2. Trust accuracy with false positive rate (FPR).
3. Scalability evaluation up to $\sim$500 nodes.
4. Auditability: smart contract cost and transaction delay.
5. Availability (% uptime under stress/failure).

**Typical Ranges from Literature.**

- Latency: 20–200 ms, with fog-based deployments often $<50$ ms.
- Trust Accuracy: 85–97% depending on dataset and method.
- Scalability: 50–500 nodes in most simulation and testbed studies.
- Availability: Above 98%, with blockchain-based approaches approaching 99.9%.
- Energy Efficiency: Up to 4–5% gains vs. baseline IoT systems.
- Bandwidth Saving: 38–40% reduction through fog/blockchain offloading.

Representative ranges are derived from multiple studies in recent literature ^25,113,201,213,216^. This summary provides a concise benchmarking reference, ensuring that IoT/SIoT evaluations are more systematic, replicable, and comparable across future studies.

## Conclusion and discussion

SIoT represents a transformative shift in how smart objects interact and collaborate. By bridging the physical and social worlds, SIoT opens new frontiers for context-aware services, decentralized intelligence, and trustworthy cyber-physical systems. Continued interdisciplinary research and real-world experimentation will be key to realizing the full potential of socially driven IoT environments. Based on this literature review, future researchers and system designers can benefit from a comprehensive understanding of existing security techniques, modular technology integration strategies, evaluation tools, and performance parameters. These insights can serve as a foundation for designing and implementing secure, scalable, and intelligent SIoT systems. In future work, we aim to leverage these findings to develop a practical SIoT implementation capable of mitigating ongoing security threat and addressing emerging attack vectors.

### Limitations and future work

While this survey provides a comprehensive review of SIoT security, several limitations and open challenges remain that point toward future research directions.

**Limitations of this survey:**

- **Selection and coverage:** Although we systematically surveyed literature from 2014 to 2025 across major databases (IEEE, ACM, Springer, Elsevier, MDPI), niche or regional studies may be underrepresented.
- **Scoring subjectivity:** The ($\checkmark$/ $\star$/✗) rubric and inter-rater checks reduce bias, but the evaluation of tools and performance metrics inevitably involves a degree of subjectivity.
- **Comparability of results:** Despite collating performance metrics and tools, the lack of standardized benchmarking frameworks across studies makes it difficult to directly compare reported outcomes.
- **Integration scope:** Although several studies have implemented blockchain, edge/fog, and AI/ML techniques for SIoT, most efforts are confined to prototypes, conceptual designs, simulations, or controlled testbeds. Evidence of robust, large-scale real-world deployments is still scarce.

**Future research directions (derived from gaps identified in Sections III–VIII):**

- **Lightweight dynamic trust models (Section** “Key research directions in the SIoT”**, RQ**4**):**
  Existing trust mechanisms are often static or computationally heavy; adaptive, lightweight trust strategies are needed for resource-constrained SIoT nodes.
- **Privacy-preserving service discovery (Section** “Key research directions in the SIoT”**, RQ**4**):** Current discovery mechanisms frequently expose sensitive identity or location attributes; future protocols should enable secure interaction without disclosure.
- **Cross-domain interoperability and unified policies (Section** “Key research directions in the SIoT”**, RQ**4**):** Standardized access-control and trust policies across heterogeneous SIoT domains remain largely unexplored, limiting secure interoperability.
- **Explainable and accountable AI/ML integration (Section** “Key research directions in the SIoT” & **Subsection** 6.3.1 **, RQ**4**):** ML-based anomaly detection and trust prediction lack transparency; incorporating explainable AI (e.g., SHAP, LIME) is crucial for accountable decision-making.
- **Benchmarking and reproducibility frameworks (Section** “Tools and evaluation metrics in IoT/SIoT environments”**, RQ**8**):** Reported evaluation parameters (latency, scalability, trust accuracy, energy overhead) vary widely; standardized datasets, toolkits, and parameter ranges are needed.
- **Dataset and trace availability (Section** “Survey methodology”**, RQ**2**):** SIoT-specific datasets and reproducible traces (e.g., discovery logs, attack scenarios) are scarce; curating open datasets would strengthen comparability and validation.

To enhance transparency and reuse, we provide two machine-readable CSV files as supplementary material: **Supplement S1** (corpus_225.csv) contains the corpus of the **225** included studies (Year; Title; Venue; Type; Access), and **Supplement S2** (table1_related_surveys.csv) contains the data underlying Table 1 (Reference No.; Year; Security requirements; Attack types/applications; Security protocols; Security techniques; Technology integration; Evaluation tools; Performance metrics). These artifacts support reproducibility, enable independent verification, and provide a foundation for extended SIoT research.

## Supplementary Information

Supplementary Information 1.

Supplementary Information 2.

## Data Availability

Derived data supporting the findings of this study are available as supplementary material: S1 ( corpus_225.csv) and S2( table1_related_surveys.csv). These files contain metadata and annotations extracted from the cited literature; no new experimental datasets were generated.
